# AmplificationTimeR: an R package for timing sequential amplification events

**DOI:** 10.1093/bioinformatics/btae281

**Published:** 2024-04-24

**Authors:** G Maria Jakobsdottir, Stefan C Dentro, Robert G Bristow, David C Wedge

**Affiliations:** Division of Cancer Sciences, University of Manchester, Manchester Academic Health Science Centre, Manchester M13 9PL, United Kingdom; Christie Hospital, The Christie NHS Foundation Trust, Manchester Academic Health Science Centre, Manchester M20 4BX, United Kingdom; Division of AI in Oncology, German Cancer Research Center (DKFZ), Heidelberg, Germany; Christie Hospital, The Christie NHS Foundation Trust, Manchester Academic Health Science Centre, Manchester M20 4BX, United Kingdom; Division of Cancer Sciences, University of Manchester, Manchester Academic Health Science Centre, Manchester M13 9PL, United Kingdom; CRUK Manchester Institute and Manchester Cancer Research Centre, Manchester M20 4GJ, United Kingdom; Christie Hospital, The Christie NHS Foundation Trust, Manchester Academic Health Science Centre, Manchester M20 4BX, United Kingdom; Division of Cancer Sciences, University of Manchester, Manchester Academic Health Science Centre, Manchester M13 9PL, United Kingdom; Christie Hospital, The Christie NHS Foundation Trust, Manchester Academic Health Science Centre, Manchester M20 4BX, United Kingdom

## Abstract

**Motivation:**

Few methods exist for timing individual amplification events in regions of focal amplification. Current methods are also limited in the copy number states that they are able to time. Here we introduce AmplificationTimeR, a method for timing higher level copy number gains and inferring the most parsimonious order of events for regions that have undergone both single gains and whole genome duplication. Our method is an extension of established approaches for timing genomic gains.

**Results:**

We can time more copy number states, and in states covered by other methods our results are comparable to previously published methods.

**Availability and implementation:**

AmplificationTimer is freely available as an R package hosted at https://github.com/Wedge-lab/AmplificationTimeR.

## 1 Introduction

The landscapes of cancer genomes are often marked by gains and losses of DNA that occur throughout the lifetime of the tumour, affecting genes involved in cancer. As sequencing of cancer samples has become more affordable and widespread, the field of cancer genomics and tumour evolution has sought to assess not just which copy number alterations are important in cancer, but also whether the time and order in which they occur matters. A number of approaches for timing the occurrence of events relative to each other across a population, based on sports statistics, have previously been published, such as the Plackett–Luce model (Ansari-Pour *et al.* 2021, Katz-Summercorn *et al.* 2022) or the Bradley–Terry models (Gerstung *et al.* 2020). These methods typically order events within a sample based on cancer cell fractions before inferring a general order of events across a cohort. However, as the primary focus of these methods is to order events across a population rather than to time them, these models do not produce timing estimates for individual events occurring in each sample. This results in a loss of resolution and a lack of distinction between single copy gains and gains of multiple copies, or “focal” amplifications, the latter of which have been identified as a driving force in cancer progression (Ulz *et al.* 2016). The question remains whether the individual events that make up high level amplifications occur at intervals during evolution of the cancer genome or in close temporal proximity, and whether different patterns of amplification result in different outcomes. Timing of individual events in cancer is important as it informs our understanding of cancer biology. The ability to discriminate between early and late events may inform our approach to early detection of cancer, and discrimination between precancerous and invasive lesions (Durinck *et al.* 2011).

Tools such as MutationTimeR (Gerstung *et al.* 2020) and cancerTiming (Purdom *et al.* 2013) have been developed to address the timing of individual gains, based on principles proposed by Durinck *et al.* (2011). These tools combine copy number calls with the multiplicity (number of chromosomes on which a mutation is found) of the mutations within the amplified copy number segment. In short, mutations occurring before an amplification or whole genome duplication (WGD) event will be present on more than one chromosome copy after the event, whereas mutations occurring post gain/WGD will only be present on one copy. This logic can be extended to time higher order amplifications as well. However, MutationTimeR and cancerTiming restrict their timing to only the first two gains or to events with a maximum total copy number of 5, respectively.

Here, we propose AmplificationTimeR, an R package specifically designed to time sequential or focal amplification events in cancer. AmplificationTimeR is able to time individual events for copy number states up to 10 + 1 without whole genome duplication, and 10 + 2 with whole genome duplication.

## 2 Approach

### 2.1 Timing gains

#### 2.1.1 Principle

**Figure 1.**
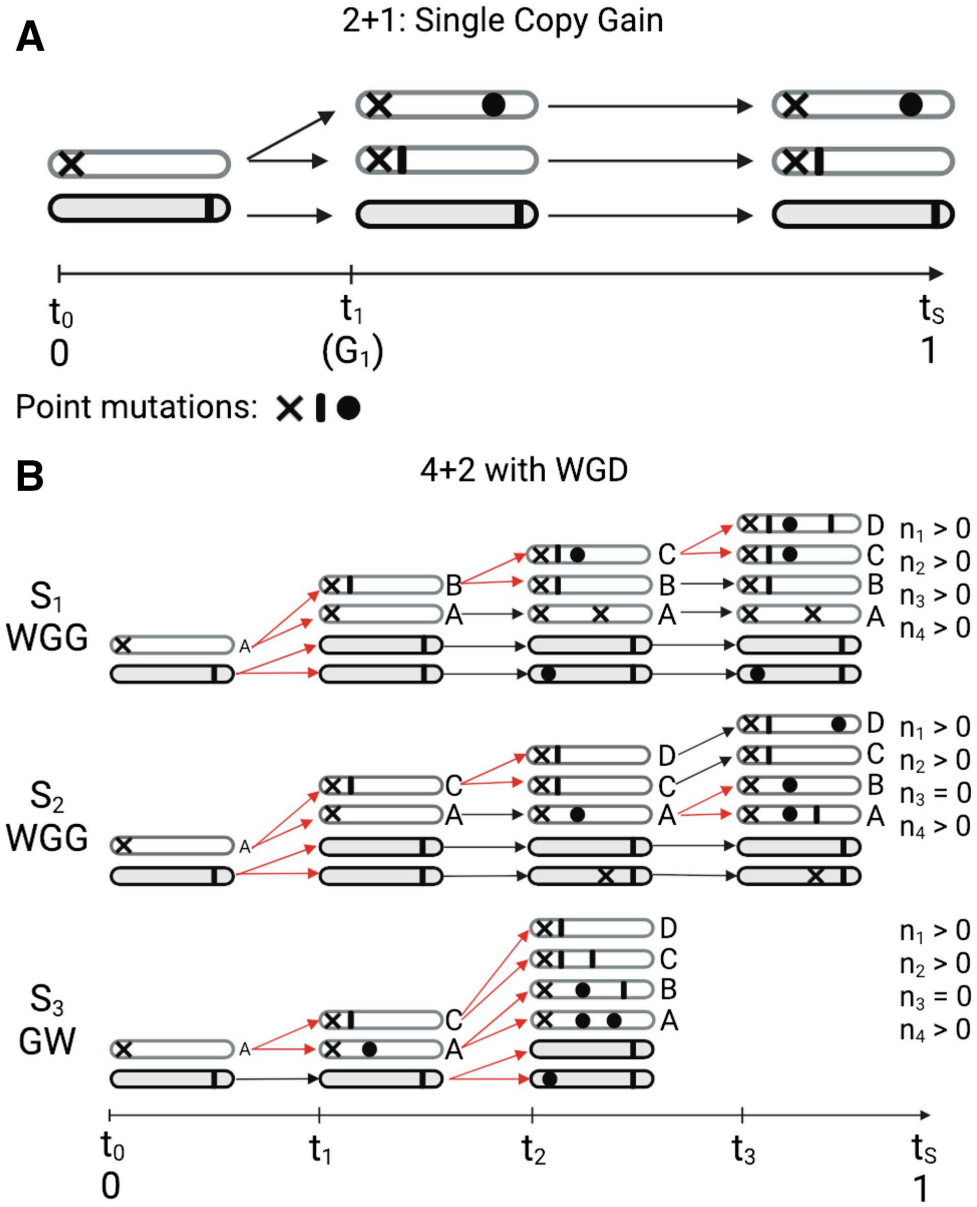
Principle behind timing amplifications. (A) A schematic representation of a single copy gain. Symbols represent point mutations occurring both before and after the depicted chromosomal gain. $t_{0}$ represents tumour initiation, $t_{1}$ or $t_{G1}$ represents the time of the first gain, and $t_{S}$ represents the time of tumour sampling. Mutations occurring before $t_{1}$ are duplicated, and thus present on two chromosome copies. Mutations occurring after $t_{1}$, or on the unaffected chromosome, are present on one chromosome copy. (B) A schematic representations of three scenarios ($S_{1}$, $S_{2}$, and $S_{3}$) leading to a copy number state of 4+2 in a whole genome duplicated sample. W and G are used to refer to whole genome duplication (WGD) and gain, respectively. In $S_{1}$ a WGD event is followed by two sequential gains of the same chromosome, in which case mutations are expected to occur in all multiplicity states in the set {1,2,3,4}. However, if no mutations of multiplicity three are observed, this suggests that the order of events may be a single gain followed by a whole genome duplication ($S_{3}$), which can be timed, or a whole genome duplication followed by gains of two separate chromosomes ($S_{2}$), which cannot be timed. Red lines indicate gain or WGD events. Image created with BioRender.com.

Our work follows the principles previously proposed in Durinck *et al.* (2011). As demonstrated in Fig. 1A, mutations that precede a gain event (occurring between $t_{0}$ and $t_{1}$) will be present on two chromosome copies at the time of sampling ($t_{S}=1$). However, mutations occurring on the chromosome that is not gained, and mutations occurring after the gain, will only be present on one chromosome copy. Thus, if we represent the number of mutations on two chromosome copies as $n_{2}$ and the number of mutations on one chromosome copy as $n_{1}$, separating this into $n_{1G}$ and $n_{1B}$ for mutations arising from the gained chromosome copy (the chromosome with grey outline - $n_{1G}$ in Fig. 1A) and the non-gained copy (the chromosome with black outline - $n_{1B}$ in Fig. 1A), respectively, one can derive the following equations, where *m* represents a constant mutation rate:

(1) $$\begin{array}{c} \begin{array}{c} n_{1G}=2m(1\;-\;t_{1})\\ n_{1B}=m\;\times \;1\\ n_{2}=m(t_{1})\\ \text{Combining to get:}\\ n_{1}=m(3\;-\;2t_{1})\\ n_{2}=mt_{1}\\ \text{Solving for}\;t_{1}:\\ t_{1}=\frac{3n_{2}}{n_{1}\;+\;2n_{2}} \end{array} \end{array}$$

This logic can be extended to calculate the time points of individual gains and whole genome duplications for higher order copy number states with the notation $n_{k}$ used to describe the number of mutations of multiplicity *k*, and $t_{x}$ representing each time point *x*. However, some restrictions apply. In particular, allele specific copy number is an absolute requirement, as the order of events cannot be inferred from total copy number data. In addition, unless a sample has undergone whole genome duplication (WGD), a minor copy number state of two will preclude timing, as the allelic origin of each mutation cannot be determined. This results in a system of equations with no unique solution.

Once WGD events are accounted for, higher copy number states (>3 + 2) can be achieved through different combinations of gains (G) and WGD (W), demonstrated in Fig. 1B, which depicts different routes that lead to a copy number state of 4 + 2. First, the order of events is inferred based on the presence or absence of specific multiplicity states. For example, in scenario 1 ($S_{1}$) of Fig. 1B, a WGD event is followed by two sequential gains of the same chromosome (WGG), resulting in multiplicity states in the set {1,2,3,4}. However, if a copy number state of 4 + 2 is reached through a gain followed by a WGD (WG; $S_{3}$ in Fig. 1B), no mutations of multiplicity 3 can be present. Second, after determining the likely order of events based on the available multiplicity states, each event is timed using the multiplicity data in combination with a set of equations specific to the inferred order.

It is also possible for a state of 4 + 2 to be reached without mutations of multiplicity 3 present in cases where different chromosomes are gained following the WGD ($S_{2}$ in Fig. 1B), as opposed to sequential gains of the same chromosome ($S_{1}$ and $S_{3}$ in Fig. 1B). Gains of different chromosome copies ($S_{2}$ in Fig. 1B) result in a system of timing equations with no unique solution. Thus, we are only able to confidently time scenarios in which the same chromosome copy has been sequentially gained. In cases where absence of specific multiplicity states can be explained by several different combinations of events, we choose to time the order of events featuring the fewest events as the most parsimonious scenario. Please see the Supplementary Text for additional equations and copy number states and scenarios timed by AmplificationTimeR.

Losses affecting gained chromosomes are not considered in the model, as their occurrence cannot be inferred from the available multiplicity data, and their inclusion results in a less parsimonious order of events based on the number of events. Segments in which the minor allele has been lost and the major allele gained can be timed, though the order and time of the loss cannot be determined, and thus is not reported.

#### 2.1.2 Multiplicity

AmplificationTimeR is designed to be compatible with copy number and multiplicity output produced by Battenberg (Nik-Zainal *et al.* 2012) and DPClust (Bolli *et al.* 2014), respectively. DPClust uses the binomial distribution to model the expected fraction of mutated reads for each possible multiplicity state and compares this to the observed fraction of mutated reads in the sequencing data [$f_{s}$; Bolli *et al.* (2014)]. A multiplicity value is then assigned to each mutation using the maximum likelihood (Bolli *et al.* 2014). In cases where the highest copy number state at the locus of interest is called as subclonal, AmplificationTimeR rounds up the non-integer multiplicity values produced by DPClust to the nearest integer using the ceiling function in R. This allows us to time the final gain event, even if it has only occurred in a fraction of the cells.

We note that, like AmplificationTimeR, cancerTiming uses a binomial distribution to model allele frequencies, whereas MutationTimeR uses the Beta-binomial distribution.

#### 2.1.3 Confidence intervals

AmplificationTimeR provides timing estimates based on observed data, as well as a mean timing estimate based on bootstrap re-sampling. For each segment, we re-sample, with replacement, an equal number of mutations and their multiplicities as observed in the original set of mutations found in the segment. We then produce a timing estimate using this re-sampled data, and repeat the process of sampling and timing 500 times, before calculating the mean timing estimate for each event. In addition, we give 95% confidence intervals based on this data.

Note, while cancerTiming and MutationTimeR also provide confidence intervals based on a bootstrap resampling approach, both of these tools take total read coverage and the number of reads containing a specific variant into account. As AmplificationTimeR makes use of multiplicity estimates provided by DPClust, the uncertainty contributed by the estimation of multiplicity is not directly taken into account in the current implementation of AmplificationTimeR.

#### 2.1.4 Assumptions

There are a number of assumptions at the core of AmplificationTimeR and similar tools, some of which have been alluded to earlier in this manuscript. We restate them here for convenience:

- Mutation rate *m* is constant
- Each site in the genome is only mutated once
- The likelihood of acquiring a mutation on each chromosome copy is equal
- If multiple different combinations of gains and losses can explain a specific copy number state and associated multiplicity states observed, the most parsimonious solution, i.e. the one with the fewest events, is selected for timing
- Losses of the major allele are not accounted for in the model
- In cases where clonal and subclonal gains are present, the lower copy number state is considered to be ancestral to the higher state
- Except in the case of whole genome duplication, only one copy is gained at each time point
- In the case of WGD, it is assumed that WGD occurs at one time point, with concurrent gain of both chromosome copies
- WGD occurs only once

#### 2.1.5 Clock-like mutations

In order for timing estimates from AmplificationTimeR, MutationTimeR or cancerTiming to correspond to chronological time, which allows the comparison of timing estimates between patients, it is required that mutations occur at a constant rate over time. However, cancerTiming and MutationTimeR make no mention of using mutations with clock-like properties, implying that all mutations in a segment be used for timing. As part of AmplificationTimeR, users are able to provide the tool with all mutations in a segment, which will then be filtered for C > T mutations at CpG sites, or mutations attributed to the SBS1 and SBS5 mutational signatures, previously established to have clock-like properties (Alexandrov *et al.* 2015). Should they choose to, users can override these filters at their own risk.

## 3 Results

### 3.1 Timing comparison

#### 3.1.1 Comparison with available tools

To compare the performance of AmplificationTimeR with MutationTimeR and cancerTiming we ran all three methods on a subset of data from the PanCancer Analysis of Whole Genomes (PCAWG) consortium (The ICGC/TCGA Pan-Cancer Analysis of Whole Genomes Consortium 2020). We timed MYC amplification in Ovarian cancers (OV; gained in 102/111 samples including gains by WGD and CN-LOH) and Breast cancers (BRCA; amplified in 156/196 samples, including gains by WGD and CN-LOH). These amplifications were selected due to their high incidence and variation in major copy number achieved.

For both the BRCA and OV samples, AmplificationTimeR was able to time the most MYC gains, followed closely by MutationTimeR (Table 1, Supplementary Fig. S1). cancerTiming timed by far the fewest samples in both sets. While MutationTimeR performed well by this metric, we note that MutationTimeR only provides a timing estimate for the first two gain events that have occurred, whereas AmplificationTimeR provides a time for each gain or whole genome duplication, up to a maximum of nine time points, in its current state. We also note that the outputs of MutationTimeR are incomplete when timing subclonal gains, in that when provided with segments describing two subclonal gains of the same segment MutationTimeR will only time the ancestral subclone (Gerstung *et al.* 2020), whereas AmplificationTimeR will also time the subclone with the highest copy number state, under the assumption that it is derived from the subclone in the lower copy number state.

**Table 1.** Number of samples for which at least one point was timed, out of a total of 156 BRCA and 102 OV samples featuring a gain of some sort.^a^

	Mutations	AmplificationTimeR		MutationTimeR		cancerTiming	
		BRCA	OV	BRCA	OV	BRCA	OV
1	All	**107**	**78**	85	59	39	27
2	SBS	**86**	**54**	75	52	29	19
3	C > T	**78**	43	70	**49**	26	15

a Boldface indicates the highest number of samples timed for each combination of tools, tumour types, and mutation types. “SBS” indicates timing using only mutations attributed to mutational signatures SBS1 and SBS5. “C > T” indicates timing using only C > T mutations at CpG sites. “All” indicates the use of all available mutations for timing.

**Figure 2.**
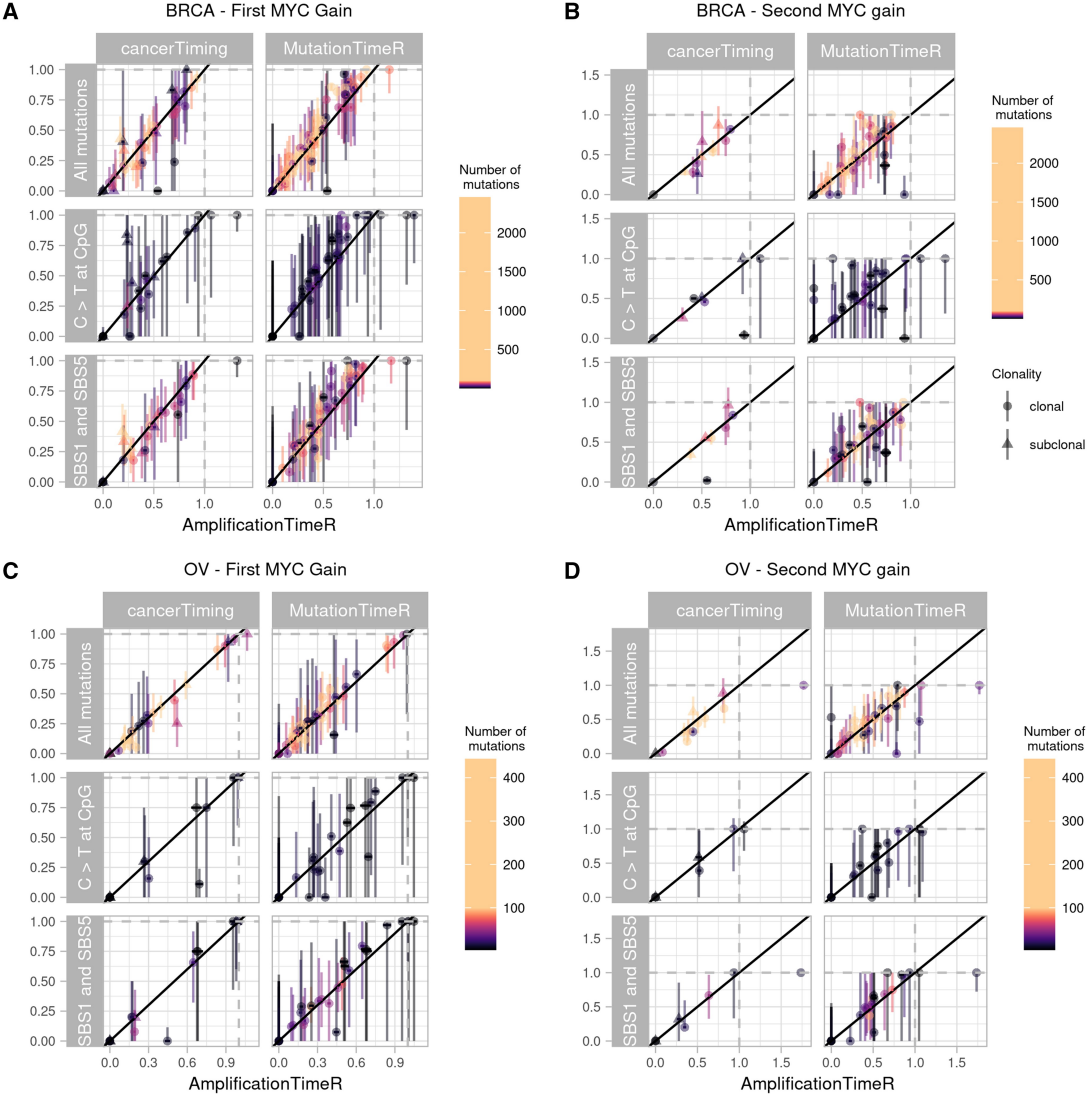
Comparison of first and second MYC gain ($t_{1}$ and $t_{2}$) in BRCA and OV cancers using available timing methods and mutation types. Point shapes indicate whether the timed gain was clonal or subclonal, and colour indicates the number of mutations used. Vertical bars represent the 95% confidence intervals provided by cancerTiming and MutationTimeR, whereas horizontal bars represent the 95% confidence intervals provided by AmplificationTimeR. “All mutations” represents timing estimates derived using all unfiltered mutations, “C > T at CpG” represents timing estimates calculated using only C > T mutations at CpG sites, and “SBS1 and SBS5” represents timing estimates generated using only mutations identified as belonging to mutational signatures SBS1 and SBS5. (A) First MYC gain in BRCA samples. (B) Second MYC gain in BRCA samples. (C) First MYC gain in OV samples. (D) Second MYC gain in OV samples.

As all three methods are able to time at least two events, we compared the first and second time points estimated by AmplificationTimer with those produced by MutationTimeR and cancerTiming (Fig. 2).

Estimates of $t_{1}$ were very highly correlated between the tools for both tumour types (Supplementary Tables S1 and S3). While still significant and highly correlated, the correlation between $t_{2}$ estimates was slightly lower in some comparisons, particularly when comparing MutationTimeR with AmplificationTimeR for BRCA samples (Supplementary Tables S2 and S4). We propose that the slight decrease in correlation is due to MutationTimeR applying a generalized equation to time events whereas AmplificationTimeR makes use of individually derived equations for each copy number state and order of events. Correlation of estimates of $t_{2}$ remained high between AmplificationTimeR and cancerTiming, though based on few samples.

#### 3.1.2 Comparison with simulated data

**Figure 3.**
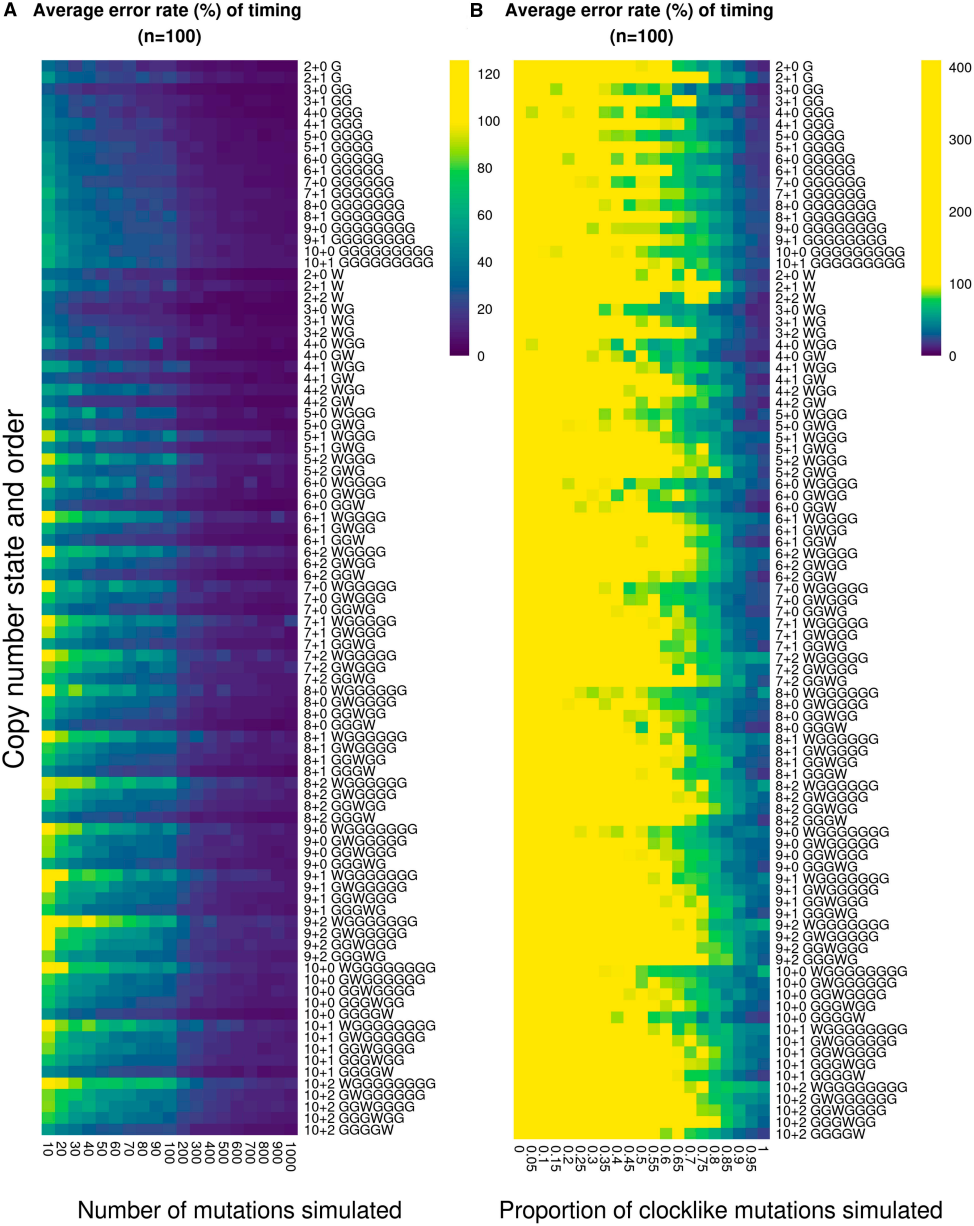
Average error rate of time points calculated from simulated data, expressed as a percentage. *n* = 100 randomly simulated sets of time points for each order, copy number state, and condition. Colour scales are capped at 100% error rate to allow better visualization of lower percentages. (A) Average error rate of calculated time points when varying the number of simulated mutations. (B) Average error rate of calculated time points when varying the proportion of clock-like mutations simulated.

Due to low sample numbers when segments were grouped by copy number state, the lack of ground truth for timing and event orders, and the limited data for comparing higher time points with MutationTimeR and cancerTiming, we also tested AmplificationTimeR’s performance using simulated data (see Supplementary Text for detailed methods). For each copy number state and order of events considered in AmplificationTimeR we randomly generated a set of time points and corresponding multiplicity values for different numbers of mutations, which were then used to assess AmplificationTimeR’s average timing error rate (Fig. 3A) and AmplificationTimeR’s ability to identify the correct order of events (Supplementary Fig. S2A). 100 independent simulated scenarios were generated for each combination of copy number and event order.

We found that AmplificationTimeR’s error rate decreased with increasing number of mutations generated for timing, highlighting the importance of this parameter for the reliability of timing estimates (Fig. 3A). The number of mutations used was also particularly important when assessing AmplificationTimeR’s ability to infer the correct order of events, with lower mutation numbers resulting in frequent mistakes for higher copy number states with many events (Supplementary Fig. S2A). Misidentification of the event order will affect the equations used to time the events, partly explaining the increased error rate seen for higher copy number states with the maximum number of events. The error rate and identification of correct timing order are particularly affected by mutation number in segments with many events, as these require many multiplicity states to be present, which may not be adequately captured at low mutation numbers. For example, correct identification of a copy number state of 10 + 2 resulting from sequential gains of the same chromosome with an event order of WGGGGGGGG requires all multiplicity states from 1 to 10 to be present. For a segment with 10 mutations we would only expect to capture all multiplicity states if the gains and WGD occurred at perfectly even intervals with a consistent mutation rate high enough to result in a mutation between each gain. However, there is no evidence to suggest that gain events happen at regular intervals, highlighting the difficulty of accurate timing with limited mutation numbers. This limitation affects all such timing methods.

**Figure 4.**
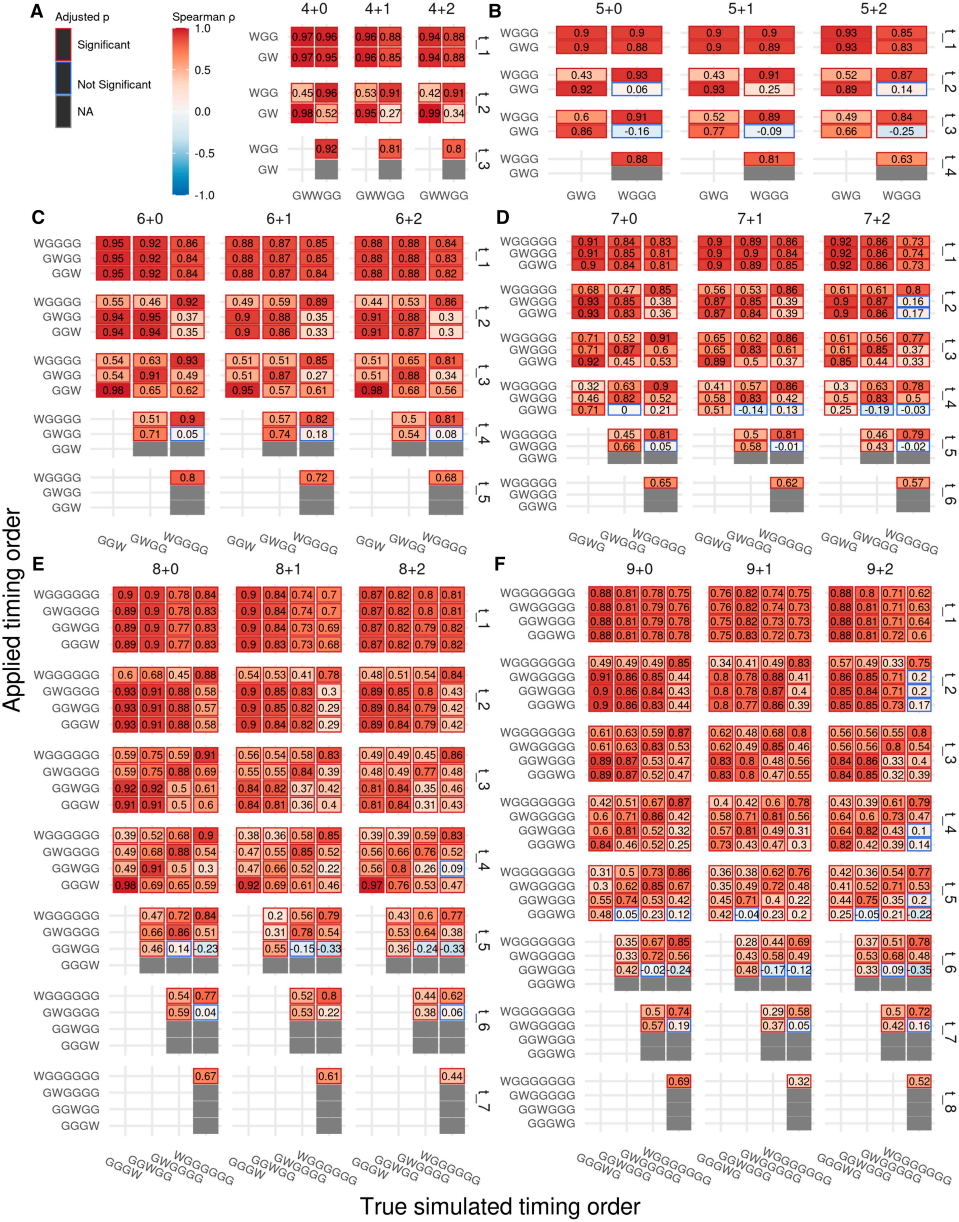
Spearman correlation between the simulated and calculated time for each time point when timed using all possible equations and event orders for timeable copy number states from 4 + 0 to 9 + 2 (A–F). Results for 10 + 0, 10 + 1, and 10 + 2 are shown in Supplementary Fig. S3. The trailing diagonal represents the correlation between time points calculated using the same order of events from which the data were simulated. *n* = 100 randomly simulated sets of time points for each order from which a set of 100 mutations was simulated for timing. Cell colours and numbers indicate the Spearman ρ. Cell borders indicate Benjamini–Hochberg adjusted *P*-values relative to an α of 0.05. Grey cells and cell borders indicate comparisons for which correlation could not be calculated due to differences in the number of time points available.

To further investigate the effects of applying incorrect timing equations, we created another set of simulations. For each copy number state and event order we randomly generated a set of time points and corresponding multiplicity values based on our timing equations (timing equations and detailed methods are described in the Supplementary Text). We then applied all available timing equations for the respective copy number state to the data, including the timing equations corresponding to the order of events from which the data were simulated, and calculated the Spearman correlation between the simulated and calculated time points. The results of this analysis are depicted in Fig. 4 and Supplementary Fig. S3, with the trailing diagonal representing the correlation between matched simulated and applied timing orders, and points off the trailing diagonal representing mismatched event orders.

We found that the correlation between simulated and calculated time points was high when the correct timing equation was applied, though this decreased with later time points for samples with high copy numbers. We also observed that $t_{1}$ was highly correlated across all copy number states and event orders, regardless of whether the correct timing equation was applied or not, and that there was generally moderate to high correlation between all but the final two time points in scenarios featuring many events. Finally, we also observed that applying equations featuring WGD as an earlier event than simulated (e.g. identifying a copy number state of 9 + 0 deriving from GGWGGG as GWGGGGG; upper left triangle of the correlation plots in Fig. 4) resulted in higher correlation coefficients than applying equations featuring WGD as a later event than simulated (e.g. identifying a copy number state of 9 + 0 deriving from GGWGGG as GGGWG; bottom right triangle of the correlation plots in Fig. 4). Identifying WGD as occurring earlier in the order of events than it actually did is not possible in real data, as this requires multiplicity states that would be absent given the true order of events to be present. Thus, when applied to real-world data WGD will only ever be identified as occurring later in the order of events, thus resulting in less accurate timing estimates.

### 3.2 AmplificationTimeR results

**Figure 5.**
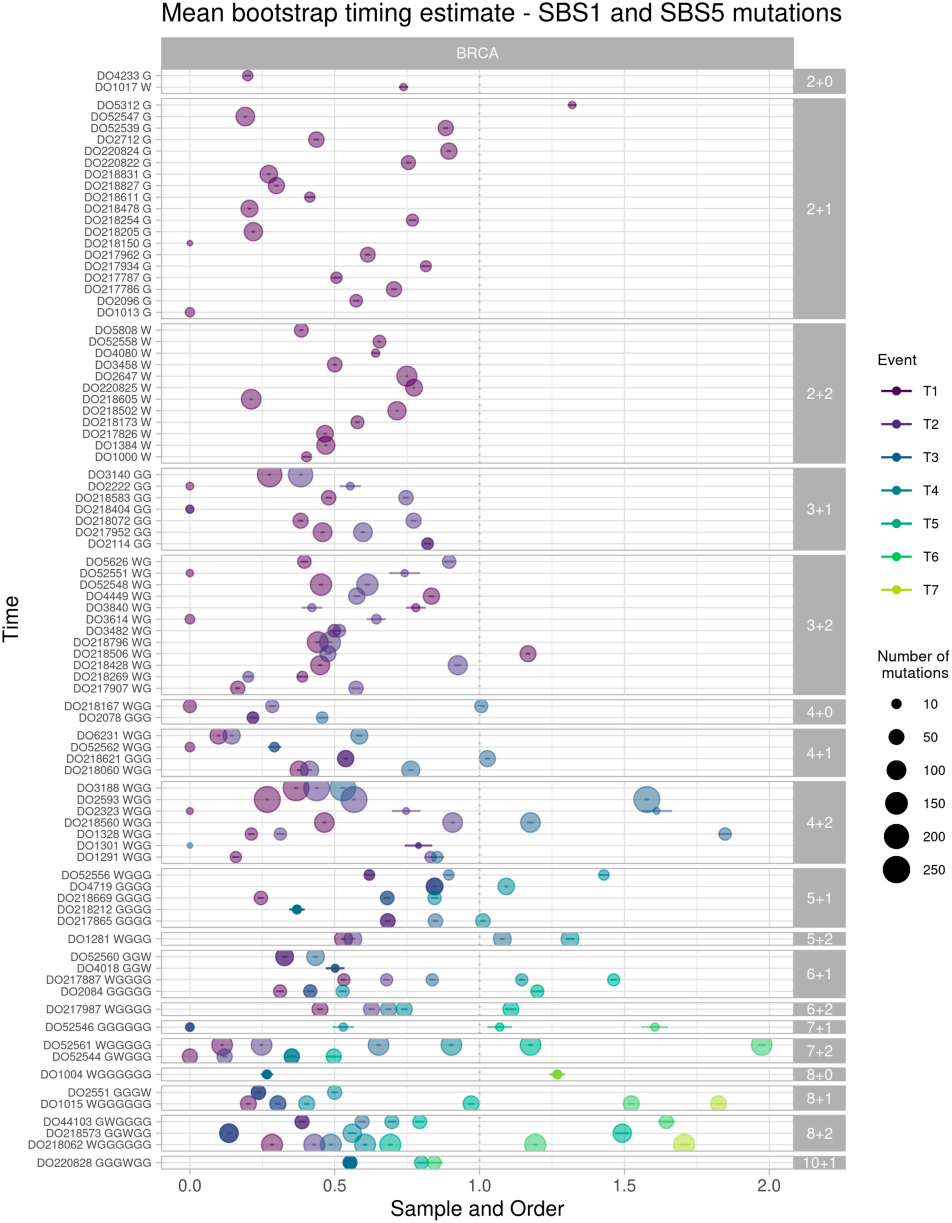
Timing estimates for MYC gains in BRCA samples. Mutations attributed to mutational signatures SBS1 and SBS5 were used to time the gain of each segment. Each row represents one sample, indicated by the patient identifier starting with “DO,” followed by the order of events inferred by AmplificationTimeR. G represents gains, and W represents whole genome duplications.

When applied to real data, AmplificationTimeR was able to time a wide range of MYC gains, both with and without whole genome duplication, identified in the PCAWG BRCA and OV samples (Fig. 5 and Supplementary Fig. S4). Interestingly, the timing of the first gain spanned a large range regardless of copy number state. However, we observed significant negative correlation between time of first MYC gain and total MYC copy number in whole genome duplicated samples, suggesting that samples with multiple gain events tend to have an earlier first gain or WGD (*P *=* *.0192, ρ = −0.327, Spearman correlation, n = 51 WGD BRCA samples timed using SBS1 and SBS5 mutations only). This trend was not observed for samples without WGD.

Although our model assumes that events should fall in the interval [0, 1], some of the timing estimates produced using real data exceed 1 (Figs 2 and 5). We postulated that this was due to a decrease in the mutation rate in samples that have been whole genome duplicated, which conflicts with the assumption of the model that mutation rate remains constant throughout the lifetime of the cancer genome, and motivated the restriction of mutations used for timing to those classified as “clock-like” (i.e. SBS1 and SBS5). This limitation affects all such models and might be addressed in future iterations of this work. However, we found that in samples that had undergone a whole genome duplication event, the timing of the first gain, which was most often due to WGD, was negatively correlated with the number of SBS1 and SBS5 mutations in both BRCA and OV samples (*P *=* *.000604, Spearman’s ρ = −0.464 and *P *=* *.00937, Spearman’s ρ = −0.396, respectively). Instead, we suggest that time points exceeding 1 simply represent events occurring late in the evolutionary history of the tumour, characterized by few to no mutations present at multiplicity 1. Indeed, replacing $n_{1}$ terms with 0 in the equations underlying AmplificationTimeR (Supplementary Text) clearly has the potential to yield timing estimates > 1. We also observe that events are occasionally called in the wrong order (i.e. $t_{1}>t_{2}$), particularly in copy number state 3 + 2. We suggest that such results represent scenarios in which gains and WGD events have been followed by a loss, in which case an incorrect timing equation has been applied as the most parsimonious (see Supplementary Text for a more detailed discussion). It is also possible that these occurrences represent situations in which AmplificationTimeR has identified an incorrect order of events from the available data, and thus applied the wrong timing equation. This may simply also be a result of having too few mutations for accurate timing. To help users identify timing results that may require further investigation we have added a set of flags to the output.

### 3.3 Choice of mutation type

#### 3.3.1 Assessment of real data

To study the effects of choice of mutation type further, we compared timing estimates produced by the three mutation types studied (i.e. all mutations in the region, mutations attributed to SBS1 and SBS5, and C > T mutations at CpG sites).

**Figure 6.**
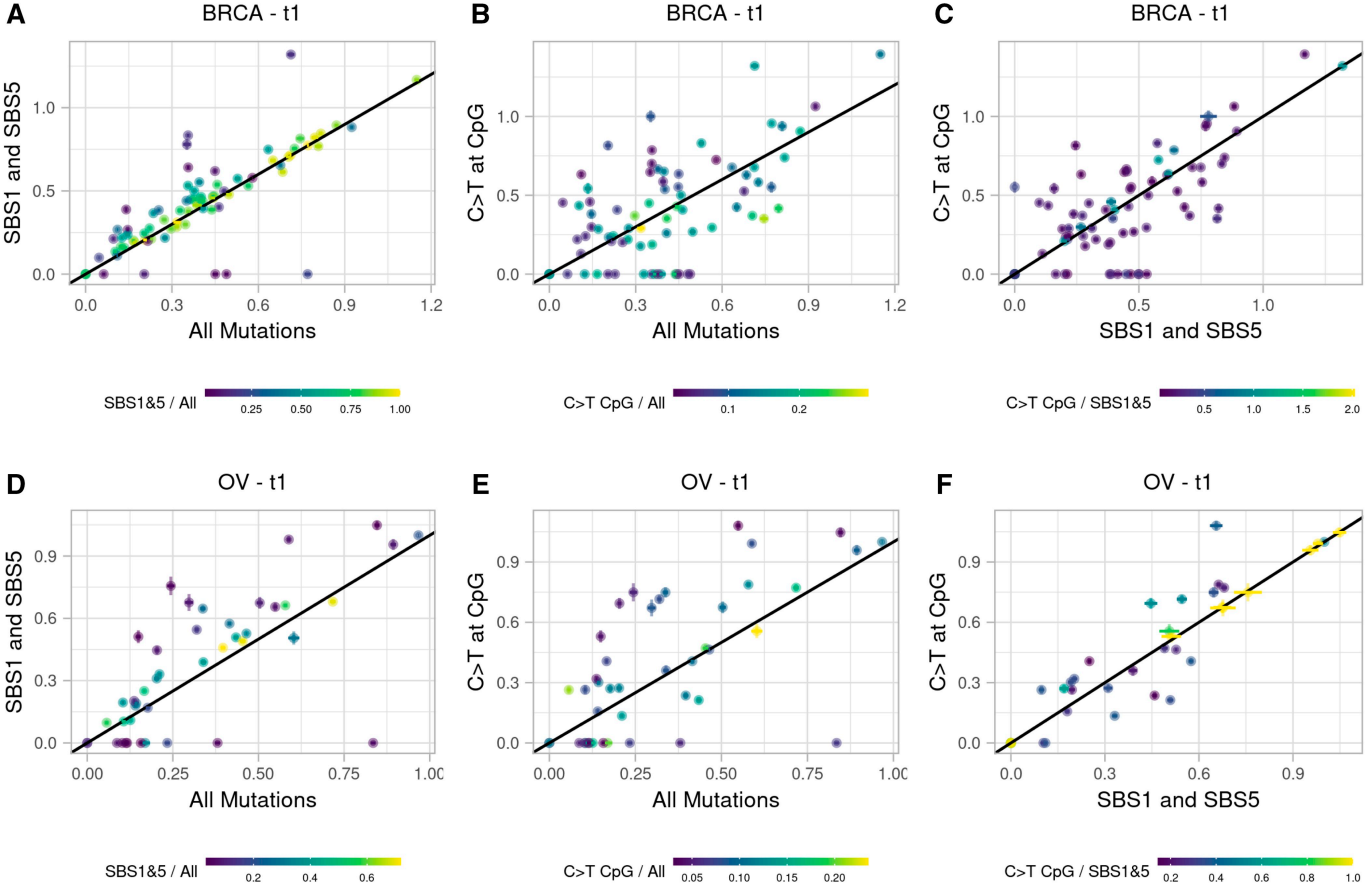
Comparison of timing estimates obtained using different mutation types for initial MYC gains or WGDs in BRCA (A–C) and OV (D–F) samples. The colour scheme indicates the ratio of the number of mutations used for each timing estimate (*y*/*x*). The mean difference in timing estimates and the results of paired Wilcoxon Rank Sum tests are listed in Supplementary Table S5 for each comparison.

Overall, we observed only minor differences in the timing estimates generated using each mutation type (Fig. 6, Supplementary Table S5). For both BRCA and OV, using all mutations to calculate timing tended to underestimate timing of the first gain relative to the estimates produced using SBS1 and SBS5 mutations or C > T mutations at CpG sites. This is expected, as many non-clock-like signatures tend to increase in activity as tumours progress (Gerstung *et al.* 2020). However, this trend was only significant for BRCA samples when comparing estimates produced using All mutations compared to SBS1 and SBS5 mutations only (*P *=* *.00178, Paired Wilcoxon Rank Sum Test with Benjamini–Hochberg adjustment for multiple testing). Interestingly, when comparing estimates obtained using C > T mutations at CpG sites to those from SBS1 and SBS5 mutations, C > T estimates of $t_{1}$ were lower than those from SBS mutations in BRCA samples but higher in OV samples. However, these differences were not significant.

#### 3.3.2 Assessment of simulated data

To address the effect of using clock-like and non-clock-like mutations more systematically we once again turn to simulated data. We randomly generated a set of time points and corresponding multiplicity values featuring varying ratios of clock-like to non-clock-like mutations for each copy number state and event order (see Supplementary Text for detailed methods). From this we assessed the average error rate of the timing estimates that were calculated compared to those that were simulated (Fig. 3B), as well as assessing AmplificationTimeR’s ability to identify the correct sequence of events (Supplementary Fig. S2B). Unsurprisingly, we found that the error rate increased with increasing proportions of non-clock-like mutations used. This provides a strong argument for restricting the mutations used to time segments to those that exhibit clock-like properties, i.e. SBS1 and SBS5 mutations, or C > T mutations occurring at CpG sites.

In contrast, AmplificationTimeR was able to identify the correct sequence of events more often when the proportion of non-clock-like mutations was higher. This is in part due to the way the simulated data were constructed, in that non-clock-like mutations were randomly selected from the pool of multiplicity states expected to be present in each scenario, whereas the multiplicities of clock-like mutations were dependent on the mutation rate and the amount of time between events. In real data, it is entirely possible that not all expected multiplicity states will be present despite including all mutations, as gain events occurring in quick succession may not allow time for mutations to occur between them, regardless of the mutational process. However, due to the information that non-clock-like mutations provide with regard to event order, when AmplificationTimeR is run on real data using only clock-like mutations, it first uses all available mutations to infer the order of events, and then restricts the mutations used for timing to clock-like mutations only. This increases the likelihood that AmplificationTimeR will identify the correct order of events, and thus use the most appropriate timing equation, while still allowing the timing itself to be carried out using only clock-like mutations.

### 3.4 Consistency across segments

As a final measure of AmplificationTimeR’s performance we investigated the consistency of WGD timing estimates calculated across different amplified segments spanning 8q from the same sample. WGD is assumed to occur only once, with concurrent gain of both chromosome copies. Thus, the timing of WGD events would be expected to be similar across segments of the same chromosome arm from the same individual. We timed all available gains on 8q for PCAWG BRCA and OV samples, and calculated the coefficient of variation for the WGD timing estimate within each sample. This was compared to a coefficient of variation calculated from WGD timing estimates that were randomly sampled from the population of 8q gains from all samples (further details in Supplementary Text). We found that the coefficient of variation for WGD events was significantly lower when calculated within individual samples compared to randomly sampled WGD events, suggesting consistency in timing (Supplementary Fig. S5; Wilcoxon rank sum test, *P *<* *.05).

## 4 Discussion

In this work, we introduce AmplificationTimeR, an R package for timing individual gains in regions of increased copy number. Timing results produced by AmplificationTimeR show good agreement with those produced by existing tools. However, AmplificationTimeR is able to time much higher copy number states than existing methods, opening the door for timing of high level amplification events in cancer. These observations could potentially be combined with analysis of mutational processes in the future to understand how these relate to each other and to disease.

Purdom *et al.* (2013) rightly point out that an exact order of events cannot be determined in scenarios where the amplified chromosome differs in different rounds of amplification, opting to exclude such segments from timing analyses. To address this, we have chosen to time segments based on the most parsimonious order of events, allowing users to time a wider range of samples. Our results suggest that such an approach is not unreasonable, as timing estimates produced by MutationTimeR, which does not exclude such segments, correlated well with our results. However, MutationTimeR only provides timing estimates for the first two gains in a series, limiting the extent of comparisons. The results of our simulations also suggest that timing estimates produced using different equations are quite consistent, in particular for earlier events.

Our experiments with simulated data also highlighted the importance of mutation number for providing accurate timing estimates, with higher numbers of mutations resulting in a lower error rate. The minimum number of mutations for which AmplificationTimeR will time a segment is 3, compared to a modifiable default of 10 for cancerTiming, and no documented minimum for MutationTimeR. However, Gerstung *et al.* (2020), in which MutationTimeR is described, used a minimum of two mutations for segment timing when using cancerTiming, so it is not unreasonable to assume that the same threshold was applied to analyses using MutationTimeR. Given the results of our simulations, we urge caution when interpreting timing estimates and event orders based on few mutations, in particular in the context of higher copy number states featuring multiple events.

The three methods compared in this paper all rely on the assumption that the mutation rate remains constant. However, different mutational processes may be active at different times during tumour development, or may occur in bursts of activity, highlighting the importance of restricting the mutations used for timing amplifications to those that occur at a constant rate (Petljak *et al.* 2019, Rubanova *et al.* 2020). For this reason, we introduced the options either to supply a list of clock-like mutations or to identify C > T mutations at CpG sites as a proxy for clocklike mutations. We found that using all mutations in a segment to time gains tended to result in an underestimation of the first gain time point relative to estimates using clock-like mutations. We also observed that the direction of the difference in timing estimates between SBS1 and SBS5 mutations and C > T mutations at CpG sites differed for BRCA and OV samples. BRCA segment times tended to be underestimated by C > T mutations, whereas OV segment times were overestimated. The results of our simulations also clearly showed the negative effects of using non-clock-like mutations on the accuracy of timing.

We recommend the use of mutational signature assignments when selecting clock-like mutations, rather than the use of C > T mutations, as this is a more nuanced identification of mutations arising from specific clock-like processes. That said, it is possible that the signatures 1 and 5 may not always behave in a clock-like manner, and that different combinations of SBS1 and SBS5 may be appropriate for different tumour types. Alexandrov *et al.* (2015) have identified tumour type specific differences in the clock-like nature of signatures 1 and 5. Further, Rubanova *et al.* (2020) observed changes in the activity of these signatures over time, suggesting that their mutation rate may not be entirely constant. This potential variation in the clock-like nature and consistency of these mutational signatures may in part underlie some of the unwanted behaviours that we observe with AmplificationTimeR, such as timing estimates >1.

The authors of both cancerTiming and MutationTimeR have remarked that confidence intervals produced by the tools are very small. We observe this trend as well and suggest that future developments in timing models might include a more sophisticated estimate of confidence, incorporating information about number of mutations, as well as the uncertainty in estimating multiplicity. The authors of MutationTimeR provide an equation for adjusting confidence intervals to make them larger; however, we found that this was unsuitable for the outputs of our model, as the upper confidence interval is constrained so that it does not exceed a time of t=1 (Gerstung *et al.* 2020). This is inappropriate for the results of our tool, as time estimates that exceed 1 will fall outside of the adjusted confidence intervals.

Currently, no method for timing amplifications takes information from adjacent genomic regions into account when calculating the timing of the amplification. It is possible for adjacent segments in a genomic region to share some but not all of their amplification history, e.g. one may observe consecutive genomic segments of copy number 2 + 1, 3 + 1, 4 + 1, and 2 + 1. The three methods compared in this paper would time each segment individually and work out the most parsimonious timing for each segment. However, it is perhaps more likely that the amplifications within the region have a shared history in which the whole segment was gained once, and sub-sections of it were gained a further one or two times. In this scenario, timing the entire region of interest would provide a more accurate timing estimate for each of the gains instead of timing each segment individually. Such an approach becomes even more important when the segments within the region of interest are small, or contain few mutations, which both contribute to uncertainty surrounding the timing estimate. Unfortunately, developing an automated method for determining which segments of the genome are likely to share an amplification history is beyond the scope of this work, so we leave it to the end user’s discretion to decide when to combine information from adjacent segments. Such an approach could also be used to improve estimation of the time of whole genome duplication events, as these affect the entire genome synchronously. We show that the current implementation of AmplificationTimeR is able to time WGD events occurring on the same chromosome arm with some consistency. However, implementing shared amplification histories, and calculating WGD time using more information would further improve the performance of AmplificationTimeR and would be an interesting direction for future development.

## 5 Conclusion

AmplificationTimeR is an R package for timing high level amplifications in cancer. Our tool incorporates information about whole genome duplication events and is able to time all events up to a copy number state of 10 + 2, with added support to restrict timing to clock-like mutations.

## Supplementary Material

btae281_Supplementary_Data

## Data Availability

AmplificationTimer is freely available as an R package hosted at https://github.com/Wedge-lab/AmplificationTimeR.
