# Supplementary material for: AmplificationTimeR: an R package for timing sequential amplification events
Source: Bioinformatics. 2024 Apr 24;40(6):btae281. doi: 10.1093/bioinformatics/btae281 (PMC11153944; doi:10.1093/bioinformatics/btae281)
Supplement: btae281_Supplementary_Data [file btae281_supplementary_data.zip › AmplificationTimeR_manuscript__Revisions_2_supplementary_tables_figures.pdf]

AmplificationTimeR Supplementary Tables and Figures

Maria Jakobsdottir

Contents

1 Supplementary Tables **2**

2 Supplementary Figures **4**

1 Supplementary Tables

Table S1: Spearman correlation between AmplificationTimeR estimate of t_1 (first MYC gain) for BRCA samples compared to MutationTimeR and cancerTiming.

	Comparison	Mutation type	ρ	p-value	Adjusted p-value
1	AmplificationTimeR v cancerTiming	All mutations	0.87	3.6e-13	4.3e-13
2	AmplificationTimeR v cancerTiming	C>T at CpG	0.82	3.5e-07	3.5e-07
3	AmplificationTimeR v cancerTiming	SBS1 and SBS5	0.94	1.8e-14	2.7e-14
4	AmplificationTimeR v MutationTimeR	All mutations	0.94	5e-34	1.9e-33
5	AmplificationTimeR v MutationTimeR	C>T at CpG	0.97	6.2e-34	1.9e-33
6	AmplificationTimeR v MutationTimeR	SBS1 and SBS5	0.96	2.9e-33	5.7e-33

Table S2: Spearman correlation between AmplificationTimeR estimate of t_2 (second MYC gain) for BRCA samples compared to MutationTimeR and cancerTiming.

	Comparison	Mutation type	ρ	p-value	Adjusted p-value
1	AmplificationTimeR v cancerTiming	All mutations	0.91	0	0
2	AmplificationTimeR v cancerTiming	C>T at CpG	0.57	0.15	0.15
3	AmplificationTimeR v cancerTiming	SBS1 and SBS5	0.86	0.011	0.013
4	AmplificationTimeR v MutationTimeR	All mutations	0.75	2.7e-09	8.1e-09
5	AmplificationTimeR v MutationTimeR	C>T at CpG	0.58	0.00052	0.00078
6	AmplificationTimeR v MutationTimeR	SBS1 and SBS5	0.7	2.1e-06	4.3e-06

Table S3: Spearman correlation between AmplificationTimeR estimate of t_1 (first MYC gain) for OV samples compared to MutationTimeR and cancerTiming.

	Comparison	Mutation type	ρ	p-value	Adjusted p-value
1	AmplificationTimeR v cancerTiming	All mutations	0.96	9.5e-16	1.9e-15
2	AmplificationTimeR v cancerTiming	C>T at CpG	0.9	5.8e-06	5.8e-06
3	AmplificationTimeR v cancerTiming	SBS1 and SBS5	0.91	5.5e-08	6.6e-08
4	AmplificationTimeR v MutationTimeR	All mutations	0.96	5.5e-30	3.3e-29
5	AmplificationTimeR v MutationTimeR	C>T at CpG	0.95	3.3e-15	5e-15
6	AmplificationTimeR v MutationTimeR	SBS1 and SBS5	0.96	9.3e-21	2.8e-20

Table S4: Spearman correlation between AmplificationTimeR estimate of t_2 (second MYC gain) for OV samples compared to MutationTimeR and cancerTiming.

	Comparison	Mutation type	ρ	p-value	Adjusted p-value
1	AmplificationTimeR v cancerTiming	All mutations	0.96	0	0
2	AmplificationTimeR v cancerTiming	C>T at CpG	0.93	0.0027	0.0027
3	AmplificationTimeR v cancerTiming	SBS1 and SBS5	0.95	0.00027	0.00033
4	AmplificationTimeR v MutationTimeR	All mutations	0.8	1e-09	2e-09
5	AmplificationTimeR v MutationTimeR	C>T at CpG	0.86	5.7e-07	8.5e-07
6	AmplificationTimeR v MutationTimeR	SBS1 and SBS5	0.94	6.7e-12	2e-11

Table S5: Mean difference in timing estimates for the first MYC gain or WGD (t_1) in BRCA and OV samples calculated using different mutation types. Paired sample Wilcoxon Rank Sum Test with Benjamini-Hochberg correction for multiple testing within tumour type.

Calculation	BRCA		OV	
	Mean Difference	Adjusted p-value	Mean Difference	Adjusted p-value
mean($t_{1All} - t_{1SBS}$)	-0.0305	0.00178	-0.0411	0.180
mean($t_{1All} - t_{1C>T}$)	-0.00494	0.998	-0.0571	0.180
mean($t_{1C>T} - t_{1SBS}$)	-0.0255	0.746	0.0160	0.451

2 Supplementary Figures

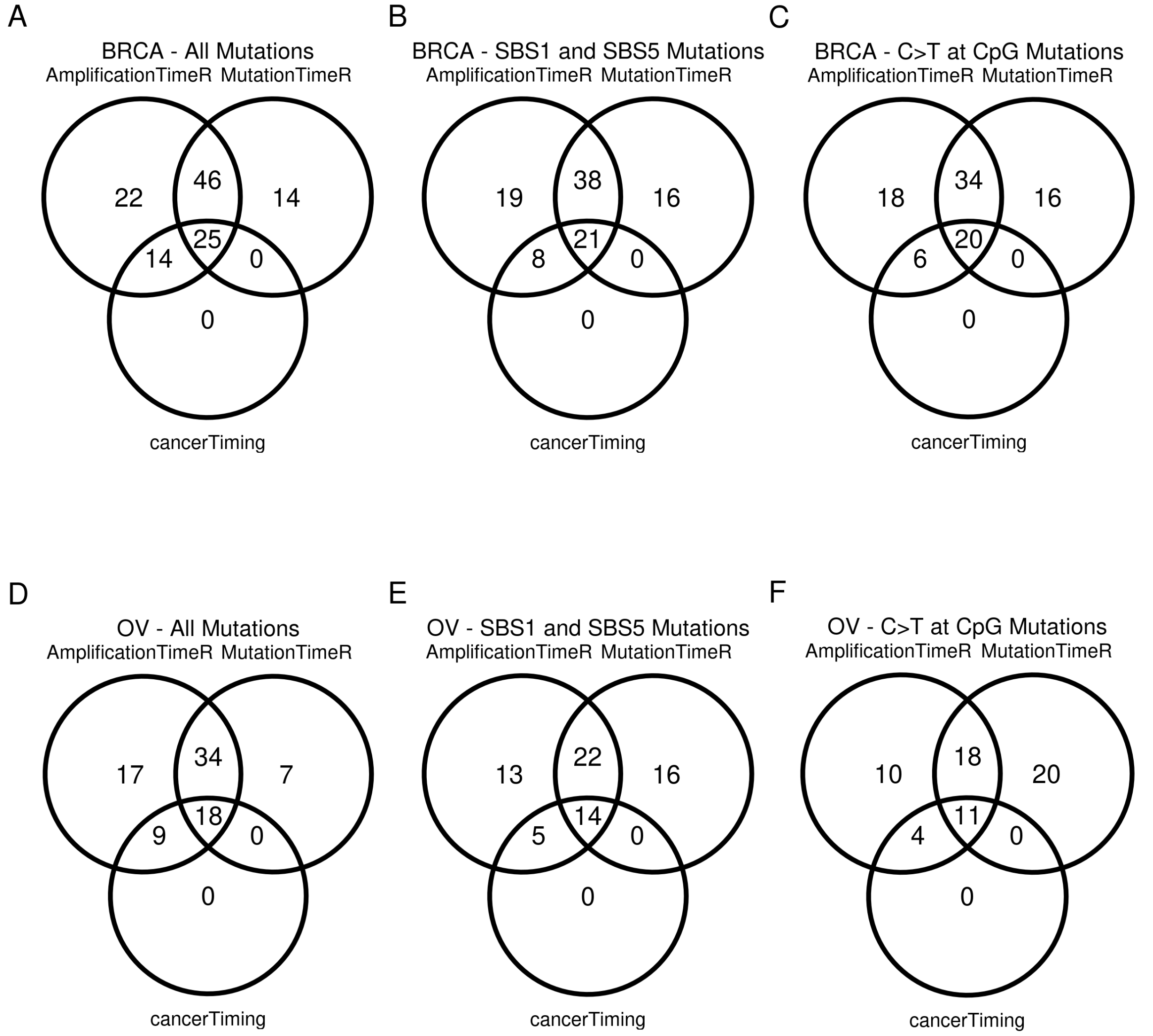

Figure S1: Venn diagrams indicating the number of samples in which MYC gains were successfully timed by each tool in the BRCA set (A-C) and OV (D-F). Timing was carried out using all mutations in the tumour (A and D), only mutations attributed to SBS1 and SBS5 (B and E), and C>T mutations at CpG islands (C and F).

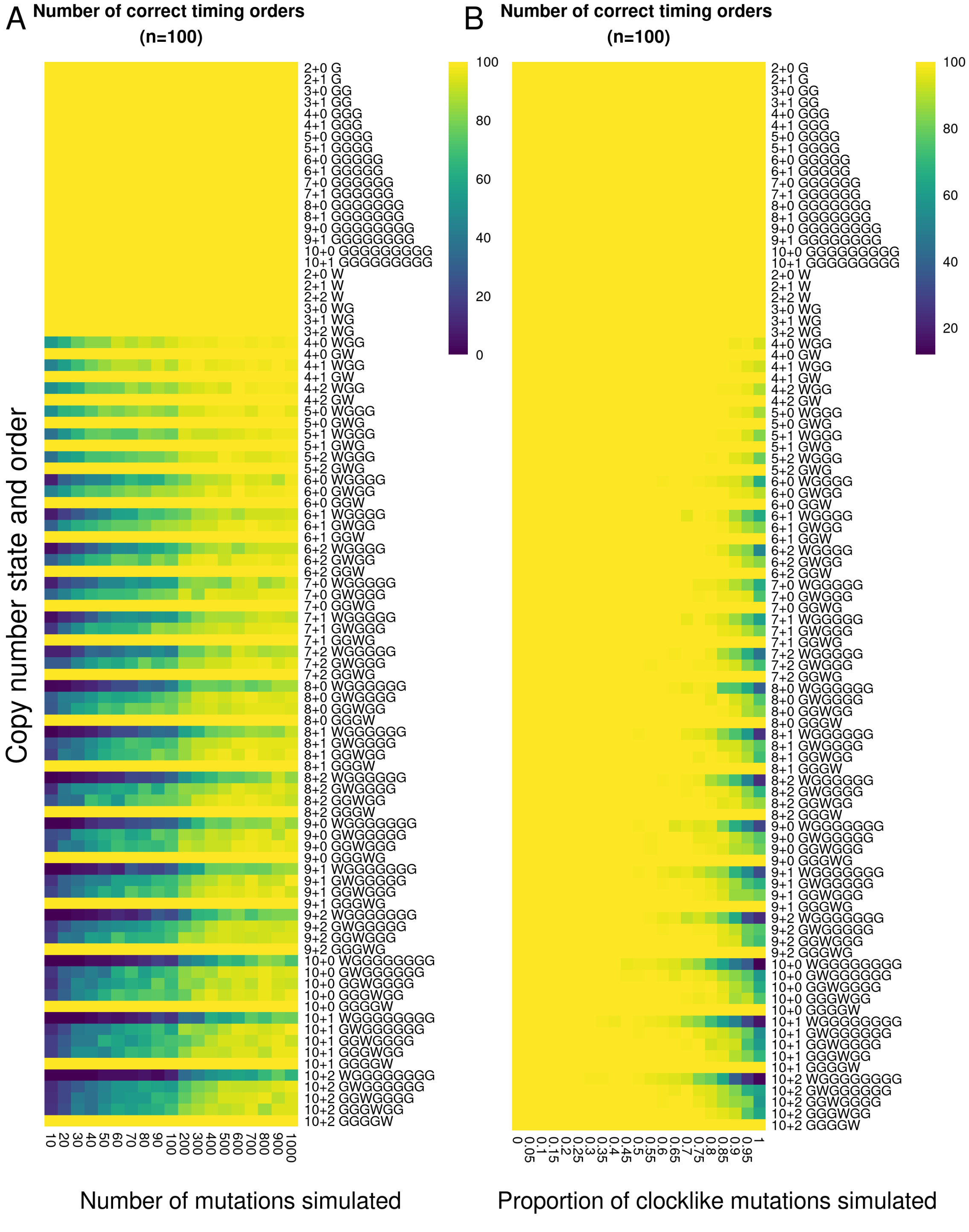

Figure S2: Number of correct event orders inferred from simulated data. n=100 randomly simulated sets of time points for each order, copy number state, and condition. **A.** Number of correct timing orders inferred when varying the number of simulated mutations. **B.** Number of correct timing orders inferred when varying the proportion of clock-like mutations simulated.

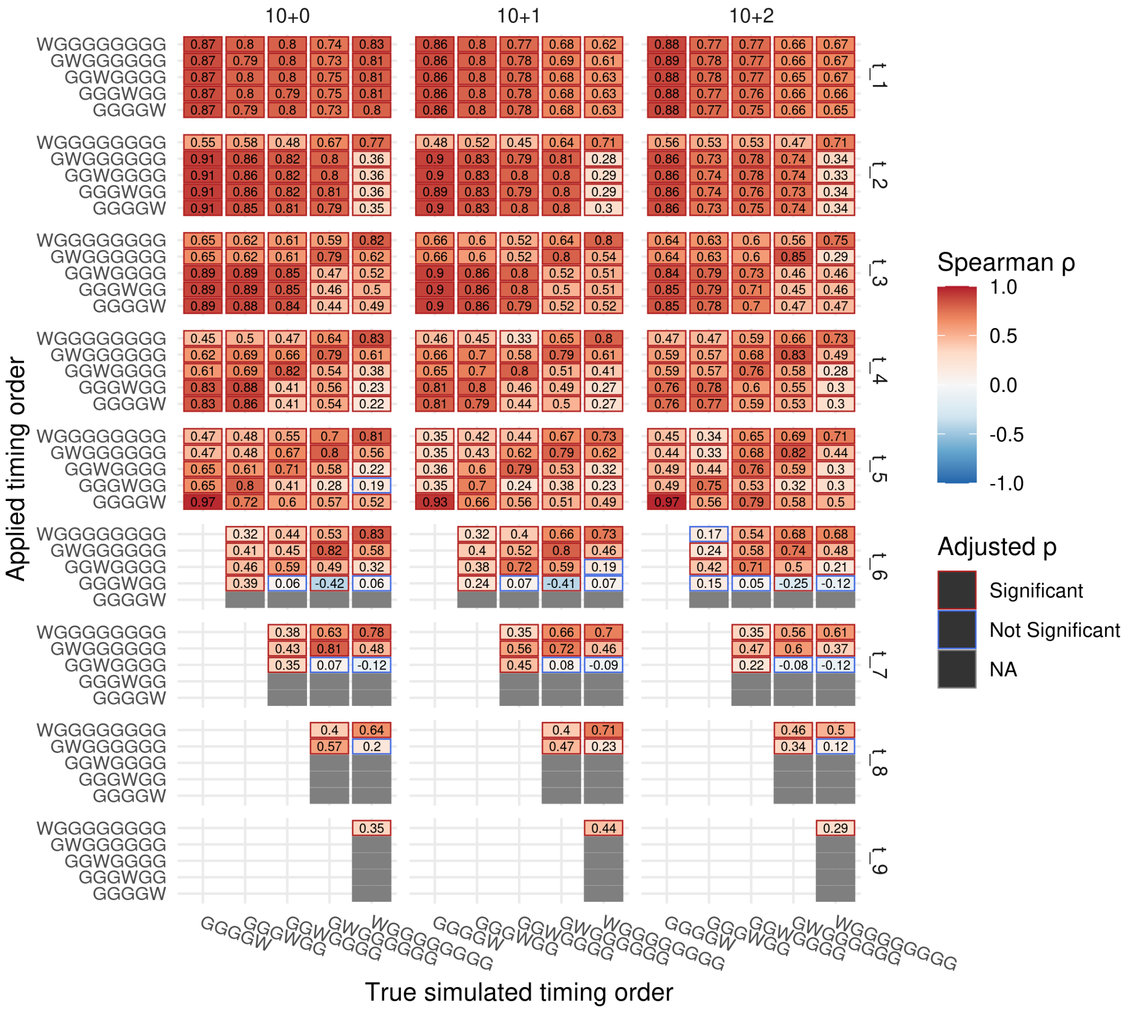

Figure S3: Spearman correlation between the simulated and calculated time for each time point when timed using all possible equations and event orders for a copy number state of 10+0, 10+1, and 10+2. The trailing diagonal represents the correlation between time points calculated using the same order of events as the data was simulated from. n = 100 randomly simulated sets of time points for each order from which a set of 100 mutations was simulated for timing. Cell colours and numbers indicate the Spearman ρ. Red cell borders indicate significant Benjamini-Hochberg adjusted p-values <0.05, whereas blue cell borders indicate adjusted p >0.05. Grey cells and cell borders indicate comparisons for which correlation could not be calculated due to differences in the number of time points calculated for different event orders.

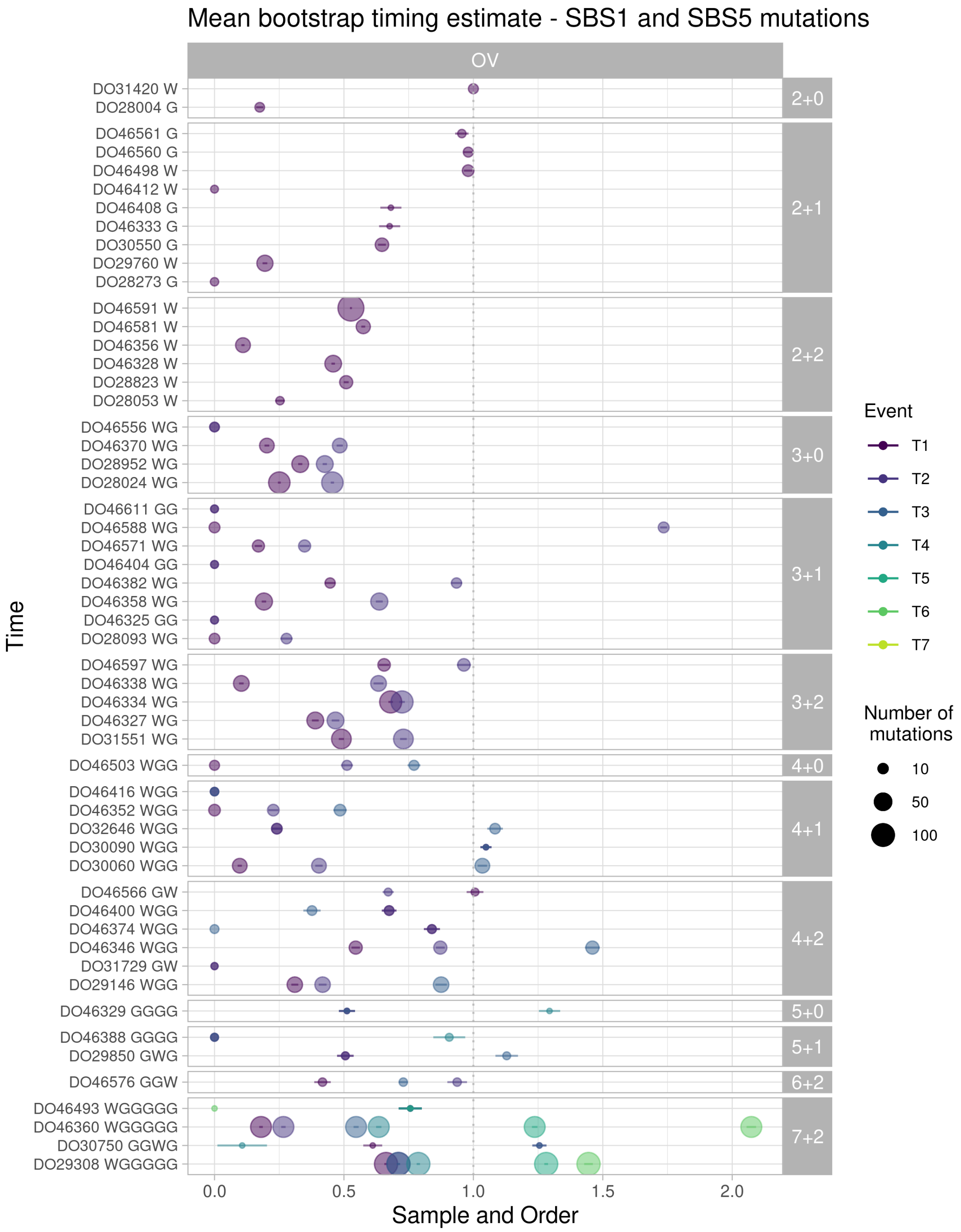

Figure S4: Timing estimates for MYC gains in OV samples. Mutations attributed to mutational signatures SBS1 and SBS5 were used to time the gain of each segment.

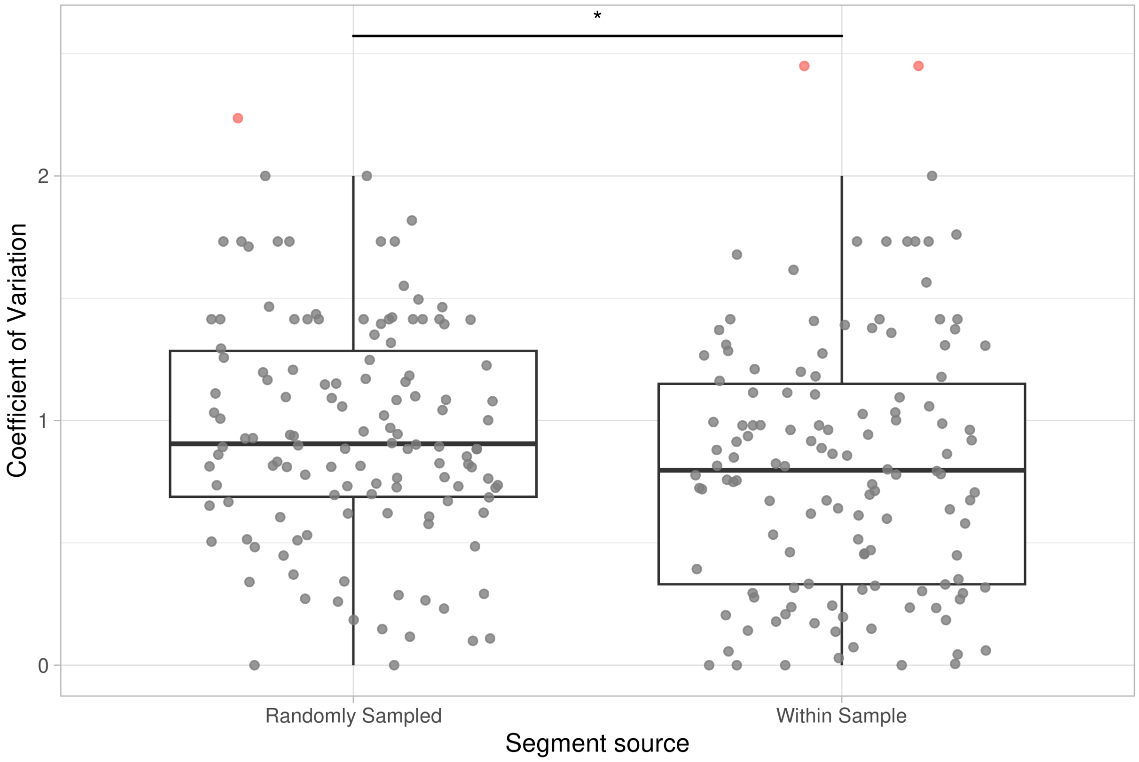

Figure S5: Coefficient of variation of the estimated WGD time calculated per sample ("Within Sample") for samples with more than one time-able segment on 8q compared to the coefficient of variation of randomly sampled 8q segments from the pool of all timed 8q segments ("Randomly Sampled"). Segments on 8q were timed using SBS1 and SBS5 mutations. (n = 124 donors and 124 randomly sampled trials) * = p <0.05 Wilcoxon rank sum test. Points indicate individual values, and red points indicate outliers. SBS1 and SBS5 mutations were used to time each segment.
