# Supplementary material for: AmplificationTimeR: an R package for timing sequential amplification events
Source: Bioinformatics. 2024 Apr 24;40(6):btae281. doi: 10.1093/bioinformatics/btae281 (PMC11153944; doi:10.1093/bioinformatics/btae281)
Supplement: btae281_Supplementary_Data [file btae281_supplementary_data.zip › AmplificationTimeR_manuscript__Revisions_2_supplementary_text.pdf]

AmplificationTimeR Supplementary Text

Maria Jakobsdottir

Contents

1 Data 3

2 Software 3

3 cancerTiming 3

4 MutationTimeR 4

5 Supplementary Methods 4
- 5.1 Simulations . . . 4
- 5.2 Simulations Varying Mutation Number . . . 4
- 5.3 Simulations Applying Different Equations . . . 4
- 5.4 Simulations Varying Clock-like Proportion . . . 4
- 5.5 Error Rate . . . 5
- 5.6 Consistency of WGD Timing Across 8q . . . 5
- 5.7 Reproducibility . . . 5

References 6

6 Full Worked Example: 4+2 WGD 7
- 6.1 General Notation and Assumptions . . . 7
- 6.2 Worked example: 4+2 . . . 7
  - 6.2.1 S_1 timing . . . 8
  - 6.2.2 S_2 timing . . . 9
  - 6.2.3 S_3 timing . . . 10

7 Equations 12
- 7.1 General Notation and Assumptions . . . 12
- 7.2 Equations - Without Whole Genome Duplication . . . 12
  - 7.2.1 2+0 Copy neutral LOH . . . 12
  - 7.2.2 2+1 Single copy gain . . . 12
  - 7.2.3 2+2 without WGD . . . 12
  - 7.2.4 3+0 . . . 12
  - 7.2.5 3+1 . . . 13
  - 7.2.6 3+2 . . . 13
  - 7.2.7 4+0 . . . 13
  - 7.2.8 4+1 . . . 13
  - 7.2.9 5+0 . . . 13
  - 7.2.10 5+1 . . . 14
  - 7.2.11 6+0 . . . 14
  - 7.2.12 6+1 . . . 14
  - 7.2.13 7+0 . . . 15
  - 7.2.14 7+1 . . . 15

7.2.15 8+0 16
7.2.16 8+1 16
7.2.17 9+0 17
7.2.18 9+1 17
7.2.19 10+0 18
7.2.20 10+1 19
7.3 Equations - With Whole Genome Duplication 20
7.3.1 Generalised solution for WGD followed by gain 20
7.3.2 2+0 21
7.3.3 2+1 21
7.3.4 2+2 21
7.3.5 3+0 22
7.3.6 3+1 22
7.3.7 3+2 23
7.3.8 4+0 24
7.3.9 4+1 25
7.3.10 4+2 27
7.3.11 5+0 28
7.3.12 5+1 29
7.3.13 5+2 30
7.3.14 6+0 31
7.3.15 6+1 33
7.3.16 6+2 35
7.3.17 7+0 37
7.3.18 7+1 39
7.3.19 7+2 41
7.3.20 8+0 43
7.3.21 8+1 45
7.3.22 8+2 47
7.3.23 9+0 49
7.3.24 9+1 52
7.3.25 9+2 55
7.3.26 10+0 57
7.3.27 10+1 60
7.3.28 10+2 63

8 Incorrect ordering of time points **66**

1 Data

`AmplificationTimeR`, `MutationTimeR` (Gerstung *et al.*, 2020), and `cancerTiming` (Purdom *et al.*, 2013) were tested on data from the PanCancer Analysis of Whole Genomes (PCAWG) project (The ICGC/TCGA Pan-Cancer Analysis of Whole Genomes Consortium, 2020). Copy number estimates derived using the Battenberg algorithm as part of work carried out by the PCAWG Evolution and Heterogeneity Working Group were provided by Stefan Dentro and used as input for all three tools (Nik-Zainal *et al.*, 2012; Gerstung *et al.*, 2020). Similarly, multiplicity estimates calculated using the `dpclust3p` `R` package (version 1.0.8) as part of the PCAWG Evolution and Heterogeneity Working Group, were also provided by Stefan Dentro, and used as input for `AmplificationTimeR` (Gerstung *et al.*, 2020). Samples whose copy number estimates did not match between the Battenberg and multiplicity files were excluded from further analysis.

Mutational signature calls were also provided by the PCAWG Mutational Signatures Working Group (Alexandrov *et al.*, 2020). Mutational signatures were assigned to individual mutations by identifying their trinucleotide context and matching this to the signature probability matrix.

2 Software

Timing using `AmplificationTimeR` and `cancerTiming` were carried out using `R` version 4.1.0 (R Core Team, 2021). Version 3.1.8 was used for `cancerTiming` (Purdom, 2016), and version 1.00.2 was used for `MutationTimeR` (Gerstung, 2020). `R` version 4.2.2 was used to run `AmplificationTimeR` version 1.1.0. All further processing, plotting, and simulation was carried out using `R` version 4.2.2 as well. The following `R` packages were used for data visualisation and processing: `ggplot2` version 3.4.4 (Wickham, 2016), `gridExtra` version 2.3 (Auguie, 2017), `data.table` version 1.14.6 (Dowle and Srinivasan, 2023), `xtable` version 1.8-4 (Dahl *et al.*, 2019), `viridis` version 0.6.4 (Garnier *et al.*, 2023), and `ggsignif` version 0.6.4 (Constantin and Patil, 2021). All experiments were run on a HPC running under CentOS Linux 7.

3 cancerTiming

`cancerTiming` (Purdom, 2016; Purdom *et al.*, 2013) was run on all histories for samples with a total copy number ≤ 5 using the ”fullMLE” method, ”nonparametric” bootstrap confidence intervals, and a minimum mutation threshold of 3 mutations. The `normCont` (normal contamination) parameter was calculated as: $\frac{2*(1-\rho)}{\psi*\rho+2*(1-\rho}$), where ρ represents purity and ψ represented ploidy, both calculated by `ASCAT` (Van Loo *et al.*, 2010). Timing was carried out for all samples with a total copy number state ≤ 5, using all mutations, C>T mutations at CpG sites, and mutations attributed to mutational signatures SBS1 and SBS5.

We found that while `cancerTiming` is able to produce timing estimates for segments in copy number states 3+0, 4+0, and 5+0, it interprets such scenarios as arising from a copy number state of 2+1, 3+1/2+2, and 4+1/3+2, respectively, based on the total copy number achieved. As this constitutes and unwanted behaviour by `cancerTiming`, and Purdom *et al.* (2013) state that they do not allow for losses on either allele, we have decided to remove timing estimates produced by `cancerTiming` for these states from our analysis. However, we have included as many of the estimates produced by `cancerTiming` as possible, such as including 2+0, 2+1, and 3+1 segments with whole genome duplications incorporated. While `cancerTiming` does not explicitly account for whole genome duplication, we decided to include these scenarios as they are timed using the same equations as their non-WGD counterparts of the same copy number by AmplificationTimeR.

4 MutationTimeR

`MutationTimeR` (Gerstung, 2020; Gerstung *et al.*, 2020) was run on copy number output produced by `Battenberg` and consensus vcf files made available through PCAWG. The `clonal_frequency` parameter was calculated as $\rho * frac1_A$ or $\rho * frac2_A$ for each segment, where ρ represents purity calculated by `Battenberg`, and $frac1_A$ and $frac2_A$ are the proportion of cells carrying the specified copy number state. Timing was carried out using all mutations, C>T mutations at CpG sites, and mutations attributed to mutational signatures SBS1 and SBS5.

5 Supplementary Methods

5.1 Simulations

For all copy number states and event orders timed by `AmplificationTimeR` we simulated a set of time points, corresponding to the number of events expected for each scenario, between 0.01 and 0.99 rounded to 2 decimal places. We then simulated the number of mutations expected at each multiplicity state by using the generated time points in combination with the multiplicity equations described in Section 7, which form the basis of the timing equations.

To make the simulated data more representative of real data we calculated the proportion of the total mutations in a segment attributed to each multiplicity state and then sampled mutations from the multinomial distribution with probability equal to the calculated proportions using the `rmultinom` function in `R`. In instances where the number of mutations of a specific multiplicity included a fraction, this was rounded down to the nearest integer. For example, in a scenario where we hoped to simulate 10 mutations, of which 3.5 had a multiplicity of 1 and 6.5 had a multiplicity of 2, we would simulate 3 mutations with multiplicity 1, and 6 mutations with multiplicity 2, resulting in a total of 9 mutations.

5.2 Simulations Varying Mutation Number

To assess the effect of mutation number on error rate and the number of correctly identified event orders we applied the approach described in Section 5.1 to simulate mutation numbers from 10 to 100 at intervals of 10, and further from 100 to 1,000 at intervals of 100. We created 100 simulations for each combination of copy number, multiplicity state, and number of mutations.

Version 1.1.0 of `AmplificationTimeR` was then run on the simulated data.

5.3 Simulations Applying Different Equations

For simulations in which all possible equations for each copy number state were run on data simulated from each of the copy number states and orders we repeated the procedure described in Section 5.1, generating 100 mutations for each scenario. We then ran a modified version of `AmplificationTimeR` in which the set of equations to be used could be specified. We then ran each set of equations on the data simulated from the relevant copy number state, and calculated the Spearman correlation between simulated and calculated time points. Each combination of simulated and calculated scenarios was tested 100 times.

5.4 Simulations Varying Clock-like Proportion

To assess the effect of the proportion of clock-like mutations on error rate and the number of correctly identified event orders we applied the approach described in Section 5.1 to simulate varying proportions of clock-like mutations at intervals of 0.05 from 0 to 1. Each simulation aimed to generate 100 mutations. Once the proportionate number of clock-like

mutations had been simulated, non-clock-like mutations were added to the set of mutations by randomly sampling, with replacement, from the multiplicity states expected for the copy number state and order of events in question. We created 100 simulations for each combination of copy number, multiplicity state, and proportion of clock-like mutations.

Version 1.1.0 of `AmplificationTimeR` was then run on the simulated data.

5.5 Error Rate

To reduce the output of the simulations described in Sections 5.2 and 5.4 down to one easily digestible number we calculated the error rate of the calculated time for each time point using Equation 1.

$$\text{Error Rate (\%)} = \frac{|t_{Calculated} - t_{Simulated}|}{t_{Simulated}} \times 100 \qquad (1)$$

We then averaged the error rate across time points for scenarios with more than one event, and subsequently averaged these values across the 100 replicates. The results were then represented as a heatmap of average error rates.

5.6 Consistency of WGD Timing Across 8q

We used version 1.1.0 of `AmplificationTimeR` to time all segments on 8q that were identified to be amplified in the PCAWG BRCA and OV samples using only SBS1 and SBS5 mutations. We then filtered the data for samples that had undergone a whole genome duplication (WGD) event and extracted the timing estimates for each WGD. Finally, we calculated the coefficient of variation between the timing estimates across different segments from the same individual for each individual.

As a comparator, we randomly selected WGD times without replacement from the pool of timed WGD segments and calculated the coefficient of variation between them. This was carried out 124 times, to match the number of real samples used, and the number of segments sampled for each random trial was matched to the distribution of the number of segments used per sample in the real data.

5.7 Reproducibility

Code for analyses and simulations run for this manuscript is available in the following GitHub repository: `https://github.com/Wedge-lab/AmplificationTimeR_paper_archive`.

References

Alexandrov, L. B., Kim, J., Haradhvala, N. J., Huang, M. N., Tian Ng, A. W., Wu, Y., Boot, A., Covington, K. R., Gordenin, D. A., Bergstrom, E. N., *et al.* (2020). The repertoire of mutational signatures in human cancer. *Nature*, **578**(7793), 94–101.

Auguie, B. (2017). *gridExtra: Miscellaneous Functions for "Grid" Graphics*. R package version 2.3.

Constantin, A.-E. and Patil, I. (2021). ggsignif: R package for displaying significance brackets for 'ggplot2'. *PsyArxiv*.

Dahl, D. B., Scott, D., Roosen, C., Magnusson, A., and Swinton, J. (2019). *xtable: Export Tables to LaTeX or HTML*. R package version 1.8-4.

Dowle, M. and Srinivasan, A. (2023). *data.table: Extension of 'data.frame'*. R package version 1.14.8.

Garnier, Simon, Ross, Noam, Rudis, Robert, Camargo, Pedro, A., Sciaini, Marco, Scherer, and Cédric (2023). *viridis(Lite) - Colorblind-Friendly Color Maps for R*. viridis package version 0.6.4.

Gerstung, M. (2020). *MutationTimeR: Timing somatic point mutations and copy number gains*. R package version 1.00.2.

Gerstung, M., Jolly, C., Leshchiner, I., Dentro, S. C., Gonzalez, S., Rosebrock, D., Mitchell, T. J., Rubanova, Y., Anur, P., Yu, K., *et al.* (2020). The evolutionary history of 2,658 cancers. *Nature*, **578**(7793), 122–128.

Nik-Zainal, S., Van Loo, P., Wedge, D. C., Alexandrov, L. B., Greenman, C. D., Lau, K. W., Raine, K., Jones, D., Marshall, J., Ramakrishna, M., *et al.* (2012). The life history of 21 breast cancers. *Cell*, **149**(5), 994–1007.

Purdom, E. (2016). *cancerTiming: Estimation of Temporal Ordering of Cancer Abnormalities*. R package version 3.1.8.

Purdom, E., Ho, C., Grasso, C. S., Quist, M. J., Cho, R. J., and Spellman, P. (2013). Methods and challenges in timing chromosomal abnormalities within cancer samples. *Bioinformatics*, **29**(24), 3113–3120.

R Core Team (2021). *R: A Language and Environment for Statistical Computing*. R Foundation for Statistical Computing, Vienna, Austria.

The ICGC/TCGA Pan-Cancer Analysis of Whole Genomes Consortium (2020). Pan-cancer analysis of whole genomes. *Nature*, **578**, 82–93.

Van Loo, P., Nordgard, S. H., Lingjærde, O. C., Russnes, H. G., Rye, I. H., Sun, W., Weigman, V. J., Marynen, P., Zetterberg, A., Naume, B., *et al.* (2010). Allele-specific copy number analysis of tumors. *Proceedings of the National Academy of Sciences*, **107**(39), 16910–16915.

Wickham, H. (2016). *ggplot2: Elegant Graphics for Data Analysis*. Springer-Verlag New York.

6 Full Worked Example: 4+2 WGD

6.1 General Notation and Assumptions

1. n_x represents the number of mutations with multiplicity x.

2. t_y represents time of event y. y can represent either a gain or a whole genome duplication event. Time begins at 0 and ends at 1, which represent the time of emergence or tumour initiation and time of tumour sampling, respectively.

3. m is used to denote the mutation rate. Mutation rate is assumed to be constant.

4. G is used to represent Gains, in scenarios where gains co-occur with whole genome duplication.

5. W is used to represent Whole genome duplication, in scenarios where gains co-occur with whole genome duplication.

6. Gr or grey is used to denote the gained chromosome, or major allele.

7. B or black is used to denote the chromosome that is not gained, or the chromosome that is gained fewer times in scenarios where both chromosomes change copy number, i.e. the minor allele.

8. Chromosome losses are not considered.

6.2 Worked example: 4+2

A copy number state of $4 + 2$ can be achieved in three different ways, termed S_1, S_2, and S_3 (See Figure ST1). Copy number $4 + 2$ was chosen for illustration purposes; the same problem applies to higher copy number states, but the number of scenarios increases.

For S_1 at time 1 (t_S, or time of sampling), chromosome A has been gained 1x, B has been gained 2x, and C and D have been gained 3x. In S_2 at time 1, chromosomes A, B, C, and D have each been gained 2x. In S_3 at time 1, chromosomes A, B, C, and D have each been gained 2x. In all scenarios, the black chromosomes have each been gained 1x. The number of times a chromosome has been gained influences the possible multiplicity states for mutations on that chromosome. A multiplicity state of 3 (n_3) can be achieved in S_1, but not S_2 or S_3. Multiplicity states n_1, n_2, and n_4 are observed in all scenarios. S_2 and S_3 cannot be differentiated from each other based on multiplicity state (or other observable features). However, the gains occurring in S_3 can be timed, whereas the gains in S_2 cannot.

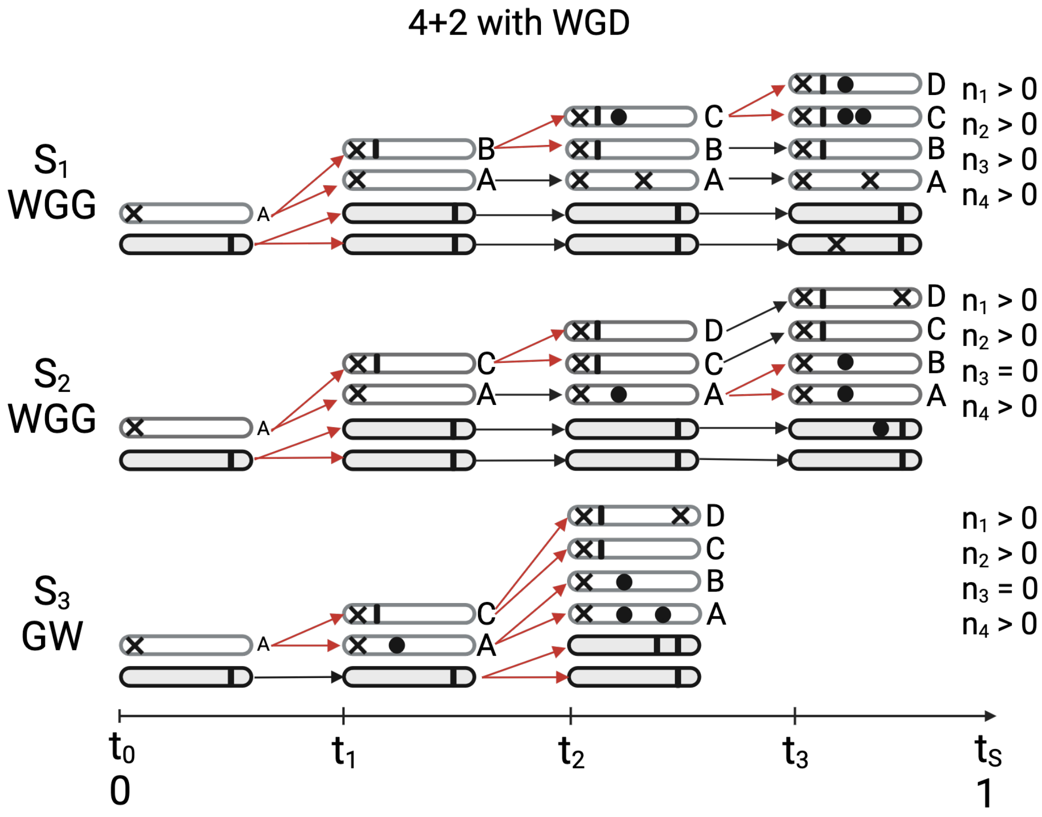

Figure ST1: Possible scenarios with copy number state 4+2 and WGD. Red arrows indicate gain events or WGD. W=WGD, G=Gain. Image created with BioRender.com

6.2.1 S_1 timing

$n_1 = m(6 - 3t_1 - t_2 - 2t_3)$
$n_2 = m(t_1 + t_3 - t_2)$
$n_3 = m(t_2 - t_1)$
$n_4 = mt_1$

$$t_1 = t_W = \frac{6n_4}{n_1 + 2n_2 + 3n_3 + 4n_4} \tag{2a}$$

$$t_2 = t_{G1} = \frac{6(n_3 + n_4)}{n_1 + 2n_2 + 3n_3 + 4n_4} \tag{2b}$$

$$t_3 = t_{G2} = \frac{6(n_2 + n_3)}{n_1 + 2n_2 + 3n_3 + 4n_4} \tag{2c}$$

6.2.1.1 Full solution

- $n_4 = mt_1$ because any mutations at multiplicity 4 will have had to happen before any gains.
- $n_3 = m(t_2 - t_1)$ because any mutations at multiplicity 3 will have had to happen after the WGD at t_1 but before the gain at t_2 on the one chromosome that is gained again at t_3 (Chromosome B in Figure ST1).
- n_2 encompasses mutations from the black chromosome which is duplicated by the WGD event (n_{2B}), and mutations from the grey chromosome that is gained at t_3

(n_{2Gr}; Chromosome C in Figure ST1).

$$n_{2B} = mt_1$$
$$n_{2Gr} = m(t_3 - t_2)$$

$$\begin{aligned} n_2 &= mt_1 + m(t_3 - t_2) \\ &= m(t_1 + t_3 - t_2) \end{aligned}$$

- n_1 encompasses mutations that occur on the black chromosomes after they are gained (n_{1B}), mutations that occur on the grey chromosome that is not gained between t_1 and t_2 (n_{Gr12}; Chromosome A), the grey chromosomes that are not gained between t_2 and t_3 (n_{Gr23}, Chromosomes A and B), and all grey chromosomes after the final gain, until the time of sampling (n_{Gr3S}).

$$\begin{aligned} n_{1B} &= 2m(1 - t_1) \\ n_{1Gr12} &= m(t_2 - t_1) \\ n_{1Gr23} &= 2m(t_3 - t_2) \\ n_{1Gr3S} &= 4m(1 - t_3) \end{aligned}$$

$$\begin{aligned} n_1 &= 2m(1 - t_1) + m(t_2 - t_1) + 2m(t_3 - t_2) + 4m(1 - t_3) \\ &= m(2 - 2t_1 + t_2 - t_1 + 2t_3 - 2t_2 + 4 - 4t_3) \\ &= m(6 - 3t_1 - t_2 - 2t_3) \end{aligned}$$

- We can then rearrange n_4 so that $m = \frac{n_4}{t_1}$, and we can plug this into equations n_1, n_2, and n_3, and solve for t_1, t_2, and t_3.

$$\begin{aligned} n_1 &= \frac{n_4}{t_1}(6 - 3t_1 - t_2 - 2t_3) \\ n_2 &= \frac{n_4}{t_1}(t_1 + t_3 - t_2) \\ n_3 &= \frac{n_4}{t_1}(t_2 - t_1) \end{aligned}$$

$$\begin{aligned} t_1 = t_W &= \frac{6n_4}{n_1 + 2n_2 + 3n_3 + 4n_4} \\ t_2 = t_{G1} &= \frac{6(n_3 + n_4)}{n_1 + 2n_2 + 3n_3 + 4n_4} \\ t_3 = t_{G2} &= \frac{6(n_2 + n_3)}{n_1 + 2n_2 + 3n_3 + 4n_4} \end{aligned}$$

6.2.2 S_2 timing

$n_1 = m(6 - 2t_1 - 2t_2 - 2t_3)$
$n_2 = m(t_2 - t_1 + t_3)$
$n_3 = 0$
$n_4 = mt_1$

The equations cannot be combined to solve for t_1, t_2, and t_3.

6.2.2.1 Full solution

- $n_4 = mt_1$ because any mutations at multiplicity 4 will have had to happen before any gains.

- $n_3 = 0$ because this multiplicity state is not observed when each chromosome is gained twice, as in S_2 (Figure ST1).

- n_2 has a contribution from the mutations on the black chromosome that occur before the WGD event (n_{2B}), mutations that occur on the first grey chromosome that is gained at t_2 after the WGD at t_1 (n_{2Gr12}; Chromosome C in Figure ST1), and mutations that occur on the second grey chromosome that is gained at t_3 after the WGD at t_1 (n_{2Gr23}; Chromosome A in Figure ST1), and the mutations that this chromosome picked up between t_1 and t_2, which are included in n_{2Gr12}.

$$\begin{aligned} n_{2B} &= mt_1 \\ n_{2Gr12} &= 2m(t_2 - t_1) \\ n_{2Gr23} &= m(t_3 - t_2) \end{aligned}$$

$$\begin{aligned} n_2 &= mt_1 + 2m(t_2 - t_1) + m(t_3 - t_2) \\ &= m(t_1 + 2t_2 - 2t_1 + t_3 - t_2) \\ &= m(t_2 - t_1 + t_3) \end{aligned}$$

- n_1 encompasses mutations that occur on the black chromosomes after they are gained (n_{1B}), on grey chromosomes C and D after they are gained but before A is gained (n_{1Gr23}) and on all grey chromosomes after the final gain until the time of sampling (n_{1Gr3S}).

$$\begin{aligned} n_{1B} &= 2m(1 - t_1) \\ n_{1Gr23} &= 2m(t_3 - t_2) \\ n_{1Gr3S} &= 4m(1 - t_3) \end{aligned}$$

$$\begin{aligned} n_1 &= 2m(1 - t_1) + 2m(t_3 - t_2) + 4m(1 - t_3) \\ &= m(2 - 2t_1 + 2t_3 - 2t_2 + 4 - 4t_3) \\ &= m(6 - 2t_1 - 2t_2 - 2t_3) \end{aligned}$$

- We can then rearrange n_4 so that $m = \frac{n_4}{t_1}$ and substitute this into equations n_1 and n_2:

$$\begin{aligned} n_1 &= \frac{n_4}{t_1}(6 - 2t_1 - 2t_2 - 2t_3) \\ n_2 &= \frac{n_4}{t_1}(t_2 - t_1 + t_3) \end{aligned}$$

- Unfortunately, two equations (n_1 and n_2) is too few to solve for three variables (t_1, t_2, and t_3).

6.2.3 S_3 timing

$n_1 = m(6 - 6t_2)$
$n_2 = m(3t_2 - 2t_1)$
$n_3 = 0$
$n_4 = mt_1$

$$t_1 = t_G = \frac{6n_4}{n_1 + 2n_2 + 4n_4} \quad (3a)$$

$$t_2 = t_W = \frac{2(n_2 + 2n_4)}{n_1 + 2n_2 + 4n_4} \quad (3b)$$

6.2.3.1 Full solution

- $n_4 = mt_1$ because any mutations present at multiplicity 4 will have had to have happened before the first event.
- $n_3 = 0$ because this multiplicity state cannot be observed when the final event is a WGD. In this case there will be not odd multiplicities besides n_1.
- n_2 consists of mutations occurring on the black chromosome before it is gained at t_2 (n_{2B}), and mutations that occur on the grey chromosomes after the first gain but before the WGD event (n_{2Gr}).

$$\begin{aligned} n_{2B} &= mt_2 \\ n_{2Gr} &= 2m(t_2 - t_1) \end{aligned}$$

$$\begin{aligned} n_2 &= mt_2 + 2m(t_2 - t_1) \\ &= m(t_2 + 2t_2 - 2t_1) \\ &= m(3t_2 - 2t_1) \end{aligned}$$

- n_1 consists of all mutations happening on any of the 6 chromosomes after the whole genome duplication event.

$$\begin{aligned} n_1 &= 6m(1 - t_2) \\ &= m(6 - 6t_2) \end{aligned}$$

- We can then rearrange n_4 so that $m = \frac{n_4}{t_1}$ and substitute this into equations n_1 and n_2, and solve for t_1 and t_2:

$$\begin{aligned} n_1 &= \frac{n_4}{t_1}(6 - 6t) \\ n_2 &= \frac{n_4}{t_1}(3t_2 - 2t_1) \end{aligned}$$

$$\begin{aligned} t_1 = t_G &= \frac{6n_4}{n_1 + 2n_2 + 4n_4} \\ t_2 = t_W &= \frac{2(n_2 + 2n_4)}{n_1 + 2n_2 + 4n_4} \end{aligned}$$

7 Equations

7.1 General Notation and Assumptions

1. n_x represents the number of mutations with multiplicity x
2. t_y represents time of event y. y can represent either a gain or a whole genome duplication event. Time begins at 0 and ends at 1, which represent the time of emergence or tumour initiation and time of tumour sampling, respectively.
3. m is used to denote the mutation rate. Mutation rate is assumed to be constant.
4. G is used to represent Gains, in scenarios where gains co-occur with whole genome duplication.
5. W is used to represent Whole genome duplication, in scenarios where gains co-occur with whole genome duplication.
6. Gr or grey is used to denote the gained chromosome, or major allele.
7. B or black is used to denote the chromosome that is not gained, or the chromosome that is gained fewer times in scenarios where both chromosomes change copy number, i.e. the minor allele.
8. Chromosome losses are not considered.

7.2 Equations - Without Whole Genome Duplication

All orders of events are treated as sequential gains.

7.2.1 2+0 Copy neutral LOH

$n_1 = 2m(1 - t_1)$
$n_2 = mt_1$

$$t_1 = \frac{2n_2}{n_1 + 2n_2} \tag{4}$$

7.2.2 2+1 Single copy gain

$n_1 = m(3 - 2t_1)$
$n_2 = mt_1$

$$t_1 = \frac{3n_2}{n_1 + 2n_2} \tag{5}$$

7.2.3 2+2 without WGD

Can't be timed.

7.2.4 3+0

$n_1 = m(3 - t_1 - 2t_2)$
$n_2 = m(t_2 - t_1)$
$n_3 = mt_1$

$$t_1 = \frac{3n_3}{n_1 + 2n_2 + 3n_3} \tag{6a}$$

$$t_2 = \frac{3(n_2 + n_3)}{n_1 + 2n_2 + 3n_3} \tag{6b}$$

7.2.5 3+1

$n_1 = m(4 - t_1 - 2t_2)$
$n_2 = m(t_2 - t_1)$
$n_3 = mt_1$

$$t_1 = \frac{4n_3}{n_1 + 2n_2 + 3n_3} \tag{7a}$$

$$t_2 = \frac{4(n_2 + n_3)}{n_1 + 2n_2 + 3n_3} \tag{7b}$$

7.2.6 3+2

Can't be timed

7.2.7 4+0

$n_1 = m(4 - t_1 - t_2 - 2t_3)$
$n_2 = m(t_3 - t_2)$
$n_3 = m(t_2 - t_1)$
$n_4 = mt_1$

$$t_1 = \frac{4n_4}{n_1 + 2n_2 + 3n_3 + 4n_4} \tag{8a}$$

$$t_2 = \frac{4(n_3 + n_4)}{n_1 + 2n_2 + 3n_3 + 4n_4} \tag{8b}$$

$$t_3 = \frac{4(n_2 + n_3 + n_4)}{n_1 + 2n_2 + 3n_3 + 4n_4} \tag{8c}$$

7.2.8 4+1

$n_1 = m(5 - t_2 - t_1 - 2t_3)$
$n_2 = m(t_3 - t_2)$
$n_3 = m(t_2 - t_1)$
$n_4 = mt_1$

$$t_1 = \frac{5n_4}{n_1 + 2n_2 + 3n_3 + 4n_4} \tag{9a}$$

$$t_2 = \frac{5(n_3 + n_4)}{n_1 + 2n_2 + 3n_3 + 4n_4} \tag{9b}$$

$$t_3 = \frac{5(n_2 + n_3 + n_4)}{n_1 + 2n_2 + 3n_3 + 4n_4} \tag{9c}$$

7.2.9 5+0

$n_1 = m(5 - t_1 - t_2 - t_3 - 2t_4)$
$n_2 = m(t_4 - t_3)$
$n_3 = m(t_3 - t_2)$
$n_4 = m(t_2 - t_1)$
$n_5 = mt_1$

$$t_1 = \frac{5n_5}{n_1 + 2n_2 + 3n_3 + 4n_4 + 5n_5} \tag{10a}$$

$$t_2 = \frac{5(n_4 + n_5)}{n_1 + 2n_2 + 3n_3 + 4n_4 + 5n_5} \tag{10b}$$

$$t_3 = \frac{5(n_3 + n_4 + n_5)}{n_1 + 2n_2 + 3n_3 + 4n_4 + 5n_5} \tag{10c}$$

$$t_4 = \frac{5(n_2 + n_3 + n_4 + n_5)}{n_1 + 2n_2 + 3n_3 + 4n_4 + 5n_5} \tag{10d}$$

7.2.10 5+1

$n_1 = m(6 - t_1 - t_2 - t_3 - 2t_4)$
$n_2 = m(t_4 - t_3)$
$n_3 = m(t_3 - t_2)$
$n_4 = m(t_2 - t_1)$
$n_5 = mt_1$

$$t_1 = t_W = \frac{6n_5}{n_1 + 2n_2 + 3n_3 + 4n_4 + 5n_5} \tag{11a}$$

$$t_2 = t_{G1} = \frac{6(n_4 + n_5)}{n_1 + 2n_2 + 3n_3 + 4n_4 + 5n_5} \tag{11b}$$

$$t_3 = t_{G2} = \frac{6(n_3 + n_4 + n_5)}{n_1 + 2n_2 + 3n_3 + 4n_4 + 5n_5} \tag{11c}$$

$$t_4 = t_{G3} = \frac{6(n_2 + n_3 + n_4 + n_5)}{n_1 + 2n_2 + 3n_3 + 4n_4 + 5n_5} \tag{11d}$$

7.2.11 6+0

$n_1 = m(6 - t_1 - t_2 - t_3 - t_4 - 2t_5)$
$n_2 = m(t_5 - t_4)$
$n_3 = m(t_4 - t_3)$
$n_4 = m(t_3 - t_2)$
$n_5 = m(t_2 - t_1)$
$n_6 = mt_1$

$$t_1 = \frac{6n_6}{n_1 + 2n_2 + 3n_3 + 4n_4 + 5n_5 + 6n_6} \tag{12a}$$

$$t_2 = \frac{6(n_5 + n_6)}{n_1 + 2n_2 + 3n_3 + 4n_4 + 5n_5 + 6n_6} \tag{12b}$$

$$t_3 = \frac{6(n_4 + n_5 + n_6)}{n_1 + 2n_2 + 3n_3 + 4n_4 + 5n_5 + 6n_6} \tag{12c}$$

$$t_4 = \frac{6(n_3 + n_4 + n_5 + n_6)}{n_1 + 2n_2 + 3n_3 + 4n_4 + 5n_5 + 6n_6} \tag{12d}$$

$$t_5 = \frac{6(n_2 + n_3 + n_4 + n_5 + n_6)}{n_1 + 2n_2 + 3n_3 + 4n_4 + 5n_5 + 6n_6} \tag{12e}$$

7.2.12 6+1

$n_1 = m(7 - t_1 - t_2 - t_3 - t_4 - 2t_5)$
$n_2 = m(t_5 - t_4)$
$n_3 = m(t_4 - t_3)$
$n_4 = m(t_3 - t_2)$
$n_5 = m(t_2 - t_1)$
$n_6 = mt_1$

$$t_1 = t_W = \frac{7n_6}{n_1 + 2n_2 + 3n_3 + 4n_4 + 5n_5 + 6n_6} \tag{13a}$$

$$t_2 = t_{G1} = \frac{7(n_5 + n_6)}{n_1 + 2n_2 + 3n_3 + 4n_4 + 5n_5 + 6n_6} \tag{13b}$$

$$t_3 = t_{G2} = \frac{7(n_4 + n_5 + n_6)}{n_1 + 2n_2 + 3n_3 + 4n_4 + 5n_5 + 6n_6} \tag{13c}$$

$$t_4 = t_{G3} = \frac{7(n_3 + n_4 + n_5 + n_6)}{n_1 + 2n_2 + 3n_3 + 4n_4 + 5n_5 + 6n_6} \tag{13d}$$

$$t_5 = t_{G4} = \frac{7(n_2 + n_3 + n_4 + n_5 + n_6)}{n_1 + 2n_2 + 3n_3 + 4n_4 + 5n_5 + 6n_6} \tag{13e}$$

7.2.13 7+0

$n_1 = m(7 - t_1 - t_2 - t_3 - t_4 - t_5 - 2t_6)$
$n_2 = m(t_6 - t_5)$
$n_3 = m(t_5 - t_4)$
$n_4 = m(t_4 - t_3)$
$n_5 = m(t_3 - t_2)$
$n_6 = m(t_2 - t_1)$
$n_7 = mt_1$

$$t_1 = \frac{7n_7}{n_1 + 2n_2 + 3n_3 + 4n_4 + 5n_5 + 6n_6 + 7n_7} \tag{14a}$$

$$t_2 = \frac{7(n_6 + n_7)}{n_1 + 2n_2 + 3n_3 + 4n_4 + 5n_5 + 6n_6 + 7n_7} \tag{14b}$$

$$t_3 = \frac{7(n_5 + n_6 + n_7)}{n_1 + 2n_2 + 3n_3 + 4n_4 + 5n_5 + 6n_6 + 7n_7} \tag{14c}$$

$$t_4 = \frac{7(n_4 + n_5 + n_6 + n_7)}{n_1 + 2n_2 + 3n_3 + 4n_4 + 5n_5 + 6n_6 + 7n_7} \tag{14d}$$

$$t_5 = \frac{7(n_3 + n_4 + n_5 + n_6 + n_7)}{n_1 + 2n_2 + 3n_3 + 4n_4 + 5n_5 + 6n_6 + 7n_7} \tag{14e}$$

$$t_6 = \frac{7(n_2 + n_3 + n_4 + n_5 + n_6 + n_7)}{n_1 + 2n_2 + 3n_3 + 4n_4 + 5n_5 + 6n_6 + 7n_7} \tag{14f}$$

7.2.14 7+1

$n_1 = m(8 - t_1 - t_2 - t_3 - t_4 - t_5 - 2t_6)$
$n_2 = m(t_6 - t_5)$
$n_3 = m(t_5 - t_4)$
$n_4 = m(t_4 - t_3)$
$n_5 = m(t_3 - t_2)$
$n_6 = m(t_2 - t_1)$
$n_7 = mt_1$

$$t_1 = t_W = \frac{8n_7}{n_1 + 2n_2 + 3n_3 + 4n_4 + 5n_5 + 6n_6 + 7n_7} \tag{15a}$$

$$t_2 = t_{G1} = \frac{8(n_6 + n_7)}{n_1 + 2n_2 + 3n_3 + 4n_4 + 5n_5 + 6n_6 + 7n_7} \tag{15b}$$

$$t_3 = t_{G2} = \frac{8(n_5 + n_6 + n_7)}{n_1 + 2n_2 + 3n_3 + 4n_4 + 5n_5 + 6n_6 + 7n_7} \tag{15c}$$

$$t_4 = t_{G3} = \frac{8(n_4 + n_5 + n_6 + n_7)}{n_1 + 2n_2 + 3n_3 + 4n_4 + 5n_5 + 6n_6 + 7n_7} \tag{15d}$$

$$t_5 = t_{G4} = \frac{8(n_3 + n_4 + n_5 + n_6 + n_7)}{n_1 + 2n_2 + 3n_3 + 4n_4 + 5n_5 + 6n_6 + 7n_7} \tag{15e}$$

$$t_6 = t_{G5} = \frac{8(n_2 + n_3 + n_4 + n_5 + n_6 + n_7)}{n_1 + 2n_2 + 3n_3 + 4n_4 + 5n_5 + 6n_6 + 7n_7} \tag{15f}$$

7.2.15 8+0

$n_1 = m(8 - t_1 - t_2 - t_3 - t_4 - t_5 - t_6 - 2t_7)$
$n_2 = m(t_7 - t_6)$
$n_3 = m(t_6 - t_5)$
$n_4 = m(t_5 - t_4)$
$n_5 = m(t_4 - t_3)$
$n_6 = m(t_3 - t_2)$
$n_7 = m(t_2 - t_1)$
$n_8 = mt_1$

$$t_1 = \frac{8n_8}{n_1 + 2n_2 + 3n_3 + 4n_4 + 5n_5 + 6n_6 + 7n_7 + 8n_8} \tag{16a}$$

$$t_2 = \frac{8(n_7 + n_8)}{n_1 + 2n_2 + 3n_3 + 4n_4 + 5n_5 + 6n_6 + 7n_7 + 8n_8} \tag{16b}$$

$$t_3 = \frac{8(n_6 + n_7 + n_8)}{n_1 + 2n_2 + 3n_3 + 4n_4 + 5n_5 + 6n_6 + 7n_7 + 8n_8} \tag{16c}$$

$$t_4 = \frac{8(n_5 + n_6 + n_7 + n_8)}{n_1 + 2n_2 + 3n_3 + 4n_4 + 5n_5 + 6n_6 + 7n_7 + 8n_8} \tag{16d}$$

$$t_5 = \frac{8(n_4 + n_5 + n_6 + n_7 + n_8)}{n_1 + 2n_2 + 3n_3 + 4n_4 + 5n_5 + 6n_6 + 7n_7 + 8n_8} \tag{16e}$$

$$t_6 = \frac{8(n_3 + n_4 + n_5 + n_6 + n_7 + n_8)}{n_1 + 2n_2 + 3n_3 + 4n_4 + 5n_5 + 6n_6 + 7n_7 + 8n_8} \tag{16f}$$

$$t_7 = \frac{8(n_2 + n_3 + n_4 + n_5 + n_6 + n_7 + n_8)}{n_1 + 2n_2 + 3n_3 + 4n_4 + 5n_5 + 6n_6 + 7n_7 + 8n_8} \tag{16g}$$

7.2.16 8+1

$n_1 = m(9 - t_1 - t_2 - t_3 - t_4 - t_5 - t_6 - 2t_7)$
$n_2 = m(t_7 - t_6)$
$n_3 = m(t_6 - t_5)$
$n_4 = m(t_5 - t_4)$
$n_5 = m(t_4 - t_3)$
$n_6 = m(t_3 - t_2)$
$n_7 = m(t_2 - t_1)$
$n_8 = mt_1$

$$t_1 = t_W = \frac{9n_8}{n_1 + 2n_2 + 3n_3 + 4n_4 + 5n_5 + 6n_6 + 7n_7 + 8n_8} \tag{17a}$$

$$t_2 = t_{G1} = \frac{9(n_7 + n_8)}{n_1 + 2n_2 + 3n_3 + 4n_4 + 5n_5 + 6n_6 + 7n_7 + 8n_8} \tag{17b}$$

$$t_3 = t_{G2} = \frac{9(n_6 + n_7 + n_8)}{n_1 + 2n_2 + 3n_3 + 4n_4 + 5n_5 + 6n_6 + 7n_7 + 8n_8} \tag{17c}$$

$$t_4 = t_{G3} = \frac{9(n_5 + n_6 + n_7 + n_8)}{n_1 + 2n_2 + 3n_3 + 4n_4 + 5n_5 + 6n_6 + 7n_7 + 8n_8} \tag{17d}$$

$$t_5 = t_{G4} = \frac{9(n_4 + n_5 + n_6 + n_7 + n_8)}{n_1 + 2n_2 + 3n_3 + 4n_4 + 5n_5 + 6n_6 + 7n_7 + 8n_8} \tag{17e}$$

$$t_6 = t_{G5} = \frac{9(n_3 + n_4 + n_5 + n_6 + n_7 + n_8)}{n_1 + 2n_2 + 3n_3 + 4n_4 + 5n_5 + 6n_6 + 7n_7 + 8n_8} \tag{17f}$$

$$t_7 = t_{G6} = \frac{9(n_2 + n_3 + n_4 + n_5 + n_6 + n_7 + n_8)}{n_1 + 2n_2 + 3n_3 + 4n_4 + 5n_5 + 6n_6 + 7n_7 + 8n_8} \tag{17g}$$

7.2.17 9+0

$n_1 = m(9 - t_1 - t_2 - t_3 - t_4 - t_5 - t_6 - t_7 - 2t_8)$
$n_2 = m(t_8 - t_7)$
$n_3 = m(t_7 - t_6)$
$n_4 = m(t_6 - t_5)$
$n_5 = m(t_5 - t_4)$
$n_6 = m(t_4 - t_3)$
$n_7 = m(t_3 - t_2)$
$n_8 = m(t_2 - t_1)$
$n_9 = mt_1$

$$t_1 = \frac{9n_9}{n_1 + 2n_2 + 3n_3 + 4n_4 + 5n_5 + 6n_6 + 7n_7 + 8n_8 + 9n_9} \tag{18a}$$

$$t_2 = \frac{9(n_8 + n_9)}{n_1 + 2n_2 + 3n_3 + 4n_4 + 5n_5 + 6n_6 + 7n_7 + 8n_8 + 9n_9} \tag{18b}$$

$$t_3 = \frac{9(n_7 + n_8 + n_9)}{n_1 + 2n_2 + 3n_3 + 4n_4 + 5n_5 + 6n_6 + 7n_7 + 8n_8 + 9n_9} \tag{18c}$$

$$t_4 = \frac{9(n_6 + n_7 + n_8 + n_9)}{n_1 + 2n_2 + 3n_3 + 4n_4 + 5n_5 + 6n_6 + 7n_7 + 8n_8 + 9n_9} \tag{18d}$$

$$t_5 = \frac{9(n_5 + n_6 + n_7 + n_8 + n_9)}{n_1 + 2n_2 + 3n_3 + 4n_4 + 5n_5 + 6n_6 + 7n_7 + 8n_8 + 9n_9} \tag{18e}$$

$$t_6 = \frac{9(n_4 + n_5 + n_6 + n_7 + n_8 + n_9)}{n_1 + 2n_2 + 3n_3 + 4n_4 + 5n_5 + 6n_6 + 7n_7 + 8n_8 + 9n_9} \tag{18f}$$

$$t_7 = \frac{9(n_3 + n_4 + n_5 + n_6 + n_7 + n_8 + n_9)}{n_1 + 2n_2 + 3n_3 + 4n_4 + 5n_5 + 6n_6 + 7n_7 + 8n_8 + 9n_9} \tag{18g}$$

$$t_8 = \frac{9(n_2 + n_3 + n_4 + n_5 + n_6 + n_7 + n_8 + n_9)}{n_1 + 2n_2 + 3n_3 + 4n_4 + 5n_5 + 6n_6 + 7n_7 + 8n_8 + 9n_9} \tag{18h}$$

7.2.18 9+1

$n_1 = m(10 - t_1 - t_2 - t_3 - t_4 - t_5 - t_6 - t_7 - 2t_8)$
$n_2 = m(t_8 - t_7)$
$n_3 = m(t_7 - t_6)$
$n_4 = m(t_6 - t_5)$
$n_5 = m(t_5 - t_4)$
$n_6 = m(t_4 - t_3)$
$n_7 = m(t_3 - t_2)$
$n_8 = m(t_2 - t_1)$
$n_9 = mt_1$

$$t_1 = t_W = \frac{10n_9}{n_1 + 2n_2 + 3n_3 + 4n_4 + 5n_5 + 6n_6 + 7n_7 + 8n_8 + 9n_9} \tag{19a}$$

$$t_2 = t_{G1} = \frac{10(n_8 + n_9)}{n_1 + 2n_2 + 3n_3 + 4n_4 + 5n_5 + 6n_6 + 7n_7 + 8n_8 + 9n_9} \tag{19b}$$

$$t_3 = t_{G2} = \frac{10(n_7 + n_8 + n_9)}{n_1 + 2n_2 + 3n_3 + 4n_4 + 5n_5 + 6n_6 + 7n_7 + 8n_8 + 9n_9} \tag{19c}$$

$$t_4 = t_{G3} = \frac{10(n_6 + n_7 + n_8 + n_9)}{n_1 + 2n_2 + 3n_3 + 4n_4 + 5n_5 + 6n_6 + 7n_7 + 8n_8 + 9n_9} \tag{19d}$$

$$t_5 = t_{G4} = \frac{10(n_5 + n_6 + n_7 + n_8 + n_9)}{n_1 + 2n_2 + 3n_3 + 4n_4 + 5n_5 + 6n_6 + 7n_7 + 8n_8 + 9n_9} \tag{19e}$$

$$t_6 = t_{G5} = \frac{10(n_4 + n_5 + n_6 + n_7 + n_8 + n_9)}{n_1 + 2n_2 + 3n_3 + 4n_4 + 5n_5 + 6n_6 + 7n_7 + 8n_8 + 9n_9} \tag{19f}$$

$$t_7 = t_{G6} = \frac{10(n_3 + n_4 + n_5 + n_6 + n_7 + n_8 + n_9)}{n_1 + 2n_2 + 3n_3 + 4n_4 + 5n_5 + 6n_6 + 7n_7 + 8n_8 + 9n_9} \tag{19g}$$

$$t_8 = t_{G7} = \frac{10(n_2 + n_3 + n_4 + n_5 + n_6 + n_7 + n_8 + n_9)}{n_1 + 2n_2 + 3n_3 + 4n_4 + 5n_5 + 6n_6 + 7n_7 + 8n_8 + 9n_9} \tag{19h}$$

7.2.19 10+0

$n_1 = m(10 - t_1 - t_2 - t_3 - t_4 - t_5 - t_6 - t_7 - t_8 - 2t_9)$
$n_2 = m(t_9 - t_8)$
$n_3 = m(t_8 - t_7)$
$n_4 = m(t_7 - t_6)$
$n_5 = m(t_6 - t_5)$
$n_6 = m(t_5 - t_4)$
$n_7 = m(t_4 - t_3)$
$n_8 = m(t_3 - t_2)$
$n_9 = m(t_2 - t_1)$
$n_{10} = mt_1$

$$t_1 = \frac{10n_{10}}{n_1 + 2n_2 + 3n_3 + 4n_4 + 5n_5 + 6n_6 + 7n_7 + 8n_8 + 9n_9 + 10n_{10}} \tag{20a}$$

$$t_2 = \frac{10(n_9 + n_{10})}{n_1 + 2n_2 + 3n_3 + 4n_4 + 5n_5 + 6n_6 + 7n_7 + 8n_8 + 9n_9 + 10n_{10}} \tag{20b}$$

$$t_3 = \frac{10(n_8 + n_9 + n_{10})}{n_1 + 2n_2 + 3n_3 + 4n_4 + 5n_5 + 6n_6 + 7n_7 + 8n_8 + 9n_9 + 10n_{10}} \tag{20c}$$

$$t_4 = \frac{10(n_7 + n_8 + n_9 + n_{10})}{n_1 + 2n_2 + 3n_3 + 4n_4 + 5n_5 + 6n_6 + 7n_7 + 8n_8 + 9n_9 + 10n_{10}} \tag{20d}$$

$$t_5 = \frac{10(n_6 + n_7 + n_8 + n_9 + n_{10})}{n_1 + 2n_2 + 3n_3 + 4n_4 + 5n_5 + 6n_6 + 7n_7 + 8n_8 + 9n_9 + 10n_{10}} \tag{20e}$$

$$t_6 = \frac{10(n_5 + n_6 + n_7 + n_8 + n_9 + n_{10})}{n_1 + 2n_2 + 3n_3 + 4n_4 + 5n_5 + 6n_6 + 7n_7 + 8n_8 + 9n_9 + 10n_{10}} \tag{20f}$$

$$t_7 = \frac{10(n_4 + n_5 + n_6 + n_7 + n_8 + n_9 + n_{10})}{n_1 + 2n_2 + 3n_3 + 4n_4 + 5n_5 + 6n_6 + 7n_7 + 8n_8 + 9n_9 + 10n_{10}} \tag{20g}$$

$$t_8 = \frac{10(n_3 + n_4 + n_5 + n_6 + n_7 + n_8 + n_9 + n_{10})}{n_1 + 2n_2 + 3n_3 + 4n_4 + 5n_5 + 6n_6 + 7n_7 + 8n_8 + 9n_9 + 10n_{10}} \tag{20h}$$

$$t_9 = \frac{10(n_2 + n_3 + n_4 + n_5 + n_6 + n_7 + n_8 + n_9 + n_{10})}{n_1 + 2n_2 + 3n_3 + 4n_4 + 5n_5 + 6n_6 + 7n_7 + 8n_8 + 9n_9 + 10n_{10}} \tag{20i}$$

7.2.20 10+1

$n_1 = m(11 - t_1 - t_2 - t_3 - t_4 - t_5 - t_6 - t_7 - t_8 - 2t_9)$
$n_2 = m(t_9 - t_8)$
$n_3 = m(t_8 - t_7)$
$n_4 = m(t_7 - t_6)$
$n_5 = m(t_6 - t_5)$
$n_6 = m(t_5 - t_4)$
$n_7 = m(t_4 - t_3)$
$n_8 = m(t_3 - t_2)$
$n_9 = m(t_2 - t_1)$
$n_{10} = mt_1$

$$t_1 = t_W = \frac{11n_{10}}{n_1 + 2n_2 + 3n_3 + 4n_4 + 5n_5 + 6n_6 + 7n_7 + 8n_8 + 9n_9 + 10n_{10}} \tag{21a}$$

$$t_2 = t_{G1} = \frac{11(n_9 + n_{10})}{n_1 + 2n_2 + 3n_3 + 4n_4 + 5n_5 + 6n_6 + 7n_7 + 8n_8 + 9n_9 + 10n_{10}} \tag{21b}$$

$$t_3 = t_{G2} = \frac{11(n_8 + n_9 + n_{10})}{n_1 + 2n_2 + 3n_3 + 4n_4 + 5n_5 + 6n_6 + 7n_7 + 8n_8 + 9n_9 + 10n_{10}} \tag{21c}$$

$$t_4 = t_{G3} = \frac{11(n_7 + n_8 + n_9 + n_{10})}{n_1 + 2n_2 + 3n_3 + 4n_4 + 5n_5 + 6n_6 + 7n_7 + 8n_8 + 9n_9 + 10n_{10}} \tag{21d}$$

$$t_5 = t_{G4} = \frac{11(n_6 + n_7 + n_8 + n_9 + n_{10})}{n_1 + 2n_2 + 3n_3 + 4n_4 + 5n_5 + 6n_6 + 7n_7 + 8n_8 + 9n_9 + 10n_{10}} \tag{21e}$$

$$t_6 = t_{G5} = \frac{11(n_5 + n_6 + n_7 + n_8 + n_9 + n_{10})}{n_1 + 2n_2 + 3n_3 + 4n_4 + 5n_5 + 6n_6 + 7n_7 + 8n_8 + 9n_9 + 10n_{10}} \tag{21f}$$

$$t_7 = t_{G6} = \frac{11(n_4 + n_5 + n_6 + n_7 + n_8 + n_9 + n_{10})}{n_1 + 2n_2 + 3n_3 + 4n_4 + 5n_5 + 6n_6 + 7n_7 + 8n_8 + 9n_9 + 10n_{10}} \tag{21g}$$

$$t_8 = t_{G7} = \frac{11(n_3 + n_4 + n_5 + n_6 + n_7 + n_8 + n_9 + n_{10})}{n_1 + 2n_2 + 3n_3 + 4n_4 + 5n_5 + 6n_6 + 7n_7 + 8n_8 + 9n_9 + 10n_{10}} \tag{21h}$$

$$t_9 = t_{G8} = \frac{11(n_2 + n_3 + n_4 + n_5 + n_6 + n_7 + n_8 + n_9 + n_{10})}{n_1 + 2n_2 + 3n_3 + 4n_4 + 5n_5 + 6n_6 + 7n_7 + 8n_8 + 9n_9 + 10n_{10}} \tag{21i}$$

7.3 Equations - With Whole Genome Duplication

7.3.1 Generalised solution for WGD followed by gain

The generalised solution for scenarios in which a whole genome duplication is followed by a series of gains of the same chromosome, such that all multiplicities are observed, as in scenario S_1 in Figure ST1 can be summarised as follows:

$$\begin{aligned} &\text{For copy number } (j+2) \text{ where events are } WGD + (j-2) \text{ Gains} \\ &x = (1, ..., j-2, j-1) \end{aligned} \tag{22}$$

$$t_x = \begin{cases} \dfrac{(j+2) \sum\limits_{k=j+1-x}^{j} n_k}{\sum\limits_{m=1}^{j} m n_m}, & \text{if } x = (1, ..., j-2) \\ \dfrac{(j+2) \sum\limits_{k=j+1-x}^{j-1} n_k}{\sum\limits_{m=1}^{j} m n_m}, & \text{if } x = (j-1) \end{cases}$$

7.3.2 2+0

$n_1 = 2m(1 - t_1)$
$n_2 = mt_1$

$$t_1 = t_W = \frac{2n_2}{n_1 + 2n_2} \tag{23}$$

7.3.3 2+1

$n_1 = m(3 - 2t_1)$
$n_2 = mt_1$

$$t_1 = t_W = \frac{3n_2}{n_1 + 2n_2} \tag{24}$$

7.3.4 2+2

$n_1 = 4m(1 - t_1)$
$n_2 = 2mt_1$

$$t_1 = t_W = \frac{2n_2}{n1 + 2n_2} \tag{25}$$

7.3.5 3+0

7.3.5.1 3+0:WG

$n_1 = m(3 - t_1 - 2t_2)$
$n_2 = m(t_2 - t_1)$
$n_3 = mt_1$

$$t_1 = t_W = \frac{3n_3}{n_1 + 2n_2 + 3n_3} \tag{26a}$$

$$t_2 = t_G = \frac{3(n_2 + n_3)}{n_1 + 2n_2 + 3n_3} \tag{26b}$$

7.3.6 3+1

7.3.6.1 3+1: WG

$n_1 = m(4 - t_1 - 2t_2)$
$n_2 = m(t_2 - t_1)$
$n_3 = mt_1$

$$t_1 = t_W = \frac{4n_3}{n_1 + 2n_2 + 3n_3} \tag{27a}$$

$$t_2 = t_G = \frac{4(n_2 + n_3)}{n_1 + 2n_2 + 3n_3} \tag{27b}$$

7.3.7 3+2

7.3.7.1 3+2: WG

$n_1 = m(5 - 3t_1 - 2t_2)$
$n_2 = mt_2$
$n_3 = mt_1$

$$t_1 = t_W = \frac{5n_3}{n_1 + 2n_2 + 3n_3} \tag{28a}$$

$$t_2 = t_G = \frac{5n_2}{n_1 + 2n_2 + 3n_3} \tag{28b}$$

7.3.8 4+0

7.3.8.1 4+0: WGG

$n_1 = m(4 - t_1 - t_2 - 2t_3)$
$n_2 = m(t_3 - t_2)$
$n_3 = m(t_2 - t_1)$
$n_4 = mt_1$

$$t_1 = t_W = \frac{4n_4}{n_1 + 2n_2 + 3n_3 + 4n_4} \tag{29a}$$

$$t_1 = t_{G1} = \frac{4(n_3 + n_4)}{n_1 + 2n_2 + 3n_3 + 4n_4} \tag{29b}$$

$$t_1 = t_{G2} = \frac{4(n_2 + n_3 + n_4)}{n_1 + 2n_2 + 3n_3 + 4n_4} \tag{29c}$$

7.3.8.2 4+0: GW

$n_1 = m(4 - 4t_2)$
$n_2 = m(2t_2 - 2t_1)$
$n_3 = 0$
$n_4 = mt_1$

$$t_1 = t_G = \frac{4n_4}{n_1 + 2n_2 + 4n_4} \tag{30a}$$

$$t_1 = t_W = \frac{2(n_2 + 2n_4)}{n_1 + 2n_2 + 4n_4} \tag{30b}$$

7.3.9 4+1

7.3.9.1 4+1: WGG

$n_1 = m(5 - t_1 - t_2 - 2t_3)$
$n_2 = m(t_3 - t_2)$
$n_3 = m(t_2 - t_1)$
$n_4 = mt_1$

$$t_1 = t_W = \frac{5n_4}{n_1 + 2n_2 + 3n_3 + 4n_4} \tag{31a}$$

$$t_2 = t_{G1} = \frac{5(n_3 + n_4)}{n_1 + 2n_2 + 3n_3 + 4n_4} \tag{31b}$$

$$t_3 = t_{G2} = \frac{5(n_2 + n_3 + n_4)}{n_1 + 2n_2 + 3n_3 + 4n_4} \tag{31c}$$

7.3.9.2 4+1: GW

$n_1 = m(5 - 4t_2)$
$n_2 = m(2t_2 - 2t_1)$
$n_3 = 0$
$n_4 = mt_1$

$$t_1 = t_G = \frac{5n_4}{n_1 + 2n_2 + 4n_4} \tag{32a}$$

$$t_2 = t_W = \frac{5(n_2 + 2n_4)}{2n_1 + 4n_2 + 8n_4} \tag{32b}$$

7.3.10 4+2

7.3.10.1 4+2: WGG

$n_1 = m(6 - 3t_1 - t_2 - 2t_3)$
$n_2 = m(t_1 + t_3 - t_2)$
$n_3 = m(t_2 - t_1)$
$n_4 = mt_1$

$$t_1 = t_W = \frac{6n_4}{n_1 + 2n_2 + 3n_3 + 4n_4} \tag{33a}$$

$$t_2 = t_{G1} = \frac{6(n_3 + n_4)}{n_1 + 2n_2 + 3n_3 + 4n_4} \tag{33b}$$

$$t_3 = t_{G2} = \frac{6(n_2 + n_3)}{n_1 + 2n_2 + 3n_3 + 4n_4} \tag{33c}$$

7.3.10.2 4+2: GW

$n_1 = m(6 - 6t_2)$
$n_2 = m(3t_2 - 2t_1)$
$n_3 = 0$
$n_4 = mt_1$

$$t_1 = t_G = \frac{6n_4}{n_1 + 2n_2 + 4n_4} \tag{34a}$$

$$t_2 = t_W = \frac{2(n_2 + 2n_4)}{n_1 + 2n_2 + 4n_4} \tag{34b}$$

7.3.11 5+0

7.3.11.1 5+0: WGGG

$n_1 = m(5 - t_1 - t_2 - t_3 - 2t_4)$
$n_2 = m(t_4 - t_3)$
$n_3 = m(t_3 - t_2)$
$n_4 = m(t_2 - t_1)$
$n_5 = mt_1$

$$t_1 = t_W = \frac{5n_5}{n_1 + 2n_2 + 3n_3 + 4n_4 + 5n_5} \tag{35a}$$

$$t_2 = t_{G1} = \frac{5(n_4 + n_5)}{n_1 + 2n_2 + 3n_3 + 4n_4 + 5n_5} \tag{35b}$$

$$t_3 = t_{G2} = \frac{5(n_3 + n_4 + n_5)}{n_1 + 2n_2 + 3n_3 + 4n_4 + 5n_5} \tag{35c}$$

$$t_4 = t_{G3} = \frac{5(n_2 + n_3 + n_4 + n_5)}{n_1 + 2n_2 + 3n_3 + 4n_4 + 5n_5} \tag{35d}$$

7.3.11.2 5+0: GWG

$n_1 = m(5 - 3t_2 - 2t_3)$
$n_2 = m(t_3 - t_1)$
$n_3 = m(t_2 - t_1)$
$n_4 = 0$
$n_5 = mt_1$

$$t_1 = t_{G1} = \frac{5n_5}{n_1 + 2n_2 + 3n_3 + 5n_5} \tag{36a}$$

$$t_1 = t_W = \frac{5(n_3 + n_5)}{n_1 + 2n_2 + 3n_3 + 5n_5} \tag{36b}$$

$$t_1 = t_{G2} = \frac{5(n_2 + n_5)}{n_1 + 2n_2 + 3n_3 + 5n_5} \tag{36c}$$

7.3.12 5+1

7.3.12.1 5+1: WGGG

$n_1 = m(6 - t_1 - t_2 - t_3 - 2t_4)$
$n_2 = m(t_4 - t_3)$
$n_3 = m(t_3 - t_2)$
$n_4 = m(t_2 - t_1)$
$n_5 = mt_1$

$$t_1 = t_W = \frac{6n_5}{n_1 + 2n_2 + 3n_3 + 4n_4 + 5n_5} \tag{37a}$$

$$t_2 = t_{G1} = \frac{6(n_4 + n_5)}{n_1 + 2n_2 + 3n_3 + 4n_4 + 5n_5} \tag{37b}$$

$$t_3 = t_{G2} = \frac{6(n_3 + n_4 + n_5)}{n_1 + 2n_2 + 3n_3 + 4n_4 + 5n_5} \tag{37c}$$

$$t_4 = t_{G3} = \frac{6(n_2 + n_3 + n_4 + n_5)}{n_1 + 2n_2 + 3n_3 + 4n_4 + 5n_5} \tag{37d}$$

7.3.12.2 5+1: GWG

$n_1 = m(6 - 3t_2 - 2t_3)$
$n_2 = m(t_3 - t_1)$
$n_3 = m(t_2 - t_1)$
$n_4 = 0$
$n_5 = mt_1$

$$t_1 = t_{G1} = \frac{6n_5}{n_1 + 2n_2 + 3n_3 + 5n_5} \tag{38a}$$

$$t_2 = t_W = \frac{6(n_3 + n_5)}{n_1 + 2n_2 + 3n_3 + 5n_5} \tag{38b}$$

$$t_3 = t_{G2} = \frac{6(n_2 + n_5)}{n_1 + 2n_2 + 3n_3 + 5n_5} \tag{38c}$$

7.3.13 5+2

7.3.13.1 5+2: WGGG

$n_1 = m(7 - 3t_1 - t_2 - t_3 - 2t_4)$
$n_2 = m(t_1 + t_4 - t_3)$
$n_3 = m(t_3 - t_2)$
$n_4 = m(t_2 - t_1)$
$n_5 = m(t_1)$

$$t_1 = t_W = \frac{7n_5}{n_1 + 2n_2 + 3n_3 + 4n_4 + 5n_5} \tag{39a}$$

$$t_2 = t_{G1} = \frac{7(n_4 + n_5)}{n_1 + 2n_2 + 3n_3 + 4n_4 + 5n_5} \tag{39b}$$

$$t_3 = t_{G2} = \frac{7(n_3 + n_4 + n_5)}{n_1 + 2n_2 + 3n_3 + 4n_4 + 5n_5} \tag{39c}$$

$$t_4 = t_{G3} = \frac{7(n_2 + n_3 + n_4)}{n_1 + 2n_2 + 3n_3 + 4n_4 + 5n_5} \tag{39d}$$

7.3.13.2 5+2: GWG

$n_1 = m(7 - 5t_2 - 2t_3)$
$n_2 = m(t_2 - t_1 + t_3)$
$n_3 = m(t_2 - t_1)$
$n_4 = 0$
$n_5 = mt_1$

$$t_1 = t_{G1} = \frac{7n_5}{n_1 + 2n_2 + 3n_3 + 5n_5} \tag{40a}$$

$$t_2 = t_W = \frac{7(n_3 + n_5)}{n_1 + 2n_2 + 3n_3 + 5n_5} \tag{40b}$$

$$t_3 = t_{G2} = \frac{7(n_2 - n_3)}{n_1 + 2n_2 + 3n_3 + 5n_5} \tag{40c}$$

7.3.14 6+0

7.3.14.1 6+0: WGGGG

$n_1 = m(6 - t_1 - t_2 - t_3 - t_4 - 2t_5)$
$n_2 = m(t_5 - t_4)$
$n_3 = m(t_4 - t_3)$
$n_4 = m(t_3 - t_2)$
$n_5 = m(t_2 - t_1)$
$n_6 = mt_1$

$$t_1 = t_W = \frac{6n_6}{n_1 + 2n_2 + 3n_3 + 4n_4 + 5n_5 + 6n_6} \tag{41a}$$

$$t_2 = t_{G1} = \frac{6(n_5 + n_6)}{n_1 + 2n_2 + 3n_3 + 4n_4 + 5n_5 + 6n_6} \tag{41b}$$

$$t_3 = t_{G2} = \frac{6(n_4 + n_5 + n_6)}{n_1 + 2n_2 + 3n_3 + 4n_4 + 5n_5 + 6n_6} \tag{41c}$$

$$t_4 = t_{G3} = \frac{6(n_3 + n_4 + n_5 + n_6)}{n_1 + 2n_2 + 3n_3 + 4n_4 + 5n_5 + 6n_6} \tag{41d}$$

$$t_5 = t_{G4} = \frac{6(n_2 + n_3 + n_4 + n_5 + n_6)}{n_1 + 2n_2 + 3n_3 + 4n_4 + 5n_5 + 6n_6} \tag{41e}$$

7.3.14.2 6+0: GWGG

$n_1 = m(6 - 3t_2 - t_3 - 2t_4)$
$n_2 = m(t_2 - t_1 + t_4 - t_3)$
$n_3 = m(t_3 - t_2)$
$n_4 = m(t_2 - t_1)$
$n_5 = 0$
$n_6 = mt_1$

$$t_1 = t_{G1} = \frac{6n_6}{n_1 + 2n_2 + 3n_3 + 4n_4 + 6n_6} \tag{42a}$$

$$t_2 = t_W = \frac{6(n_4 + n_6)}{n_1 + 2n_2 + 3n_3 + 4n_4 + 6n_6} \tag{42b}$$

$$t_3 = t_{G2} = \frac{6(n_3 + n_4 + n_6)}{n_1 + 2n_2 + 3n_3 + 4n_4 + 6n_6} \tag{42c}$$

$$t_4 = t_{G3} = \frac{6(n_2 + n_3 + n_6)}{n_1 + 2n_2 + 3n_3 + 4n_4 + 6n_6} \tag{42d}$$

7.3.14.3 6+0: GGW

$n_1 = m(6 - 6t_3)$
$n_2 = m(3t_3 - 2t_2 - t_1)$
$n_3 = 0$
$n_4 = m(t_2 - t_1)$
$n_5 = 0$
$n_6 = mt_1$

$$t_1 = t_{G1} = \frac{6n_6}{n_1 + 2n_2 + 4n_4 + 6n_6} \tag{43a}$$

$$t_2 = t_{G2} = \frac{6(n_4 + n_6)}{n_1 + 2n_2 + 4n_4 + 6n_6} \tag{43b}$$

$$t_3 = t_W = \frac{2(n_2 + 2n_4 + 3n_6)}{n_1 + 2n_2 + 4n_4 + 6n_6} \tag{43c}$$

7.3.15 6+1

7.3.15.1 6+1: WGGGG

$n_1 = m(7 - t_1 - t_2 - t_3 - t_4 - 2t_5)$
$n_2 = m(t_5 - t_4)$
$n_3 = m(t_4 - t_3)$
$n_4 = m(t_3 - t_2)$
$n_5 = m(t_2 - t_1)$
$n_6 = mt_1$

$$t_1 = t_W = \frac{7n_6}{n_1 + 2n_2 + 3n_3 + 4n_4 + 5n_5 + 6n_6} \tag{44a}$$

$$t_2 = t_{G1} = \frac{7(n_5 + n_6)}{n_1 + 2n_2 + 3n_3 + 4n_4 + 5n_5 + 6n_6} \tag{44b}$$

$$t_3 = t_{G2} = \frac{7(n_4 + n_5 + n_6)}{n_1 + 2n_2 + 3n_3 + 4n_4 + 5n_5 + 6n_6} \tag{44c}$$

$$t_4 = t_{G3} = \frac{7(n_3 + n_4 + n_5 + n_6)}{n_1 + 2n_2 + 3n_3 + 4n_4 + 5n_5 + 6n_6} \tag{44d}$$

$$t_5 = t_{G4} = \frac{7(n_2 + n_3 + n_4 + n_5 + n_6)}{n_1 + 2n_2 + 3n_3 + 4n_4 + 5n_5 + 6n_6} \tag{44e}$$

7.3.15.2 6+1: GWGG

$n_1 = m(7 - 3t_2 - t_3 - 2t_4)$
$n_2 = m(-t_1 + t_2 - t_3 + t_4)$
$n_3 = m(t_3 - t_2)$
$n_4 = m(t_2 - t_1)$
$n_5 = 0$
$n_6 = mt_1$

$$t_1 = t_{G1} = \frac{7n_6}{n_1 + 2n_2 + 3n_3 + 4n_4 + 6n_6} \tag{45a}$$

$$t_2 = t_W = \frac{7(n_4 + n_6)}{n_1 + 2n_2 + 3n_3 + 4n_4 + 6n_6} \tag{45b}$$

$$t_3 = t_{G2} = \frac{7(n_3 + n_4 + n_6)}{n_1 + 2n_2 + 3n_3 + 4n_4 + 6n_6} \tag{45c}$$

$$t_4 = t_{G3} = \frac{7(n_2 + n_3 + n_6)}{n_1 + 2n_2 + 3n_3 + 4n_4 + 6n_6} \tag{45d}$$

7.3.15.3 6+1: GGW

$n_1 = m(7 - 6t_3)$
$n_2 = m(3t_3 - 2t_2 - t_1)$
$n_3 = 0$
$n_4 = m(t_2 - t_1)$
$n_5 = 0$
$n_6 = mt_1$

$$t_1 = t_{G1} = \frac{7n_6}{n_1 + 2n_2 + 4n_4 + 6n_6} \tag{46a}$$

$$t_2 = t_{G2} = \frac{7(n_4 + n_6)}{n_1 + 2n_2 + 4n_4 + 6n_6} \tag{46b}$$

$$t_3 = t_W = \frac{7(n_2 + 2n_4 + 3n_6)}{3(n_1 + 2n_2 + 4n_4 + 6n_6)} \tag{46c}$$

7.3.16 6+2

7.3.16.1 6+2: WGGGG

$n_1 = m(8 - 3t_1 - t_2 - t_3 - t_4 - 2t_5)$
$n_2 = m(t_1 + t_5 - t_4)$
$n_3 = m(t_4 - t_3)$
$n_4 = m(t_3 - t_2)$
$n_5 = m(t_2 - t_1)$
$n_6 = mt_1$

$$t_1 = t_W = \frac{8n_6}{n_1 + 2n_2 + 3n_3 + 4n_4 + 5n_5 + 6n_6} \tag{47a}$$

$$t_2 = t_{G1} = \frac{8(n_5 + n_6)}{n_1 + 2n_2 + 3n_3 + 4n_4 + 5n_5 + 6n_6} \tag{47b}$$

$$t_3 = t_{G2} = \frac{8(n_4 + n_5 + n_6)}{n_1 + 2n_2 + 3n_3 + 4n_4 + 5n_5 + 6n_6} \tag{47c}$$

$$t_4 = t_{G3} = \frac{8(n_3 + n_4 + n_5 + n_6)}{n_1 + 2n_2 + 3n_3 + 4n_4 + 5n_5 + 6n_6} \tag{47d}$$

$$t_5 = t_{G4} = \frac{8(n_2 + n_3 + n_4 + n_5)}{n_1 + 2n_2 + 3n_3 + 4n_4 + 5n_5 + 6n_6} \tag{47e}$$

7.3.16.2 6+2: GWGG

$n_1 = m(8 - 5t_2 - t_3 - 2t_4)$
$n_2 = m(2t_2 - t_1 + t_4 - t_3)$
$n_3 = m(t_3 - t_2)$
$n_4 = m(t_2 - t_1)$
$n_5 = 0$
$n_6 = mt_1$

$$t_1 = t_{G1} = \frac{8n_6}{n_1 + 2n_2 + 3n_3 + 4n_4 + 6n_6} \tag{48a}$$

$$t_2 = t_W = \frac{8(n_4 + n_6)}{n_1 + 2n_2 + 3n_3 + 4n_4 + 6n_6} \tag{48b}$$

$$t_3 = t_{G2} = \frac{8(n_3 + n_4 + n_6)}{n_1 + 2n_2 + 3n_3 + 4n_4 + 6n_6} \tag{48c}$$

$$t_4 = t_{G3} = \frac{8(n_2 + n_3 - n_4)}{n_1 + 2n_2 + 3n_3 + 4n_4 + 6n_6} \tag{48d}$$

7.3.16.3 6+2: GGW

$n_1 = m(8 - 8t_3)$
$n_2 = m(4t_3 - 2t_2 - t_1)$
$n_3 = 0$
$n_4 = m(t_2 - t_1)$
$n_5 = 0$
$n_6 = mt_1$

$$t_1 = t_{G1} = \frac{8n_6}{n_1 + 2n_2 + 4n_4 + 6n_6} \tag{49a}$$

$$t_2 = t_{G2} = \frac{8(n_4 + n_6)}{n_1 + 2n_2 + 4n_4 + 6n_6} \tag{49b}$$

$$t_3 = t_W = \frac{2(n_2 + 2n_4 + 3n_6)}{n_1 + 2n_2 + 4n_4 + 6n_6} \tag{49c}$$

7.3.17 7+0

7.3.17.1 7+0: WGGGGG

$n_1 = m(7 - t_1 - t_2 - t_3 - t_4 - t_5 - 2t_6)$
$n_2 = m(t_6 - t_5)$
$n_3 = m(t_5 - t_4)$
$n_4 = m(t_4 - t_3)$
$n_5 = m(t_3 - t_2)$
$n_6 = m(t_2 - t_1)$
$n_7 = mt_1$

$$t_1 = t_W = \frac{7n_7}{n_1 + 2n_2 + 3n_3 + 4n_4 + 5n_5 + 6n_6 + 7n_7} \tag{50a}$$

$$t_2 = t_{G1} = \frac{7(n_6 + n_7)}{n_1 + 2n_2 + 3n_3 + 4n_4 + 5n_5 + 6n_6 + 7n_7} \tag{50b}$$

$$t_3 = t_{G2} = \frac{7(n_5 + n_6 + n_7)}{n_1 + 2n_2 + 3n_3 + 4n_4 + 5n_5 + 6n_6 + 7n_7} \tag{50c}$$

$$t_4 = t_{G3} = \frac{7(n_4 + n_5 + n_6 + n_7)}{n_1 + 2n_2 + 3n_3 + 4n_4 + 5n_5 + 6n_6 + 7n_7} \tag{50d}$$

$$t_5 = t_{G4} = \frac{7(n_3 + n_4 + n_5 + n_6 + n_7)}{n_1 + 2n_2 + 3n_3 + 4n_4 + 5n_5 + 6n_6 + 7n_7} \tag{50e}$$

$$t_6 = t_{G5} = \frac{7(n_2 + n_3 + n_4 + n_5 + n_6 + n_7)}{n_1 + 2n_2 + 3n_3 + 4n_4 + 5n_5 + 6n_6 + 7n_7} \tag{50f}$$

7.3.17.2 7+0: GWGGG

$n_1 = m(7 - 3t_2 - t_3 - t_4 - 2t_5)$
$n_2 = m(t_2 - t_1 + t_5 - t_4)$
$n_3 = m(t_4 - t_3)$
$n_4 = m(t_3 - t_2)$
$n_5 = m(t_2 - t_1)$
$n_6 = 0$
$n_7 = mt_1$

$$t_1 = t_{G1} = \frac{7n_7}{n_1 + 2n_2 + 3n_3 + 4n_4 + 5n_5 + 7n_7} \tag{51a}$$

$$t_2 = t_W = \frac{7(n_5 + n_7)}{n_1 + 2n_2 + 3n_3 + 4n_4 + 5n_5 + 7n_7} \tag{51b}$$

$$t_3 = t_{G2} = \frac{7(n_4 + n_5 + n_7)}{n_1 + 2n_2 + 3n_3 + 4n_4 + 5n_5 + 7n_7} \tag{51c}$$

$$t_4 = t_{G3} = \frac{7(n_3 + n_4 + n_5 + n_7)}{n_1 + 2n_2 + 3n_3 + 4n_4 + 5n_5 + 7n_7} \tag{51d}$$

$$t_5 = t_{G4} = \frac{7(n_2 + n_3 + n_4 + n_7)}{n_1 + 2n_2 + 3n_3 + 4n_4 + 5n_5 + 7n_7} \tag{51e}$$

7.3.17.3 7+0: GGWG

$n_1 = m(7 - 5t_3 - 2t_4)$
$n_2 = m(t_4 - t_1 - t_2 + t_3)$
$n_3 = m(t_3 - t_2)$
$n_4 = 0$
$n_5 = m(t_2 - t_1)$
$n_6 = 0$
$n_7 = mt_1$

$$t_1 = t_{G1} = \frac{7n_7}{n_1 + 2n_2 + 3n_3 + 5n_5 + 7n_7} \tag{52a}$$

$$t_2 = t_{G2} = \frac{7(n_5 + n_7)}{n_1 + 2n_2 + 3n_3 + 5n_5 + 7n_7} \tag{52b}$$

$$t_3 = t_W = \frac{7(n_3 + n_5 + n_7)}{n_1 + 2n_2 + 3n_3 + 5n_5 + 7n_7} \tag{52c}$$

$$t_4 = t_{G3} = \frac{7(n_2 - n_3 + n_7)}{n_1 + 2n_2 + 3n_3 + 5n_5 + 7n_7} \tag{52d}$$

7.3.18 7+1

7.3.18.1 7+1: WGGGGG

$n_1 = m(8 - t_1 - t_2 - t_3 - t_4 - t_5 - 2t_6)$
$n_2 = m(t_6 - t_5)$
$n_3 = m(t_5 - t_4)$
$n_4 = m(t_4 - t_3)$
$n_5 = m(t_3 - t_2)$
$n_6 = m(t_2 - t_1)$
$n_7 = mt_1$

$$t_1 = t_W = \frac{8n_7}{n_1 + 2n_2 + 3n_3 + 4n_4 + 5n_5 + 6n_6 + 7n_7} \tag{53a}$$

$$t_2 = t_{G1} = \frac{8(n_6 + n_7)}{n_1 + 2n_2 + 3n_3 + 4n_4 + 5n_5 + 6n_6 + 7n_7} \tag{53b}$$

$$t_3 = t_{G2} = \frac{8(n_5 + n_6 + n_7)}{n_1 + 2n_2 + 3n_3 + 4n_4 + 5n_5 + 6n_6 + 7n_7} \tag{53c}$$

$$t_4 = t_{G3} = \frac{8(n_4 + n_5 + n_6 + n_7)}{n_1 + 2n_2 + 3n_3 + 4n_4 + 5n_5 + 6n_6 + 7n_7} \tag{53d}$$

$$t_5 = t_{G4} = \frac{8(n_3 + n_4 + n_5 + n_6 + n_7)}{n_1 + 2n_2 + 3n_3 + 4n_4 + 5n_5 + 6n_6 + 7n_7} \tag{53e}$$

$$t_6 = t_{G5} = \frac{8(n_2 + n_3 + n_4 + n_5 + n_6 + n_7)}{n_1 + 2n_2 + 3n_3 + 4n_4 + 5n_5 + 6n_6 + 7n_7} \tag{53f}$$

7.3.18.2 7+1: GWGGG

$n_1 = m(8 - 3t_2 - t_3 - t_4 - 2t_5)$
$n_2 = m(t_2 - t_1 + t_5 - t_4)$
$n_3 = m(t_4 - t_3)$
$n_4 = m(t_3 - t_2)$
$n_5 = m(t_2 - t_1)$
$n_6 = 0$
$n_7 = mt_1$

$$t_1 = t_{G1} = \frac{8n_7}{n_1 + 2n_2 + 3n_3 + 4n_4 + 5n_5 + 7n_7} \tag{54a}$$

$$t_2 = t_W = \frac{8(n_5 + n_7)}{n_1 + 2n_2 + 3n_3 + 4n_4 + 5n_5 + 7n_7} \tag{54b}$$

$$t_3 = t_{G2} = \frac{8(n_4 + n_5 + n_7)}{n_1 + 2n_2 + 3n_3 + 4n_4 + 5n_5 + 7n_7} \tag{54c}$$

$$t_4 = t_{G3} = \frac{8(n_3 + n_4 + n_5 + n_7)}{n_1 + 2n_2 + 3n_3 + 4n_4 + 5n_5 + 7n_7} \tag{54d}$$

$$t_5 = t_{G3} = \frac{8(n_2 + n_3 + n_4 + n_7)}{n_1 + 2n_2 + 3n_3 + 4n_4 + 5n_5 + 7n_7} \tag{54e}$$

7.3.18.3 7+1: GGWG

$n_1 = m(8 - 5t_3 - 2t_4)$
$n_2 = m(t_3 - t_2 - t_1 + t_4)$
$n_3 = m(t_3 - t_2)$
$n_4 = 0$
$n_5 = m(t_2 - t_1)$
$n_6 = 0$
$n_7 = mt_1$

$$t_1 = t_{G1} = \frac{8n_7}{n_1 + 2n_2 + 3n_3 + 5n_5 + 7n_7} \tag{55a}$$

$$t_2 = t_{G2} = \frac{8(n_5 + n_7)}{n_1 + 2n_2 + 3n_3 + 5n_5 + 7n_7} \tag{55b}$$

$$t_3 = t_W = \frac{8(n_3 + n_5 + n_7)}{n_1 + 2n_2 + 3n_3 + 5n_5 + 7n_7} \tag{55c}$$

$$t_4 = t_{G3} = \frac{8(n_2 - n_3 + n_7)}{n_1 + 2n_2 + 3n_3 + 5n_5 + 7n_7} \tag{55d}$$

7.3.19 7+2

7.3.19.1 7+2: WGGGGG

$n_1 = m(9 - 3t_1 - t_2 - t_3 - t_4 - t_5 - 2t_6)$
$n_2 = m(t_1 + t_6 - t_5)$
$n_3 = m(t_5 - t_4)$
$n_4 = m(t_4 - t_3)$
$n_5 = m(t_3 - t_2)$
$n_6 = m(t_2 - t_1)$
$n_7 = mt_1$

$$t_1 = t_W = \frac{9n_7}{n_1 + 2n_2 + 3n_3 + 4n_4 + 5n_5 + 6n_6 + 7n_7} \tag{56a}$$

$$t_2 = t_{G1} = \frac{9(n_6 + n_7)}{n_1 + 2n_2 + 3n_3 + 4n_4 + 5n_5 + 6n_6 + 7n_7} \tag{56b}$$

$$t_3 = t_{G2} = \frac{9(n_5 + n_6 + n_7)}{n_1 + 2n_2 + 3n_3 + 4n_4 + 5n_5 + 6n_6 + 7n_7} \tag{56c}$$

$$t_4 = t_{G3} = \frac{9(n_4 + n_5 + n_6 + n_7)}{n_1 + 2n_2 + 3n_3 + 4n_4 + 5n_5 + 6n_6 + 7n_7} \tag{56d}$$

$$t_5 = t_{G4} = \frac{9(n_3 + n_4 + n_5 + n_6 + n_7)}{n_1 + 2n_2 + 3n_3 + 4n_4 + 5n_5 + 6n_6 + 7n_7} \tag{56e}$$

$$t_6 = t_{G5} = \frac{9(n_2 + n_3 + n_4 + n_5 + n_6)}{n_1 + 2n_2 + 3n_3 + 4n_4 + 5n_5 + 6n_6 + 7n_7} \tag{56f}$$

7.3.19.2 7+2: GWGGG

$n_1 = m(9 - 5t_2 - t_3 - t_4 - 2t_5)$
$n_2 = m(2t_2 - t_1 + t_5 - t_4)$
$n_3 = m(t_4 - t_3)$
$n_4 = m(t_3 - t_2)$
$n_5 = m(t_2 - t_1)$
$n_6 = 0$
$n_7 = mt_1$

$$t_1 = t_{G1} = \frac{9n_7}{n_1 + 2n_2 + 3n_3 + 4n_4 + 5n_5 + 7n_7} \tag{57a}$$

$$t_2 = t_W = \frac{9(n_5 + n_7)}{n_1 + 2n_2 + 3n_3 + 4n_4 + 5n_5 + 7n_7} \tag{57b}$$

$$t_3 = t_{G2} = \frac{9(n_4 + n_5 + n_7)}{n_1 + 2n_2 + 3n_3 + 4n_4 + 5n_5 + 7n_7} \tag{57c}$$

$$t_4 = t_{G3} = \frac{9(n_3 + n_4 + n_5 + n_7)}{n_1 + 2n_2 + 3n_3 + 4n_4 + 5n_5 + 7n_7} \tag{57d}$$

$$t_5 = t_{G3} = \frac{9(n_2 + n_3 + n_4 - n_5)}{n_1 + 2n_2 + 3n_3 + 4n_4 + 5n_5 + 7n_7} \tag{57e}$$

7.3.19.3 7+2: GGWG

$n_1 = m(9 - 7t_3 - 2t_4)$
$n_2 = m(2t_3 - t_2 - t_1 + t_4)$
$n_3 = m(t_3 - t_2)$
$n_4 = 0$
$n_5 = m(t_2 - t_1)$
$n_6 = 0$
$n_7 = mt_1$

$$t_1 = t_{G1} = \frac{9n_7}{n_1 + 2n_2 + 3n_3 + 5n_5 + 7n_7} \tag{58a}$$

$$t_2 = t_{G2} = \frac{9(n_5 + n_7)}{n_1 + 2n_2 + 3n_3 + 5n_5 + 7n_7} \tag{58b}$$

$$t_3 = t_W = \frac{9(n_3 + n_5 + n_7)}{n_1 + 2n_2 + 3n_3 + 5n_5 + 7n_7} \tag{58c}$$

$$t_4 = t_{G3} = \frac{9(n_2 - 2n_3 - n_5)}{n_1 + 2n_2 + 3n_3 + 5n_5 + 7n_7} \tag{58d}$$

7.3.20 8+0

7.3.20.1 8+0: WGGGGGG

$n_1 = m(8 - t_1 - t_2 - t_3 - t_4 - t_5 - t_6 - 2t_7)$
$n_2 = m(t_7 - t_6)$
$n_3 = m(t_6 - t_5)$
$n_4 = m(t_5 - t_4)$
$n_5 = m(t_4 - t_3)$
$n_6 = m(t_3 - t_2)$
$n_7 = m(t_2 - t_1)$
$n_8 = mt_1$

$$t_1 = t_W = \frac{8n_8}{n_1 + 2n_2 + 3n_3 + 4n_4 + 5n_5 + 6n_6 + 7n_7 + 8n_8} \tag{59a}$$

$$t_2 = t_{G1} = \frac{8(n_7 + n_8)}{n_1 + 2n_2 + 3n_3 + 4n_4 + 5n_5 + 6n_6 + 7n_7 + 8n_8} \tag{59b}$$

$$t_3 = t_{G2} = \frac{8(n_6 + n_7 + n_8)}{n_1 + 2n_2 + 3n_3 + 4n_4 + 5n_5 + 6n_6 + 7n_7 + 8n_8} \tag{59c}$$

$$t_4 = t_{G3} = \frac{8(n_5 + n_6 + n_7 + n_8)}{n_1 + 2n_2 + 3n_3 + 4n_4 + 5n_5 + 6n_6 + 7n_7 + 8n_8} \tag{59d}$$

$$t_5 = t_{G4} = \frac{8(n_4 + n_5 + n_6 + n_7 + n_8)}{n_1 + 2n_2 + 3n_3 + 4n_4 + 5n_5 + 6n_6 + 7n_7 + 8n_8} \tag{59e}$$

$$t_6 = t_{G5} = \frac{8(n_3 + n_4 + n_5 + n_6 + n_7 + n_8)}{n_1 + 2n_2 + 3n_3 + 4n_4 + 5n_5 + 6n_6 + 7n_7 + 8n_8} \tag{59f}$$

$$t_7 = t_{G6} = \frac{8(n_2 + n_3 + n_4 + n_5 + n_6 + n_7 + n_8)}{n_1 + 2n_2 + 3n_3 + 4n_4 + 5n_5 + 6n_6 + 7n_7 + 8n_8} \tag{59g}$$

7.3.20.2 8+0: GWGGGG

$n_1 = m(8 - 3t_2 - t_3 - t_4 - t_5 - 2t_6)$
$n_2 = m(t_2 - t_1 + t_6 - t_5)$
$n_3 = m(t_5 - t_4)$
$n_4 = m(t_4 - t_3)$
$n_5 = m(t_3 - t_2)$
$n_6 = m(t_2 - t_1)$
$n_7 = 0$
$n_8 = mt_1$

$$t_1 = t_{G1} = \frac{8n_8}{n_1 + 2n_2 + 3n_3 + 4n_4 + 5n_5 + 6n_6 + 8n_8} \tag{60a}$$

$$t_2 = t_W = \frac{8(n_6 + n_8)}{n_1 + 2n_2 + 3n_3 + 4n_4 + 5n_5 + 6n_6 + 8n_8} \tag{60b}$$

$$t_3 = t_{G2} = \frac{8(n_5 + n_6 + n_8)}{n_1 + 2n_2 + 3n_3 + 4n_4 + 5n_5 + 6n_6 + 8n_8} \tag{60c}$$

$$t_4 = t_{G3} = \frac{8(n_4 + n_5 + n_6 + n_8)}{n_1 + 2n_2 + 3n_3 + 4n_4 + 5n_5 + 6n_6 + 8n_8} \tag{60d}$$

$$t_5 = t_{G4} = \frac{8(n_3 + n_4 + n_5 + n_6 + n_8)}{n_1 + 2n_2 + 3n_3 + 4n_4 + 5n_5 + 6n_6 + 8n_8} \tag{60e}$$

$$t_6 = t_{G5} = \frac{8(n_2 + n_3 + n_4 + n_5 + n_8)}{n_1 + 2n_2 + 3n_3 + 4n_4 + 5n_5 + 6n_6 + 8n_8} \tag{60f}$$

7.3.20.3 8+0: GGWGG

$n_1 = m(8 - 5t_3 - t_4 - 2t_5)$
$n_2 = m(t_5 - t_1 - t_2 + 2t_3 - t_4)$
$n_3 = m(t_4 - t_3)$
$n_4 = m(t_3 - t_2)$
$n_5 = 0$
$n_6 = m(t_2 - t_1)$
$n_7 = 0$
$n_8 = mt_1$

$$t_1 = t_{G1} = \frac{8n_8}{n_1 + 2n_2 + 3n_3 + 4n_4 + 6n_6 + 8n_8} \tag{61a}$$

$$t_2 = t_{G2} = \frac{8(n_6 + n_8)}{n_1 + 2n_2 + 3n_3 + 4n_4 + 6n_6 + 8n_8} \tag{61b}$$

$$t_3 = t_W = \frac{8(n_4 + n_6 + n_8)}{n_1 + 2n_2 + 3n_3 + 4n_4 + 6n_6 + 8n_8} \tag{61c}$$

$$t_4 = t_{G3} = \frac{8(n_3 + n_4 + n_6 + n_8)}{n_1 + 2n_2 + 3n_3 + 4n_4 + 6n_6 + 8n_8} \tag{61d}$$

$$t_5 = t_{G4} = \frac{8(n_2 + n_3 - n_4 + n_8)}{n_1 + 2n_2 + 3n_3 + 4n_4 + 6n_6 + 8n_8} \tag{61e}$$

7.3.20.4 8+0: GGGW

$n_1 = m(8 - 8t_4)$
$n_2 = m(4t_4 - t_1 - t_2 - 2t_3)$
$n_3 = 0$
$n_4 = m(t_3 - t_2)$
$n_5 = 0$
$n_6 = m(t_2 - t_1)$
$n_7 = 0$
$n_8 = mt_1$

$$t_1 = t_{G1} = \frac{8n_8}{n_1 + 2n_2 + 4n_4 + 6n_6 + 8n_8} \tag{62a}$$

$$t_2 = t_{G2} = \frac{8(n_6 + n_8)}{n_1 + 2n_2 + 4n_4 + 6n_6 + 8n_8} \tag{62b}$$

$$t_3 = t_{G3} = \frac{8(n_4 + n_6 + n_8)}{n_1 + 2n_2 + 4n_4 + 6n_6 + 8n_8} \tag{62c}$$

$$t_4 = t_W = \frac{2(n_2 + 2n_4 + 3n_6 + 4n_8)}{n_1 + 2n_2 + 4n_4 + 6n_6 + 8n_8} \tag{62d}$$

7.3.21 8+1

7.3.21.1 8+1: WGGGGGG

$n_1 = m(9 - t_1 - t_2 - t_3 - t_4 - t_5 - t_6 - 2t_7)$
$n_2 = m(t_7 - t_6)$
$n_3 = m(t_6 - t_5)$
$n_4 = m(t_5 - t_4)$
$n_5 = m(t_4 - t_3)$
$n_6 = m(t_3 - t_2)$
$n_7 = m(t_2 - t_1)$
$n_8 = mt_1$

$$t_1 = t_W = \frac{9n_8}{n_1 + 2n_2 + 3n_3 + 4n_4 + 5n_5 + 6n_6 + 7n_7 + 8n_8} \tag{63a}$$

$$t_2 = t_{G1} = \frac{9(n_7 + n_8)}{n_1 + 2n_2 + 3n_3 + 4n_4 + 5n_5 + 6n_6 + 7n_7 + 8n_8} \tag{63b}$$

$$t_3 = t_{G2} = \frac{9(n_6 + n_7 + n_8)}{n_1 + 2n_2 + 3n_3 + 4n_4 + 5n_5 + 6n_6 + 7n_7 + 8n_8} \tag{63c}$$

$$t_4 = t_{G3} = \frac{9(n_5 + n_6 + n_7 + n_8)}{n_1 + 2n_2 + 3n_3 + 4n_4 + 5n_5 + 6n_6 + 7n_7 + 8n_8} \tag{63d}$$

$$t_5 = t_{G4} = \frac{9(n_4 + n_5 + n_6 + n_7 + n_8)}{n_1 + 2n_2 + 3n_3 + 4n_4 + 5n_5 + 6n_6 + 7n_7 + 8n_8} \tag{63e}$$

$$t_6 = t_{G5} = \frac{9(n_3 + n_4 + n_5 + n_6 + n_7 + n_8)}{n_1 + 2n_2 + 3n_3 + 4n_4 + 5n_5 + 6n_6 + 7n_7 + 8n_8} \tag{63f}$$

$$t_7 = t_{G6} = \frac{9(n_2 + n_3 + n_4 + n_5 + n_6 + n_7 + n_8)}{n_1 + 2n_2 + 3n_3 + 4n_4 + 5n_5 + 6n_6 + 7n_7 + 8n_8} \tag{63g}$$

7.3.21.2 8+1: GWGGGG

$n_1 = m(9 - 3t_2 - t_3 - t_4 - t_5 - 2t_6)$
$n_2 = m(t_2 - t_1 + t_6 - t_5)$
$n_3 = m(t_5 - t_4)$
$n_4 = m(t_4 - t_3)$
$n_5 = m(t_3 - t_2)$
$n_6 = m(t_2 - t_1)$
$n_7 = 0$
$n_8 = mt_1$

$$t_1 = t_{G1} = \frac{9n_8}{n_1 + 2n_2 + 3n_3 + 4n_4 + 5n_5 + 6n_6 + 8n_8} \tag{64a}$$

$$t_2 = t_W = \frac{9(n_6 + n_8)}{n_1 + 2n_2 + 3n_3 + 4n_4 + 5n_5 + 6n_6 + 8n_8} \tag{64b}$$

$$t_3 = t_{G2} = \frac{9(n_5 + n_6 + n_8)}{n_1 + 2n_2 + 3n_3 + 4n_4 + 5n_5 + 6n_6 + 8n_8} \tag{64c}$$

$$t_4 = t_{G3} = \frac{9(n_4 + n_5 + n_6 + n_8)}{n_1 + 2n_2 + 3n_3 + 4n_4 + 5n_5 + 6n_6 + 8n_8} \tag{64d}$$

$$t_5 = t_{G4} = \frac{9(n_3 + n_4 + n_5 + n_6 + n_8)}{n_1 + 2n_2 + 3n_3 + 4n_4 + 5n_5 + 6n_6 + 8n_8} \tag{64e}$$

$$t_6 = t_{G5} = \frac{9(n_2 + n_3 + n_4 + n_5 + n_8)}{n_1 + 2n_2 + 3n_3 + 4n_4 + 5n_5 + 6n_6 + 8n_8} \tag{64f}$$

7.3.21.3 8+1: GGWGG

$n_1 = m(9 - 5t_3 - t_4 - 2t_5)$
$n_2 = m(t_5 - t_1 - t_2 + 2t_3 - t_4)$
$n_3 = m(t_4 - t_3)$
$n_4 = m(t_3 - t_2)$
$n_5 = 0$
$n_6 = m(t_2 - t_1)$
$n_7 = 0$
$n_8 = mt_1$

$$t_1 = t_{G1} = \frac{9n_8}{n_1 + 2n_2 + 3n_3 + 4n_4 + 6n_6 + 8n_8} \tag{65a}$$

$$t_2 = t_{G2} = \frac{9(n_6 + n_8)}{n_1 + 2n_2 + 3n_3 + 4n_4 + 6n_6 + 8n_8} \tag{65b}$$

$$t_3 = t_W = \frac{9(n_4 + n_6 + n_8)}{n_1 + 2n_2 + 3n_3 + 4n_4 + 6n_6 + 8n_8} \tag{65c}$$

$$t_4 = t_{G3} = \frac{9(n_3 + n_4 + n_6 + n_8)}{n_1 + 2n_2 + 3n_3 + 4n_4 + 6n_6 + 8n_8} \tag{65d}$$

$$t_5 = t_{G4} = \frac{9(n_2 + n_3 - n_4 + n_8)}{n_1 + 2n_2 + 3n_3 + 4n_4 + 6n_6 + 8n_8} \tag{65e}$$

7.3.21.4 8+1: GGGW

$n_1 = m(9 - 8t_4)$
$n_2 = m(4t_4 - t_1 - t_2 - 2t_3)$
$n_3 = 0$
$n_4 = m(t_3 - t_2)$
$n_5 = 0$
$n_6 = m(t_2 - t_1)$
$n_7 = 0$
$n_8 = mt_1$

$$t_1 = t_{G1} = \frac{9n_8}{n_1 + 2n_2 + 4n_4 + 6n_6 + 8n_8} \tag{66a}$$

$$t_2 = t_{G2} = \frac{9(n_6 + n_8)}{n_1 + 2n_2 + 4n_4 + 6n_6 + 8n_8} \tag{66b}$$

$$t_3 = t_{G3} = \frac{9(n_4 + n_6 + n_8)}{n_1 + 2n_2 + 4n_4 + 6n_6 + 8n_8} \tag{66c}$$

$$t_4 = t_W = \frac{9(n_2 + 2n_4 + 3n_6 + 4n_8)}{4(n_1 + 2n_2 + 4n_4 + 6n_6 + 8n_8)} \tag{66d}$$

7.3.22 8+2

7.3.22.1 8+2: WGGGGGG

$n_1 = m(10 - 3t_1 - t_2 - t_3 - t_4 - t_5 - t_6 - 2t_7)$
$n_2 = m(t_1 + t_7 - t_6)$
$n_3 = m(t_6 - t_5)$
$n_4 = m(t_5 - t_4)$
$n_5 = m(t_4 - t_3)$
$n_6 = m(t_3 - t_2)$
$n_7 = m(t_2 - t_1)$
$n_8 = mt_1$

$$t_1 = t_W = \frac{10n_8}{n_1 + 2n_2 + 3n_3 + 4n_4 + 5n_5 + 6n_6 + 7n_7 + 8n_8} \tag{67a}$$

$$t_2 = t_{G1} = \frac{10(n_7 + n_8)}{n_1 + 2n_2 + 3n_3 + 4n_4 + 5n_5 + 6n_6 + 7n_7 + 8n_8} \tag{67b}$$

$$t_3 = t_{G2} = \frac{10(n_6 + n_7 + n_8)}{n_1 + 2n_2 + 3n_3 + 4n_4 + 5n_5 + 6n_6 + 7n_7 + 8n_8} \tag{67c}$$

$$t_4 = t_{G3} = \frac{10(n_5 + n_6 + n_7 + n_8)}{n_1 + 2n_2 + 3n_3 + 4n_4 + 5n_5 + 6n_6 + 7n_7 + 8n_8} \tag{67d}$$

$$t_5 = t_{G4} = \frac{10(n_4 + n_5 + n_6 + n_7 + n_8)}{n_1 + 2n_2 + 3n_3 + 4n_4 + 5n_5 + 6n_6 + 7n_7 + 8n_8} \tag{67e}$$

$$t_6 = t_{G5} = \frac{10(n_3 + n_4 + n_5 + n_6 + n_7 + n_8)}{n_1 + 2n_2 + 3n_3 + 4n_4 + 5n_5 + 6n_6 + 7n_7 + 8n_8} \tag{67f}$$

$$t_7 = t_{G6} = \frac{10(n_2 + n_3 + n_4 + n_5 + n_6 + n_7)}{n_1 + 2n_2 + 3n_3 + 4n_4 + 5n_5 + 6n_6 + 7n_7 + 8n_8} \tag{67g}$$

7.3.22.2 8+2: GWGGGG

$n_1 = m(10 - 5t_2 - t_3 - t_4 - t_5 - 2t_6)$
$n_2 = m(2t_2 - t_1 + t_6 - t_5)$
$n_3 = m(t_5 - t_4)$
$n_4 = m(t_4 - t_3)$
$n_5 = m(t_3 - t_2)$
$n_6 = m(t_2 - t_1)$
$n_7 = 0$
$n_8 = mt_1$

$$t_1 = t_{G1} = \frac{10n_8}{n_1 + 2n_2 + 3n_3 + 4n_4 + 5n_5 + 6n_6 + 8n_8} \tag{68a}$$

$$t_2 = t_W = \frac{10(n_6 + n_8)}{n_1 + 2n_2 + 3n_3 + 4n_4 + 5n_5 + 6n_6 + 8n_8} \tag{68b}$$

$$t_3 = t_{G2} = \frac{10(n_5 + n_6 + n_8)}{n_1 + 2n_2 + 3n_3 + 4n_4 + 5n_5 + 6n_6 + 8n_8} \tag{68c}$$

$$t_4 = t_{G3} = \frac{10(n_4 + n_5 + n_6 + n_8)}{n_1 + 2n_2 + 3n_3 + 4n_4 + 5n_5 + 6n_6 + 8n_8} \tag{68d}$$

$$t_5 = t_{G4} = \frac{10(n_3 + n_4 + n_5 + n_6 + n_8)}{n_1 + 2n_2 + 3n_3 + 4n_4 + 5n_5 + 6n_6 + 8n_8} \tag{68e}$$

$$t_6 = t_{G5} = \frac{10(n_2 + n_3 + n_4 + n_5 - n_6)}{n_1 + 2n_2 + 3n_3 + 4n_4 + 5n_5 + 6n_6 + 8n_8} \tag{68f}$$

7.3.22.3 8+2: GGWGG

$n_1 = m(10 - 7t_3 - t_4 - 2t_5)$
$n_2 = m(3t_3 - t_1 - t_2 + t_5 - t_4)$
$n_3 = m(t_4 - t_3)$
$n_4 = m(t_3 - t_2)$
$n_5 = 0$
$n_6 = m(t_2 - t_1)$
$n_7 = 0$
$n_8 = mt_1$

$$t_1 = t_{G1} = \frac{10n_8}{n_1 + 2n_2 + 3n_3 + 4n_4 + 6n_6 + 8n_8} \tag{69a}$$

$$t_2 = t_{G2} = \frac{10(n_6 + n_8)}{n_1 + 2n_2 + 3n_3 + 4n_4 + 6n_6 + 8n_8} \tag{69b}$$

$$t_3 = t_W = \frac{10(n_4 + n_6 + n_8)}{n_1 + 2n_2 + 3n_3 + 4n_4 + 6n_6 + 8n_8} \tag{69c}$$

$$t_4 = t_{G3} = \frac{10(n_3 + n_4 + n_6 + n_8)}{n_1 + 2n_2 + 3n_3 + 4n_4 + 6n_6 + 8n_8} \tag{69d}$$

$$t_5 = t_{G4} = \frac{10(n_2 + n_3 - 2n_4 - n_6)}{n_1 + 2n_2 + 3n_3 + 4n_4 + 6n_6 + 8n_8} \tag{69e}$$

7.3.22.4 8+2: GGGW

$n_1 = m(10 - 10t_4)$
$n_2 = m(5t_4 - t_1 - t_2 - 2t_3)$
$n_3 = 0$
$n_4 = m(t_3 - t_2)$
$n_5 = 0$
$n_6 = m(t_2 - t_1)$
$n_7 = 0$
$n_8 = mt_1$

$$t_1 = t_{G1} = \frac{10n_8}{n_1 + 2n_2 + 4n_4 + 6n_6 + 8n_8} \tag{70a}$$

$$t_2 = t_{G2} = \frac{10(n_6 + n_8)}{n_1 + 2n_2 + 4n_4 + 6n_6 + 8n_8} \tag{70b}$$

$$t_3 = t_{G3} = \frac{10(n_4 + n_6 + n_8)}{n_1 + 2n_2 + 4n_4 + 6n_6 + 8n_8} \tag{70c}$$

$$t_4 = t_W = \frac{2(n_2 + 2n_4 + 3n_6 + 4n_8)}{n_1 + 2n_2 + 4n_4 + 6n_6 + 8n_8} \tag{70d}$$

7.3.23 9+0

7.3.23.1 9+0: WGGGGGGG

$n_1 = m(9 - t_1 - t_2 - t_3 - t_4 - t_5 - t_6 - t_7 - 2t_8)$
$n_2 = m(t_8 - t_7)$
$n_3 = m(t_7 - t_6)$
$n_4 = m(t_6 - t_5)$
$n_5 = m(t_5 - t_4)$
$n_6 = m(t_4 - t_3)$
$n_7 = m(t_3 - t_2)$
$n_8 = m(t_2 - t_1)$
$n_9 = mt_1$

$$t_1 = t_W = \frac{9n_9}{n_1 + 2n_2 + 3n_3 + 4n_4 + 5n_5 + 6n_6 + 7n_7 + 8n_8 + 9n_9} \quad (71a)$$

$$t_2 = t_{G1} = \frac{9(n_8 + n_9)}{n_1 + 2n_2 + 3n_3 + 4n_4 + 5n_5 + 6n_6 + 7n_7 + 8n_8 + 9n_9} \quad (71b)$$

$$t_3 = t_{G2} = \frac{9(n_7 + n_8 + n_9)}{n_1 + 2n_2 + 3n_3 + 4n_4 + 5n_5 + 6n_6 + 7n_7 + 8n_8 + 9n_9} \quad (71c)$$

$$t_4 = t_{G3} = \frac{9(n_6 + n_7 + n_8 + n_9)}{n_1 + 2n_2 + 3n_3 + 4n_4 + 5n_5 + 6n_6 + 7n_7 + 8n_8 + 9n_9} \quad (71d)$$

$$t_5 = t_{G4} = \frac{9(n_5 + n_6 + n_7 + n_8 + n_9)}{n_1 + 2n_2 + 3n_3 + 4n_4 + 5n_5 + 6n_6 + 7n_7 + 8n_8 + 9n_9} \quad (71e)$$

$$t_6 = t_{G5} = \frac{9(n_4 + n_5 + n_6 + n_7 + n_8 + n_9)}{n_1 + 2n_2 + 3n_3 + 4n_4 + 5n_5 + 6n_6 + 7n_7 + 8n_8 + 9n_9} \quad (71f)$$

$$t_7 = t_{G6} = \frac{9(n_3 + n_4 + n_5 + n_6 + n_7 + n_8 + n_9)}{n_1 + 2n_2 + 3n_3 + 4n_4 + 5n_5 + 6n_6 + 7n_7 + 8n_8 + 9n_9} \quad (71g)$$

$$t_8 = t_{G7} = \frac{9(n_2 + n_3 + n_4 + n_5 + n_6 + n_7 + n_8 + n_9)}{n_1 + 2n_2 + 3n_3 + 4n_4 + 5n_5 + 6n_6 + 7n_7 + 8n_8 + 9n_9} \quad (71h)$$

7.3.23.2 9+0: GWGGGGG

$n_1 = m(9 - 3t_2 - t_3 - t_4 - t_5 - t_6 - 2t_7)$
$n_2 = m(t_2 - t_1 + t_7 - t_6)$
$n_3 = m(t_6 - t_5)$
$n_4 = m(t_5 - t_4)$
$n_5 = m(t_4 - t_3)$
$n_6 = m(t_3 - t_2)$
$n_7 = m(t_2 - t_1)$
$n_8 = 0$
$n_9 = mt_1$

$$t_1 = t_{G1} = \frac{9n_9}{n_1 + 2n_2 + 3n_3 + 4n_4 + 5n_5 + 6n_6 + 7n_7 + 9n_9} \quad (72a)$$

$$t_2 = t_W = \frac{9(n_7 + n_9)}{n_1 + 2n_2 + 3n_3 + 4n_4 + 5n_5 + 6n_6 + 7n_7 + 9n_9} \quad (72b)$$

$$t_3 = t_{G2} = \frac{9(n_6 + n_7 + n_9)}{n_1 + 2n_2 + 3n_3 + 4n_4 + 5n_5 + 6n_6 + 7n_7 + 9n_9} \quad (72c)$$

$$t_4 = t_{G3} = \frac{9(n_5 + n_6 + n_7 + n_9)}{n_1 + 2n_2 + 3n_3 + 4n_4 + 5n_5 + 6n_6 + 7n_7 + 9n_9} \quad (72d)$$

$$t_5 = t_{G4} = \frac{9(n_4 + n_5 + n_6 + n_7 + n_9)}{n_1 + 2n_2 + 3n_3 + 4n_4 + 5n_5 + 6n_6 + 7n_7 + 9n_9} \tag{72e}$$

$$t_6 = t_{G5} = \frac{9(n_3 + n_4 + n_5 + n_6 + n_7 + n_9)}{n_1 + 2n_2 + 3n_3 + 4n_4 + 5n_5 + 6n_6 + 7n_7 + 9n_9} \tag{72f}$$

$$t_7 = t_{G6} = \frac{9(n_2 + n_3 + n_4 + n_5 + n_6 + n_9)}{n_1 + 2n_2 + 3n_3 + 4n_4 + 5n_5 + 6n_6 + 7n_7 + 9n_9} \tag{72g}$$

7.3.23.3 9+0: GGWGGG

$n_1 = m(9 - 5t_3 - t_4 - t_5 - 2t_6)$
$n_2 = m(2t_3 - t_2 - t_1 - t_5 + t_6)$
$n_3 = m(t_5 - t_4)$
$n_4 = m(t_4 - t_3)$
$n_5 = m(t_3 - t_2)$
$n_6 = 0$
$n_7 = m(t_2 - t_1)$
$n_8 = 0$
$n_9 = mt_1$

$$t_1 = t_{G1} = \frac{9n_9}{n_1 + 2n_2 + 3n_3 + 4n_4 + 5n_5 + 7n_7 + 9n_9} \tag{73a}$$

$$t_2 = t_{G2} = \frac{9(n_7 + n_9)}{n_1 + 2n_2 + 3n_3 + 4n_4 + 5n_5 + 7n_7 + 9n_9} \tag{73b}$$

$$t_3 = t_W = \frac{9(n_5 + n_7 + n_9)}{n_1 + 2n_2 + 3n_3 + 4n_4 + 5n_5 + 7n_7 + 9n_9} \tag{73c}$$

$$t_4 = t_{G3} = \frac{9(n_4 + n_5 + n_7 + n_9)}{n_1 + 2n_2 + 3n_3 + 4n_4 + 5n_5 + 7n_7 + 9n_9} \tag{73d}$$

$$t_5 = t_{G4} = \frac{9(n_3 + n_4 + n_5 + n_7 + n_9)}{n_1 + 2n_2 + 3n_3 + 4n_4 + 5n_5 + 7n_7 + 9n_9} \tag{73e}$$

$$t_6 = t_{G5} = \frac{9(n_2 + n_3 + n_4 - n_5 + n_9)}{n_1 + 2n_2 + 3n_3 + 4n_4 + 5n_5 + 7n_7 + 9n_9} \tag{73f}$$

7.3.23.4 9+0: GGGWG

$n_1 = m(9 - 7t_4 - 2t_5)$
$n_2 = m(t_5 - t_1 - t_2 - t_3 + 2t_4)$
$n_3 = m(t_4 - t_3)$
$n_4 = 0$
$n_5 = m(t_3 - t_2)$
$n_6 = 0$
$n_7 = m(t_2 - t_1)$
$n_8 = 0$
$n_9 = mt_1$

$$t_1 = t_{G1} = \frac{9n_9}{n_1 + 2n_2 + 3n_3 + 5n_5 + 7n_7 + 9n_9} \tag{74a}$$

$$t_2 = t_{G2} = \frac{9(n_7 + n_9)}{n_1 + 2n_2 + 3n_3 + 5n_5 + 7n_7 + 9n_9} \tag{74b}$$

$$t_3 = t_{G3} = \frac{9(n_5 + n_7 + n_9)}{n_1 + 2n_2 + 3n_3 + 5n_5 + 7n_7 + 9n_9} \tag{74c}$$

$$t_4 = t_W = \frac{9(n_3 + n_5 + n_7 + n_9)}{n_1 + 2n_2 + 3n_3 + 5n_5 + 7n_7 + 9n_9} \tag{74d}$$

$$t_5 = t_{G4} = \frac{9(n_2 - 2n_3 - n_5 + n_9)}{n_1 + 2n_2 + 3n_3 + 5n_5 + 7n_7 + 9n_9} \tag{74e}$$

7.3.24 9+1

7.3.24.1 9+1: WGGGGGGG

$n_1 = m(10 - t_1 - t_2 - t_3 - t_4 - t_5 - t_6 - t_7 - 2t_8)$
$n_2 = m(t_8 - t_7)$
$n_3 = m(t_7 - t_6)$
$n_4 = m(t_6 - t_5)$
$n_5 = m(t_5 - t_4)$
$n_6 = m(t_4 - t_3)$
$n_7 = m(t_3 - t_2)$
$n_8 = m(t_2 - t_1)$
$n_9 = mt_1$

$$t_1 = t_W = \frac{10n_9}{n_1 + 2n_2 + 3n_3 + 4n_4 + 5n_5 + 6n_6 + 7n_7 + 8n_8 + 9n_9} \tag{75a}$$

$$t_2 = t_{G1} = \frac{10(n_8 + n_9)}{n_1 + 2n_2 + 3n_3 + 4n_4 + 5n_5 + 6n_6 + 7n_7 + 8n_8 + 9n_9} \tag{75b}$$

$$t_3 = t_{G2} = \frac{10(n_7 + n_8 + n_9)}{n_1 + 2n_2 + 3n_3 + 4n_4 + 5n_5 + 6n_6 + 7n_7 + 8n_8 + 9n_9} \tag{75c}$$

$$t_4 = t_{G3} = \frac{10(n_6 + n_7 + n_8 + n_9)}{n_1 + 2n_2 + 3n_3 + 4n_4 + 5n_5 + 6n_6 + 7n_7 + 8n_8 + 9n_9} \tag{75d}$$

$$t_5 = t_{G4} = \frac{10(n_5 + n_6 + n_7 + n_8 + n_9)}{n_1 + 2n_2 + 3n_3 + 4n_4 + 5n_5 + 6n_6 + 7n_7 + 8n_8 + 9n_9} \tag{75e}$$

$$t_6 = t_{G5} = \frac{10(n_4 + n_5 + n_6 + n_7 + n_8 + n_9)}{n_1 + 2n_2 + 3n_3 + 4n_4 + 5n_5 + 6n_6 + 7n_7 + 8n_8 + 9n_9} \tag{75f}$$

$$t_7 = t_{G6} = \frac{10(n_3 + n_4 + n_5 + n_6 + n_7 + n_8 + n_9)}{n_1 + 2n_2 + 3n_3 + 4n_4 + 5n_5 + 6n_6 + 7n_7 + 8n_8 + 9n_9} \tag{75g}$$

$$t_8 = t_{G7} = \frac{10(n_2 + n_3 + n_4 + n_5 + n_6 + n_7 + n_8 + n_9)}{n_1 + 2n_2 + 3n_3 + 4n_4 + 5n_5 + 6n_6 + 7n_7 + 8n_8 + 9n_9} \tag{75h}$$

7.3.24.2 9+1: GWGGGGG

$n_1 = m(10 - 3t_2 - t_3 - t_4 - t_5 - t_6 - 2t_7)$
$n_2 = m(t_2 - t_1 + t_7 - t_6)$
$n_3 = m(t_6 - t_5)$
$n_4 = m(t_5 - t_4)$
$n_5 = m(t_4 - t_3)$
$n_6 = m(t_3 - t_2)$
$n_7 = m(t_2 - t_1)$
$n_8 = 0$
$n_9 = mt_1$

$$t_1 = t_{G1} = \frac{10n_9}{n_1 + 2n_2 + 3n_3 + 4n_4 + 5n_5 + 6n_6 + 7n_7 + 9n_9} \tag{76a}$$

$$t_2 = t_W = \frac{10(n_7 + n_9)}{n_1 + 2n_2 + 3n_3 + 4n_4 + 5n_5 + 6n_6 + 7n_7 + 9n_9} \tag{76b}$$

$$t_3 = t_{G2} = \frac{10(n_6 + n_7 + n_9)}{n_1 + 2n_2 + 3n_3 + 4n_4 + 5n_5 + 6n_6 + 7n_7 + 9n_9} \tag{76c}$$

$$t_4 = t_{G3} = \frac{10(n_5 + n_6 + n_7 + n_9)}{n_1 + 2n_2 + 3n_3 + 4n_4 + 5n_5 + 6n_6 + 7n_7 + 9n_9} \tag{76d}$$

$$t_5 = t_{G4} = \frac{10(n_4 + n_5 + n_6 + n_7 + n_9)}{n_1 + 2n_2 + 3n_3 + 4n_4 + 5n_5 + 6n_6 + 7n_7 + 9n_9} \tag{76e}$$

$$t_6 = t_{G5} = \frac{10(n_3 + n_4 + n_5 + n_6 + n_7 + n_9)}{n_1 + 2n_2 + 3n_3 + 4n_4 + 5n_5 + 6n_6 + 7n_7 + 9n_9} \tag{76f}$$

$$t_7 = t_{G6} = \frac{10(n_2 + n_3 + n_4 + n_5 + n_6 + n_9)}{n_1 + 2n_2 + 3n_3 + 4n_4 + 5n_5 + 6n_6 + 7n_7 + 9n_9} \tag{76g}$$

7.3.24.3 9+1: GGWGGG

$n_1 = m(10 - 5t_3 - t_4 - t_5 - 2t_6)$
$n_2 = m(2t_3 - t_1 - t_2 + t_6 - t_5)$
$n_3 = m(t_5 - t_4)$
$n_4 = m(t_4 - t_3)$
$n_5 = m(t_3 - t_2)$
$n_6 = 0$
$n_7 = m(t_2 - t_1)$
$n_8 = 0$
$n_9 = mt_1$

$$t_1 = t_{G1} = \frac{10n_9}{n_1 + 2n_2 + 3n_3 + 4n_4 + 5n_5 + 7n_7 + 9n_9} \tag{77a}$$

$$t_2 = t_{G2} = \frac{10(n_7 + n_9)}{n_1 + 2n_2 + 3n_3 + 4n_4 + 5n_5 + 7n_7 + 9n_9} \tag{77b}$$

$$t_3 = t_W = \frac{10(n_5 + n_7 + n_9)}{n_1 + 2n_2 + 3n_3 + 4n_4 + 5n_5 + 7n_7 + 9n_9} \tag{77c}$$

$$t_4 = t_{G3} = \frac{10(n_4 + n_5 + n_7 + n_9)}{n_1 + 2n_2 + 3n_3 + 4n_4 + 5n_5 + 7n_7 + 9n_9} \tag{77d}$$

$$t_5 = t_{G4} = \frac{10(n_3 + n_4 + n_5 + n_7 + n_9)}{n_1 + 2n_2 + 3n_3 + 4n_4 + 5n_5 + 7n_7 + 9n_9} \tag{77e}$$

$$t_6 = t_{G5} = \frac{10(n_2 + n_3 + n_4 - n_5 + n_9)}{n_1 + 2n_2 + 3n_3 + 4n_4 + 5n_5 + 7n_7 + 9n_9} \tag{77f}$$

7.3.24.4 9+1: GGGWG

$n_1 = m(10 - 7t_4 - 2t_5)$
$n_2 = m(2t_4 - t_1 - t_2 - t_3 + t_5)$
$n_3 = m(t_4 - t_3)$
$n_4 = 0$
$n_5 = m(t_3 - t_2)$
$n_6 = 0$
$n_7 = m(t_2 - t_1)$
$n_8 = 0$
$n_9 = mt_1$

$$t_1 = t_{G1} = \frac{10n_9}{n_1 + 2n_2 + 3n_3 + 5n_5 + 7n_7 + 9n_9} \tag{78a}$$

$$t_2 = t_{G2} = \frac{10(n_7 + n_9)}{n_1 + 2n_2 + 3n_3 + 5n_5 + 7n_7 + 9n_9} \tag{78b}$$

$$t_3 = t_{G3} = \frac{10(n_5 + n_7 + n_9)}{n_1 + 2n_2 + 3n_3 + 5n_5 + 7n_7 + 9n_9} \tag{78c}$$

$$t_4 = t_W = \frac{10(n_3 + n_5 + n_7 + n_9)}{n_1 + 2n_2 + 3n_3 + 5n_5 + 7n_7 + 9n_9} \tag{78d}$$

$$t_5 = t_{G4} = \frac{10(n_2 - 2n_3 - n_5 + n_9)}{n_1 + 2n_2 + 3n_3 + 5n_5 + 7n_7 + 9n_9} \tag{78e}$$

7.3.25 9+2

7.3.25.1 9+2: WGGGGGGG

$n_1 = m(11 - 3t_1 - t_2 - t_3 - t_4 - t_5 - t_6 - t_7 - 2t_8)$
$n_2 = m(t_1 - t_7 + t_8)$
$n_3 = m(t_7 - t_6)$
$n_4 = m(t_6 - t_5)$
$n_5 = m(t_5 - t_4)$
$n_6 = m(t_4 - t_3)$
$n_7 = m(t_3 - t_2)$
$n_8 = m(t_2 - t_1)$

$$t_1 = t_W = \frac{11n_9}{n_1 + 2n_2 + 3n_3 + 4n_4 + 5n_5 + 6n_6 + 7n_7 + 8n_8 + 9n_9} \tag{79a}$$

$$t_2 = t_{G1} = \frac{11(n_8 + n_9)}{n_1 + 2n_2 + 3n_3 + 4n_4 + 5n_5 + 6n_6 + 7n_7 + 8n_8 + 9n_9} \tag{79b}$$

$$t_3 = t_{G2} = \frac{11(n_7 + n_8 + n_9)}{n_1 + 2n_2 + 3n_3 + 4n_4 + 5n_5 + 6n_6 + 7n_7 + 8n_8 + 9n_9} \tag{79c}$$

$$t_4 = t_{G3} = \frac{11(n_6 + n_7 + n_8 + n_9)}{n_1 + 2n_2 + 3n_3 + 4n_4 + 5n_5 + 6n_6 + 7n_7 + 8n_8 + 9n_9} \tag{79d}$$

$$t_5 = t_{G4} = \frac{11(n_5 + n_6 + n_7 + n_8 + n_9)}{n_1 + 2n_2 + 3n_3 + 4n_4 + 5n_5 + 6n_6 + 7n_7 + 8n_8 + 9n_9} \tag{79e}$$

$$t_6 = t_{G5} = \frac{11(n_4 + n_5 + n_6 + n_7 + n_8 + n_9)}{n_1 + 2n_2 + 3n_3 + 4n_4 + 5n_5 + 6n_6 + 7n_7 + 8n_8 + 9n_9} \tag{79f}$$

$$t_7 = t_{G6} = \frac{11(n_3 + n_4 + n_5 + n_6 + n_7 + n_8 + n_9)}{n_1 + 2n_2 + 3n_3 + 4n_4 + 5n_5 + 6n_6 + 7n_7 + 8n_8 + 9n_9} \tag{79g}$$

$$t_8 = t_{G7} = \frac{11(n_2 + n_3 + n_4 + n_5 + n_6 + n_7 + n_8 + n_9)}{n_1 + 2n_2 + 3n_3 + 4n_4 + 5n_5 + 6n_6 + 7n_7 + 8n_8 + 9n_9} \tag{79h}$$

7.3.25.2 9+2: GWGGGGG

$n_1 = m(11 - 5t_2 - t_3 - t_4 - t_5 - t_6 - 2t_7)$
$n_2 = m(-t_1 + 2t_2 - t_6 + t_7)$
$n_3 = m(t_6 - t_5)$
$n_4 = m(t_5 - t_4)$
$n_5 = m(t_4 - t_3)$
$n_6 = m(t_3 - t_2)$
$n_7 = m(t_2 - t_1)$
$n_8 = 0$
$n_9 = mt_1$

$$t_1 = t_{G1} = \frac{11n_9}{n_1 + 2n_2 + 3n_3 + 4n_4 + 5n_5 + 6n_6 + 7n_7 + 9n_9} \tag{80a}$$

$$t_2 = t_W = \frac{11(n_7 + n_9)}{n_1 + 2n_2 + 3n_3 + 4n_4 + 5n_5 + 6n_6 + 7n_7 + 9n_9} \tag{80b}$$

$$t_3 = t_{G2} = \frac{11(n_6 + n_7 + n_9)}{n_1 + 2n_2 + 3n_3 + 4n_4 + 5n_5 + 6n_6 + 7n_7 + 9n_9} \tag{80c}$$

$$t_4 = t_{G3} = \frac{11(n_5 + n_6 + n_7 + n_9)}{n_1 + 2n_2 + 3n_3 + 4n_4 + 5n_5 + 6n_6 + 7n_7 + 9n_9} \tag{80d}$$

$$t_5 = t_{G4} = \frac{11(n_4 + n_5 + n_6 + n_7 + n_9)}{n_1 + 2n_2 + 3n_3 + 4n_4 + 5n_5 + 6n_6 + 7n_7 + 9n_9} \tag{80e}$$

$$t_6 = t_{G5} = \frac{11(n_3 + n_4 + n_5 + n_6 + n_7 + n_9)}{n_1 + 2n_2 + 3n_3 + 4n_4 + 5n_5 + 6n_6 + 7n_7 + 9n_9} \quad (80f)$$

$$t_7 = t_{G6} = \frac{11(n_2 + n_3 + n_4 + n_5 + n_6 - n_7)}{n_1 + 2n_2 + 3n_3 + 4n_4 + 5n_5 + 6n_6 + 7n_7 + 9n_9} \quad (80g)$$

7.3.25.3 9+2: GGWGGG

$n_1 = m(11 - 7t_3 - t_4 - t_5 - 2t_6)$
$n_2 = m(-t_1 - t_2 + 3t_3 - t_5 + t_6)$
$n_3 = m(t_5 - t_4)$
$n_4 = m(t_4 - t_3)$
$n_5 = m(t_3 - t_2)$
$n_6 = 0$
$n_7 = m(t_2 - t_1)$
$n_8 = 0$
$n_9 = mt_1$

$$t_1 = t_{G1} = \frac{11n_9}{n_1 + 2n_2 + 3n_3 + 4n_4 + 5n_5 + 7n_7 + 9n_9} \quad (81a)$$

$$t_2 = t_{G2} = \frac{11(n_7 + n_9)}{n_1 + 2n_2 + 3n_3 + 4n_4 + 5n_5 + 7n_7 + 9n_9} \quad (81b)$$

$$t_3 = t_W = \frac{11(n_5 + n_7 + n_9)}{n_1 + 2n_2 + 3n_3 + 4n_4 + 5n_5 + 7n_7 + 9n_9} \quad (81c)$$

$$t_4 = t_{G3} = \frac{11(n_4 + n_5 + n_7 + n_9)}{n_1 + 2n_2 + 3n_3 + 4n_4 + 5n_5 + 7n_7 + 9n_9} \quad (81d)$$

$$t_5 = t_{G4} = \frac{11(n_3 + n_4 + n_5 + n_7 + n_9)}{n_1 + 2n_2 + 3n_3 + 4n_4 + 5n_5 + 7n_7 + 9n_9} \quad (81e)$$

$$t_6 = t_{G5} = \frac{11(n_2 + n_3 + n_4 - 2n_5 - n_7)}{n_1 + 2n_2 + 3n_3 + 4n_4 + 5n_5 + 7n_7 + 9n_9} \quad (81f)$$

7.3.25.4 9+2: GGGWG

$n_1 = m(11 - 9t_4 - 2t_5)$
$n_2 = m(-t_1 - t_2 - t_3 + 3t_4 + t_5)$
$n_3 = m(t_4 - t_3)$
$n_4 = 0$
$n_5 = m(t_3 - t_2)$
$n_6 = 0$
$n_7 = m(t_2 - t_1)$
$n_8 = 0$
$n_9 = mt_1$

$$t_1 = t_{G1} = \frac{11n_9}{n_1 + 2n_2 + 3n_3 + 5n_5 + 7n_7 + 9n_9} \quad (82a)$$

$$t_2 = t_{G2} = \frac{11(n_7 + n_9)}{n_1 + 2n_2 + 3n_3 + 5n_5 + 7n_7 + 9n_9} \quad (82b)$$

$$t_3 = t_{G3} = \frac{11(n_5 + n_7 + n_9)}{n_1 + 2n_2 + 3n_3 + 5n_5 + 7n_7 + 9n_9} \quad (82c)$$

$$t_4 = t_W = \frac{11(n_3 + n_5 + n_7 + n_9)}{n_1 + 2n_2 + 3n_3 + 5n_5 + 7n_7 + 9n_9} \quad (82d)$$

$$t_5 = t_{G4} = \frac{11(n_2 - 3n_3 - 2n_5 - n_7)}{n_1 + 2n_2 + 3n_3 + 5n_5 + 7n_7 + 9n_9} \quad (82e)$$

7.3.26 10+0

7.3.26.1 10+0: WGGGGGGGG

$n_1 = m(10 - t_1 - t_2 - t_3 - t_4 - t_5 - t_6 - t_7 - t_8 - 2t_9)$
$n_2 = m(t_9 - t_8)$
$n_3 = m(t_8 - t_7)$
$n_4 = m(t_7 - t_6)$
$n_5 = m(t_6 - t_5)$
$n_6 = m(t_5 - t_4)$
$n_7 = m(t_4 - t_3)$
$n_8 = m(t_3 - t_2)$
$n_9 = m(t_2 - t_1)$
$n_{10} = mt_1$

$$t_1 = t_W = \frac{10n_{10}}{n_1 + 2n_2 + 3n_3 + 4n_4 + 5n_5 + 6n_6 + 7n_7 + 8n_8 + 9n_9 + 10n_{10}} \tag{83a}$$

$$t_2 = t_{G1} = \frac{10(n_9 + n_{10})}{n_1 + 2n_2 + 3n_3 + 4n_4 + 5n_5 + 6n_6 + 7n_7 + 8n_8 + 9n_9 + 10n_{10}} \tag{83b}$$

$$t_3 = t_{G2} = \frac{10(n_8 + n_9 + n_{10})}{n_1 + 2n_2 + 3n_3 + 4n_4 + 5n_5 + 6n_6 + 7n_7 + 8n_8 + 9n_9 + 10n_{10}} \tag{83c}$$

$$t_4 = t_{G3} = \frac{10(n_7 + n_8 + n_9 + n_{10})}{n_1 + 2n_2 + 3n_3 + 4n_4 + 5n_5 + 6n_6 + 7n_7 + 8n_8 + 9n_9 + 10n_{10}} \tag{83d}$$

$$t_5 = t_{G4} = \frac{10(n_6 + n_7 + n_8 + n_9 + n_{10})}{n_1 + 2n_2 + 3n_3 + 4n_4 + 5n_5 + 6n_6 + 7n_7 + 8n_8 + 9n_9 + 10n_{10}} \tag{83e}$$

$$t_6 = t_{G5} = \frac{10(n_5 + n_6 + n_7 + n_8 + n_9 + n_{10})}{n_1 + 2n_2 + 3n_3 + 4n_4 + 5n_5 + 6n_6 + 7n_7 + 8n_8 + 9n_9 + 10n_{10}} \tag{83f}$$

$$t_7 = t_{G6} = \frac{10(n_4 + n_5 + n_6 + n_7 + n_8 + n_9 + n_{10})}{n_1 + 2n_2 + 3n_3 + 4n_4 + 5n_5 + 6n_6 + 7n_7 + 8n_8 + 9n_9 + 10n_{10}} \tag{83g}$$

$$t_8 = t_{G7} = \frac{10(n_3 + n_4 + n_5 + n_6 + n_7 + n_8 + n_9 + n_{10})}{n_1 + 2n_2 + 3n_3 + 4n_4 + 5n_5 + 6n_6 + 7n_7 + 8n_8 + 9n_9 + 10n_{10}} \tag{83h}$$

$$t_9 = t_{G8} = \frac{10(n_2 + n_3 + n_4 + n_5 + n_6 + n_7 + n_8 + n_9 + n_{10})}{n_1 + 2n_2 + 3n_3 + 4n_4 + 5n_5 + 6n_6 + 7n_7 + 8n_8 + 9n_9 + 10n_{10}} \tag{83i}$$

7.3.26.2 10+0: GWGGGGGG

$n_1 = m(10 - 3t_2 - t_3 - t_4 - t_5 - t_6 - t_7 - 2t_8)$
$n_2 = m(t_2 - t_1 + t_8 - t_7)$
$n_3 = m(t_7 - t_6)$
$n_4 = m(t_6 - t_5)$
$n_5 = m(t_5 - t_4)$
$n_6 = m(t_4 - t_3)$
$n_7 = m(t_3 - t_2)$
$n_8 = m(t_2 - t_1)$
$n_9 = 0$
$n_{10} = mt_1$

$$t_1 = t_{G1} = \frac{10n_{10}}{n_1 + 2n_2 + 3n_3 + 4n_4 + 5n_5 + 6n_6 + 7n_7 + 8n_8 + 10n_{10}} \tag{84a}$$

$$t_2 = t_W = \frac{10(n_8 + n_{10})}{n_1 + 2n_2 + 3n_3 + 4n_4 + 5n_5 + 6n_6 + 7n_7 + 8n_8 + 10n_{10}} \tag{84b}$$

$$t_3 = t_{G2} = \frac{10(n_7 + n_8 + n_{10})}{n_1 + 2n_2 + 3n_3 + 4n_4 + 5n_5 + 6n_6 + 7n_7 + 8n_8 + 10n_{10}} \tag{84c}$$

$$t_4 = t_{G3} = \frac{10(n_6 + n_7 + n_8 + n_{10})}{n_1 + 2n_2 + 3n_3 + 4n_4 + 5n_5 + 6n_6 + 7n_7 + 8n_8 + 10n_{10}} \tag{84d}$$

$$t_5 = t_{G4} = \frac{10(n_5 + n_6 + n_7 + n_8 + n_{10})}{n_1 + 2n_2 + 3n_3 + 4n_4 + 5n_5 + 6n_6 + 7n_7 + 8n_8 + 10n_{10}} \tag{84e}$$

$$t_6 = t_{G5} = \frac{10(n_4 + n_5 + n_6 + n_7 + n_8 + n_{10})}{n_1 + 2n_2 + 3n_3 + 4n_4 + 5n_5 + 6n_6 + 7n_7 + 8n_8 + 10n_{10}} \tag{84f}$$

$$t_7 = t_{G6} = \frac{10(n_3 + n_4 + n_5 + n_6 + n_7 + n_8 + n_{10})}{n_1 + 2n_2 + 3n_3 + 4n_4 + 5n_5 + 6n_6 + 7n_7 + 8n_8 + 10n_{10}} \tag{84g}$$

$$t_8 = t_{G7} = \frac{10(n_2 + n_3 + n_4 + n_5 + n_6 + n_7 + n_{10})}{n_1 + 2n_2 + 3n_3 + 4n_4 + 5n_5 + 6n_6 + 7n_7 + 8n_8 + 10n_{10}} \tag{84h}$$

7.3.26.3 10+0: GGWGGGG

$n_1 = m(10 - 5t_3 - t_4 - t_5 - t_6 - 2t_7)$
$n_2 = m(t_7 - t_1 - t_2 + 2t_3 - t_6)$
$n_3 = m(t_6 - t_5)$
$n_4 = m(t_5 - t_4)$
$n_5 = m(t_4 - t_3)$
$n_6 = m(t_3 - t_2)$
$n_7 = 0$
$n_8 = m(t_2 - t_1)$
$n_9 = 0$
$n_{10} = mt_1$

$$t_1 = t_{G1} = \frac{10n_{10}}{n_1 + 2n_2 + 3n_3 + 4n_4 + 5n_5 + 6n_6 + 8n_8 + 10n_{10}} \tag{85a}$$

$$t_2 = t_{G2} = \frac{10(n_8 + n_{10})}{n_1 + 2n_2 + 3n_3 + 4n_4 + 5n_5 + 6n_6 + 8n_8 + 10n_{10}} \tag{85b}$$

$$t_3 = t_W = \frac{10(n_6 + n_8 + n_{10})}{n_1 + 2n_2 + 3n_3 + 4n_4 + 5n_5 + 6n_6 + 8n_8 + 10n_{10}} \tag{85c}$$

$$t_4 = t_{G3} = \frac{10(n_5 + n_6 + n_8 + n_{10})}{n_1 + 2n_2 + 3n_3 + 4n_4 + 5n_5 + 6n_6 + 8n_8 + 10n_{10}} \tag{85d}$$

$$t_5 = t_{G4} = \frac{10(n_4 + n_5 + n_6 + n_8 + n_{10})}{n_1 + 2n_2 + 3n_3 + 4n_4 + 5n_5 + 6n_6 + 8n_8 + 10n_{10}} \tag{85e}$$

$$t_6 = t_{G5} = \frac{10(n_3 + n_4 + n_5 + n_6 + n_8 + n_{10})}{n_1 + 2n_2 + 3n_3 + 4n_4 + 5n_5 + 6n_6 + 8n_8 + 10n_{10}} \tag{85f}$$

$$t_7 = t_{G6} = \frac{10(n_2 + n_3 + n_4 + n_5 - n_6 + n_{10})}{n_1 + 2n_2 + 3n_3 + 4n_4 + 5n_5 + 6n_6 + 8n_8 + 10n_{10}} \tag{85g}$$

7.3.26.4 10+0: GGGWGG

$n_1 = m(10 - 7t_4 - t_5 - 2t_6)$
$n_2 = m(t_6 - t_1 - t_2 - t_3 + 3t_4 - t_5)$
$n_3 = m(t_5 - t_4)$
$n_4 = m(t_4 - t_3)$
$n_5 = 0$
$n_6 = m(t_3 - t_2)$
$n_7 = 0$
$n_8 = m(t_2 - t_1)$

$n_9 = 0$
$n_{10} = mt_1$

$$t_1 = t_{G1} = \frac{10n_{10}}{n_1 + 2n_2 + 3n_3 + 4n_4 + 6n_6 + 8n_8 + 10n_{10}} \tag{86a}$$

$$t_2 = t_{G2} = \frac{10(n_8 + n_{10})}{n_1 + 2n_2 + 3n_3 + 4n_4 + 6n_6 + 8n_8 + 10n_{10}} \tag{86b}$$

$$t_3 = t_{G3} = \frac{10(n_6 + n_8 + n_{10})}{n_1 + 2n_2 + 3n_3 + 4n_4 + 6n_6 + 8n_8 + 10n_{10}} \tag{86c}$$

$$t_4 = t_W = \frac{10(n_4 + n_6 + n_8 + n_{10})}{n_1 + 2n_2 + 3n_3 + 4n_4 + 6n_6 + 8n_8 + 10n_{10}} \tag{86d}$$

$$t_5 = t_{G4} = \frac{10(n_3 + n_4 + n_6 + n_8 + n_{10})}{n_1 + 2n_2 + 3n_3 + 4n_4 + 6n_6 + 8n_8 + 10n_{10}} \tag{86e}$$

$$t_6 = t_{G5} = \frac{10(n_2 + n_3 - 2n_4 - n_6 + n_{10})}{n_1 + 2n_2 + 3n_3 + 4n_4 + 6n_6 + 8n_8 + 10n_{10}} \tag{86f}$$

7.3.26.5 10+0: GGGGW

$n_1 = m(10 - 10t_5)$
$n_2 = m(5t_5 - t_1 - t_2 - t_3 - 2t_4)$
$n_3 = 0$
$n_4 = m(t_4 - t_3)$
$n_5 = 0$
$n_6 = m(t_3 - t_2)$
$n_7 = 0$
$n_8 = m(t_2 - t_1)$
$n_9 = 0$
$n_{10} = mt_1$

$$t_1 = t_{G1} = \frac{10n_{10}}{n_1 + 2n_2 + 4n_4 + 6n_6 + 8n_8 + 10n_{10}} \tag{87a}$$

$$t_2 = t_{G2} = \frac{10(n_8 + n_{10})}{n_1 + 2n_2 + 4n_4 + 6n_6 + 8n_8 + 10n_{10}} \tag{87b}$$

$$t_3 = t_{G3} = \frac{10(n_6 + n_8 + n_{10})}{n_1 + 2n_2 + 4n_4 + 6n_6 + 8n_8 + 10n_{10}} \tag{87c}$$

$$t_4 = t_{G4} = \frac{10(n_4 + n_6 + n_8 + n_{10})}{n_1 + 2n_2 + 4n_4 + 6n_6 + 8n_8 + 10n_{10}} \tag{87d}$$

$$t_5 = t_W = \frac{2(n_2 + 2n_4 + 3n_6 + 4n_8 + 5n_{10})}{n_1 + 2n_2 + 4n_4 + 6n_6 + 8n_8 + 10n_{10}} \tag{87e}$$

7.3.27 10+1

7.3.27.1 10+1: WGGGGGGGG

$n_1 = m(11 - t_1 - t_2 - t_3 - t_4 - t_5 - t_6 - t_7 - t_8 - 2t_9)$
$n_2 = m(t_9 - t_8)$
$n_3 = m(t_8 - t_7)$
$n_4 = m(t_7 - t_6)$
$n_5 = m(t_6 - t_5)$
$n_6 = m(t_5 - t_4)$
$n_7 = m(t_4 - t_3)$
$n_8 = m(t_3 - t_2)$
$n_9 = m(t_2 - t_1)$
$n_{10} = mt_1$

$$t_1 = t_W = \frac{11n_{10}}{n_1 + 2n_2 + 3n_3 + 4n_4 + 5n_5 + 6n_6 + 7n_7 + 8n_8 + 9n_9 + 10n_{10}} \tag{88a}$$

$$t_2 = t_{G1} = \frac{11(n_9 + n_{10})}{n_1 + 2n_2 + 3n_3 + 4n_4 + 5n_5 + 6n_6 + 7n_7 + 8n_8 + 9n_9 + 10n_{10}} \tag{88b}$$

$$t_3 = t_{G2} = \frac{11(n_8 + n_9 + n_{10})}{n_1 + 2n_2 + 3n_3 + 4n_4 + 5n_5 + 6n_6 + 7n_7 + 8n_8 + 9n_9 + 10n_{10}} \tag{88c}$$

$$t_4 = t_{G3} = \frac{11(n_7 + n_8 + n_9 + n_{10})}{n_1 + 2n_2 + 3n_3 + 4n_4 + 5n_5 + 6n_6 + 7n_7 + 8n_8 + 9n_9 + 10n_{10}} \tag{88d}$$

$$t_5 = t_{G4} = \frac{11(n_6 + n_7 + n_8 + n_9 + n_{10})}{n_1 + 2n_2 + 3n_3 + 4n_4 + 5n_5 + 6n_6 + 7n_7 + 8n_8 + 9n_9 + 10n_{10}} \tag{88e}$$

$$t_6 = t_{G5} = \frac{11(n_5 + n_6 + n_7 + n_8 + n_9 + n_{10})}{n_1 + 2n_2 + 3n_3 + 4n_4 + 5n_5 + 6n_6 + 7n_7 + 8n_8 + 9n_9 + 10n_{10}} \tag{88f}$$

$$t_7 = t_{G6} = \frac{11(n_4 + n_5 + n_6 + n_7 + n_8 + n_9 + n_{10})}{n_1 + 2n_2 + 3n_3 + 4n_4 + 5n_5 + 6n_6 + 7n_7 + 8n_8 + 9n_9 + 10n_{10}} \tag{88g}$$

$$t_8 = t_{G7} = \frac{11(n_3 + n_4 + n_5 + n_6 + n_7 + n_8 + n_9 + n_{10})}{n_1 + 2n_2 + 3n_3 + 4n_4 + 5n_5 + 6n_6 + 7n_7 + 8n_8 + 9n_9 + 10n_{10}} \tag{88h}$$

$$t_9 = t_{G8} = \frac{11(n_2 + n_3 + n_4 + n_5 + n_6 + n_7 + n_8 + n_9 + n_{10})}{n_1 + 2n_2 + 3n_3 + 4n_4 + 5n_5 + 6n_6 + 7n_7 + 8n_8 + 9n_9 + 10n_{10}} \tag{88i}$$

7.3.27.2 10+1: GWGGGGGG

$n_1 = m(11 - 3t_2 - t_3 - t_4 - t_5 - t_6 - t_7 - 2t_8)$
$n_2 = m(t_2 - t_1 + t_8 - t_7)$
$n_3 = m(t_7 - t_6)$
$n_4 = m(t_6 - t_5)$
$n_5 = m(t_5 - t_4)$
$n_6 = m(t_4 - t_3)$
$n_7 = m(t_3 - t_2)$
$n_8 = m(t_2 - t_1)$
$n_9 = 0$
$n_{10} = mt_1$

$$t_1 = t_{G1} = \frac{11n_{10}}{n_1 + 2n_2 + 3n_3 + 4n_4 + 5n_5 + 6n_6 + 7n_7 + 8n_8 + 10n_{10}} \tag{89a}$$

$$t_2 = t_W = \frac{11(n_8 + n_{10})}{n_1 + 2n_2 + 3n_3 + 4n_4 + 5n_5 + 6n_6 + 7n_7 + 8n_8 + 10n_{10}} \tag{89b}$$

$$t_3 = t_{G2} = \frac{11(n_7 + n_8 + n_{10})}{n_1 + 2n_2 + 3n_3 + 4n_4 + 5n_5 + 6n_6 + 7n_7 + 8n_8 + 10n_{10}} \tag{89c}$$

$$t_4 = t_{G3} = \frac{11(n_6 + n_7 + n_8 + n_{10})}{n_1 + 2n_2 + 3n_3 + 4n_4 + 5n_5 + 6n_6 + 7n_7 + 8n_8 + 10n_{10}} \tag{89d}$$

$$t_5 = t_{G4} = \frac{11(n_5 + n_6 + n_7 + n_8 + n_{10})}{n_1 + 2n_2 + 3n_3 + 4n_4 + 5n_5 + 6n_6 + 7n_7 + 8n_8 + 10n_{10}} \tag{89e}$$

$$t_6 = t_{G5} = \frac{11(n_4 + n_5 + n_6 + n_7 + n_8 + n_{10})}{n_1 + 2n_2 + 3n_3 + 4n_4 + 5n_5 + 6n_6 + 7n_7 + 8n_8 + 10n_{10}} \tag{89f}$$

$$t_7 = t_{G6} = \frac{11(n_3 + n_4 + n_5 + n_6 + n_7 + n_8 + n_{10})}{n_1 + 2n_2 + 3n_3 + 4n_4 + 5n_5 + 6n_6 + 7n_7 + 8n_8 + 10n_{10}} \tag{89g}$$

$$t_8 = t_{G7} = \frac{11(n_2 + n_3 + n_4 + n_5 + n_6 + n_7 + n_{10})}{n_1 + 2n_2 + 3n_3 + 4n_4 + 5n_5 + 6n_6 + 7n_7 + 8n_8 + 10n_{10}} \tag{89h}$$

7.3.27.3 10+1: GGWGGGG

$n_1 = m(11 - 5t_3 - t_4 - t_5 - t_6 - 2t_7)$
$n_2 = m(t_7 - t_1 - t_2 + 2t_3 - t_6)$
$n_3 = m(t_6 - t_5)$
$n_4 = m(t_5 - t_4)$
$n_5 = m(t_4 - t_3)$
$n_6 = m(t_3 - t_2)$
$n_7 = 0$
$n_8 = m(t_2 - t_1)$
$n_9 = 0$
$n_{10} = mt_1$

$$t_1 = t_{G1} = \frac{11n_{10}}{n_1 + 2n_2 + 3n_3 + 4n_4 + 5n_5 + 6n_6 + 8n_8 + 10n_{10}} \tag{90a}$$

$$t_2 = t_{G2} = \frac{11(n_8 + n_{10})}{n_1 + 2n_2 + 3n_3 + 4n_4 + 5n_5 + 6n_6 + 8n_8 + 10n_{10}} \tag{90b}$$

$$t_3 = t_W = \frac{11(n_6 + n_8 + n_{10})}{n_1 + 2n_2 + 3n_3 + 4n_4 + 5n_5 + 6n_6 + 8n_8 + 10n_{10}} \tag{90c}$$

$$t_4 = t_{G3} = \frac{11(n_5 + n_6 + n_8 + n_{10})}{n_1 + 2n_2 + 3n_3 + 4n_4 + 5n_5 + 6n_6 + 8n_8 + 10n_{10}} \tag{90d}$$

$$t_5 = t_{G4} = \frac{11(n_4 + n_5 + n_6 + n_8 + n_{10})}{n_1 + 2n_2 + 3n_3 + 4n_4 + 5n_5 + 6n_6 + 8n_8 + 10n_{10}} \tag{90e}$$

$$t_6 = t_{G5} = \frac{11(n_3 + n_4 + n_5 + n_6 + n_8 + n_{10})}{n_1 + 2n_2 + 3n_3 + 4n_4 + 5n_5 + 6n_6 + 8n_8 + 10n_{10}} \tag{90f}$$

$$t_7 = t_{G6} = \frac{11(n_2 + n_3 + n_4 + n_5 - n_6 + n_{10})}{n_1 + 2n_2 + 3n_3 + 4n_4 + 5n_5 + 6n_6 + 8n_8 + 10n_{10}} \tag{90g}$$

7.3.27.4 10+1: GGGWGG

$n_1 = m(11 - 7t_4 - t_5 - 2t_6)$
$n_2 = m(t_6 - t_1 - t_2 - t_3 + 3t_4 - t_5)$
$n_3 = m(t_5 - t_4)$
$n_4 = m(t_4 - t_3)$
$n_5 = 0$
$n_6 = m(t_3 - t_2)$
$n_7 = 0$
$n_8 = m(t_2 - t_1)$

$n_9 = 0$
$n_{10} = mt_1$

$$t_1 = t_{G1} = \frac{11n_{10}}{n_1 + 2n_2 + 3n_3 + 4n_4 + 6n_6 + 8n_8 + 10n_{10}} \tag{91a}$$

$$t_2 = t_{G2} = \frac{11(n_8 + n_{10})}{n_1 + 2n_2 + 3n_3 + 4n_4 + 6n_6 + 8n_8 + 10n_{10}} \tag{91b}$$

$$t_3 = t_{G3} = \frac{11(n_6 + n_8 + n_{10})}{n_1 + 2n_2 + 3n_3 + 4n_4 + 6n_6 + 8n_8 + 10n_{10}} \tag{91c}$$

$$t_4 = t_W = \frac{11(n_4 + n_6 + n_8 + n_{10})}{n_1 + 2n_2 + 3n_3 + 4n_4 + 6n_6 + 8n_8 + 10n_{10}} \tag{91d}$$

$$t_5 = t_{G4} = \frac{11(n_3 + n_4 + n_6 + n_8 + n_{10})}{n_1 + 2n_2 + 3n_3 + 4n_4 + 6n_6 + 8n_8 + 10n_{10}} \tag{91e}$$

$$t_6 = t_{G5} = \frac{11(n_2 + n_3 - 2n_4 - n_6 + n_8)}{n_1 + 2n_2 + 3n_3 + 4n_4 + 6n_6 + 8n_8 + 10n_{10}} \tag{91f}$$

7.3.27.5 10+1: GGGGW

$n_1 = m(11 - 10t_5)$
$n_2 = m(5t_5 - t_1 - t_2 - t_3 - 2t_4)$
$n_3 = 0$
$n_4 = m(t_4 - t_3)$
$n_5 = 0$
$n_6 = m(t_3 - t_2)$
$n_7 = 0$
$n_8 = m(t_2 - t_1)$
$n_9 = 0$
$n_{10} = mt_1$

$$t_1 = t_{G1} = \frac{11n_{10}}{n_1 + 2n_2 + 4n_4 + 6n_6 + 8n_8 + 10n_{10}} \tag{92a}$$

$$t_2 = t_{G2} = \frac{11(n_8 + n_{10})}{n_1 + 2n_2 + 4n_4 + 6n_6 + 8n_8 + 10n_{10}} \tag{92b}$$

$$t_3 = t_{G3} = \frac{11(n_6 + n_8 + n_{10})}{n_1 + 2n_2 + 4n_4 + 6n_6 + 8n_8 + 10n_{10}} \tag{92c}$$

$$t_4 = t_{G4} = \frac{11(n_4 + n_6 + n_8 + n_{10})}{n_1 + 2n_2 + 4n_4 + 6n_6 + 8n_8 + 10n_{10}} \tag{92d}$$

$$t_5 = t_W = \frac{11(n_2 + 2n_4 + 3n_6 + 4n_8 + 5n_{10})}{5(n_1 + 2n_2 + 4n_4 + 6n_6 + 8n_8 + 10n_{10})} \tag{92e}$$

7.3.28 10+2

7.3.28.1 10+2: WGGGGGGGG

$n_1 = m(12 - 3t_1 - t_2 - t_3 - t_4 - t_5 - t_6 - t_7 - t_8 - 2t_9)$
$n_2 = m(t_1 - t_8 + t_9)$
$n_3 = m(t_8 - t_7)$
$n_4 = m(t_7 - t_6)$
$n_5 = m(t_6 - t_5)$
$n_6 = m(t_5 - t_4)$
$n_7 = m(t_4 - t_3)$
$n_8 = m(t_3 - t_2)$
$n_9 = m(t_2 - t_1)$
$n_{10} = mt_1$

$$t_1 = t_W = \frac{12n_{10}}{n_1 + 2n_2 + 3n_3 + 4n_4 + 5n_5 + 6n_6 + 7n_7 + 8n_8 + 9n_9 + 10n_{10}} \tag{93a}$$

$$t_2 = t_{G1} = \frac{12(n_9 + n_{10})}{n_1 + 2n_2 + 3n_3 + 4n_4 + 5n_5 + 6n_6 + 7n_7 + 8n_8 + 9n_9 + 10n_{10}} \tag{93b}$$

$$t_3 = t_{G2} = \frac{12(n_8 + n_9 + n_{10})}{n_1 + 2n_2 + 3n_3 + 4n_4 + 5n_5 + 6n_6 + 7n_7 + 8n_8 + 9n_9 + 10n_{10}} \tag{93c}$$

$$t_4 = t_{G3} = \frac{12(n_7 + n_8 + n_9 + n_{10})}{n_1 + 2n_2 + 3n_3 + 4n_4 + 5n_5 + 6n_6 + 7n_7 + 8n_8 + 9n_9 + 10n_{10}} \tag{93d}$$

$$t_5 = t_{G4} = \frac{12(n_6 + n_7 + n_8 + n_9 + n_{10})}{n_1 + 2n_2 + 3n_3 + 4n_4 + 5n_5 + 6n_6 + 7n_7 + 8n_8 + 9n_9 + 10n_{10}} \tag{93e}$$

$$t_6 = t_{G5} = \frac{12(n_5 + n_6 + n_7 + n_8 + n_9 + n_{10})}{n_1 + 2n_2 + 3n_3 + 4n_4 + 5n_5 + 6n_6 + 7n_7 + 8n_8 + 9n_9 + 10n_{10}} \tag{93f}$$

$$t_7 = t_{G6} = \frac{12(n_4 + n_5 + n_6 + n_7 + n_8 + n_9 + n_{10})}{n_1 + 2n_2 + 3n_3 + 4n_4 + 5n_5 + 6n_6 + 7n_7 + 8n_8 + 9n_9 + 10n_{10}} \tag{93g}$$

$$t_8 = t_{G7} = \frac{12(n_3 + n_4 + n_5 + n_6 + n_7 + n_8 + n_9 + n_{10})}{n_1 + 2n_2 + 3n_3 + 4n_4 + 5n_5 + 6n_6 + 7n_7 + 8n_8 + 9n_9 + 10n_{10}} \tag{93h}$$

$$t_9 = t_{G8} = \frac{12(n_2 + n_3 + n_4 + n_5 + n_6 + n_7 + n_8 + n_9)}{n_1 + 2n_2 + 3n_3 + 4n_4 + 5n_5 + 6n_6 + 7n_7 + 8n_8 + 9n_9 + 10n_{10}} \tag{93i}$$

7.3.28.2 10+2: GWGGGGGG

$n_1 = m(12 - 5t_2 - t_3 - t_4 - t_5 - t_6 - t_7 - 2t_8)$
$n_2 = m(-t_1 + 2t_2 - t_7 + t_8)$
$n_3 = m(t_7 - t_6)$
$n_4 = m(t_6 - t_5)$
$n_5 = m(t_5 - t_4)$
$n_6 = m(t_4 - t_3)$
$n_7 = m(t_3 - t_2)$
$n_8 = m(t_2 - t_1)$
$n_9 = 0$
$n_{10} = mt_1$

$$t_1 = t_{G1} = \frac{12n_{10}}{n_1 + 2n_2 + 3n_3 + 4n_4 + 5n_5 + 6n_6 + 7n_7 + 8n_8 + 10n_{10}} \tag{94a}$$

$$t_2 = t_W = \frac{12(n_8 + n_{10})}{n_1 + 2n_2 + 3n_3 + 4n_4 + 5n_5 + 6n_6 + 7n_7 + 8n_8 + 10n_{10}} \tag{94b}$$

$$t_3 = t_{G2} = \frac{12(n_7 + n_8 + n_{10})}{n_1 + 2n_2 + 3n_3 + 4n_4 + 5n_5 + 6n_6 + 7n_7 + 8n_8 + 10n_{10}} \quad (94c)$$

$$t_4 = t_{G3} = \frac{12(n_6 + n_7 + n_8 + n_{10})}{n_1 + 2n_2 + 3n_3 + 4n_4 + 5n_5 + 6n_6 + 7n_7 + 8n_8 + 10n_{10}} \quad (94d)$$

$$t_5 = t_{G4} = \frac{12(n_5 + n_6 + n_7 + n_8 + n_{10})}{n_1 + 2n_2 + 3n_3 + 4n_4 + 5n_5 + 6n_6 + 7n_7 + 8n_8 + 10n_{10}} \quad (94e)$$

$$t_6 = t_{G5} = \frac{12(n_4 + n_5 + n_6 + n_7 + n_8 + n_{10})}{n_1 + 2n_2 + 3n_3 + 4n_4 + 5n_5 + 6n_6 + 7n_7 + 8n_8 + 10n_{10}} \quad (94f)$$

$$t_7 = t_{G6} = \frac{12(n_3 + n_4 + n_5 + n_6 + n_7 + n_8 + n_{10})}{n_1 + 2n_2 + 3n_3 + 4n_4 + 5n_5 + 6n_6 + 7n_7 + 8n_8 + 10n_{10}} \quad (94g)$$

$$t_8 = t_{G7} = \frac{12(n_2 + n_3 + n_4 + n_5 + n_6 + n_7 - n_8)}{n_1 + 2n_2 + 3n_3 + 4n_4 + 5n_5 + 6n_6 + 7n_7 + 8n_8 + 10n_{10}} \quad (94h)$$

7.3.28.3 10+2: GGWGGGG

$n_1 = m(12 - 7t_3 - t_4 - t_5 - t_6 - 2t_7)$
$n_2 = m(-t_1 - t_2 + 3t_3 - t_6 + t_7)$
$n_3 = m(t_6 - t_5)$
$n_4 = m(t_5 - t_4)$
$n_5 = m(t_4 - t_3)$
$n_6 = m(t_3 - t_2)$
$n_7 = 0$
$n_8 = m(t_2 - t_1)$
$n_9 = 0$
$n_{10} = mt_1$

$$t_1 = t_{G1} = \frac{12n_{10}}{n_1 + 2n_2 + 3n_3 + 4n_4 + 5n_5 + 6n_6 + 8n_8 + 10n_{10}} \quad (95a)$$

$$t_2 = t_{G2} = \frac{12(n_8 + n_{10})}{n_1 + 2n_2 + 3n_3 + 4n_4 + 5n_5 + 6n_6 + 8n_8 + 10n_{10}} \quad (95b)$$

$$t_3 = t_W = \frac{12(n_6 + n_8 + n_{10})}{n_1 + 2n_2 + 3n_3 + 4n_4 + 5n_5 + 6n_6 + 8n_8 + 10n_{10}} \quad (95c)$$

$$t_4 = t_{G3} = \frac{12(n_5 + n_6 + n_8 + n_{10})}{n_1 + 2n_2 + 3n_3 + 4n_4 + 5n_5 + 6n_6 + 8n_8 + 10n_{10}} \quad (95d)$$

$$t_5 = t_{G4} = \frac{12(n_4 + n_5 + n_6 + n_8 + n_{10})}{n_1 + 2n_2 + 3n_3 + 4n_4 + 5n_5 + 6n_6 + 8n_8 + 10n_{10}} \quad (95e)$$

$$t_6 = t_{G5} = \frac{12(n_3 + n_4 + n_5 + n_6 + n_8 + n_{10})}{n_1 + 2n_2 + 3n_3 + 4n_4 + 5n_5 + 6n_6 + 8n_8 + 10n_{10}} \quad (95f)$$

$$t_7 = t_{G6} = \frac{12(n_2 + n_3 + n_4 + n_5 - 2n_6 - n_8)}{n_1 + 2n_2 + 3n_3 + 4n_4 + 5n_5 + 6n_6 + 8n_8 + 10n_{10}} \quad (95g)$$

7.3.28.4 10+2: GGGWGG

$n_1 = m(12 - 9t_4 - t_5 - 2t_6)$
$n_2 = m(-t_1 - t_2 - t_3 + 4t_4 - t_5 + t_6)$
$n_3 = m(t_5 - t_4)$
$n_4 = m(t_4 - t_3)$
$n_5 = 0$
$n_6 = m(t_3 - t_2)$
$n_7 = 0$
$n_8 = m(t_2 - t_1)$

$n_9 = 0$
$n_{10} = mt_1$

$$t_1 = t_{G1} = \frac{12n_{10}}{n_1 + 2n_2 + 3n_3 + 4n_4 + 6n_6 + 8n_8 + 10n_{10}} \tag{96a}$$

$$t_2 = t_{G2} = \frac{12(n_8 + n_{10})}{n_1 + 2n_2 + 3n_3 + 4n_4 + 6n_6 + 8n_8 + 10n_{10}} \tag{96b}$$

$$t_3 = t_{G3} = \frac{12(n_6 + n_8 + n_{10})}{n_1 + 2n_2 + 3n_3 + 4n_4 + 6n_6 + 8n_8 + 10n_{10}} \tag{96c}$$

$$t_4 = t_W = \frac{12(n_4 + n_6 + n_8 + n_{10})}{n_1 + 2n_2 + 3n_3 + 4n_4 + 6n_6 + 8n_8 + 10n_{10}} \tag{96d}$$

$$t_5 = t_{G4} = \frac{12(n_3 + n_4 + n_6 + n_8 + n_{10})}{n_1 + 2n_2 + 3n_3 + 4n_4 + 6n_6 + 8n_8 + 10n_{10}} \tag{96e}$$

$$t_6 = t_{G5} = \frac{12(n_2 + n_3 - 3n_4 - 2n_6 - n_8)}{n_1 + 2n_2 + 3n_3 + 4n_4 + 6n_6 + 8n_8 + 10n_{10}} \tag{96f}$$

7.3.28.5 10+2: GGGGW

$n_1 = m(12 - 12t_5)$
$n_2 = m(6t_5 - t_1 - t_2 - t_3 - 2t_4)$
$n_3 = 0$
$n_4 = m(t_4 - t_3)$
$n_5 = 0$
$n_6 = m(t_3 - t_2)$
$n_7 = 0$
$n_8 = m(t_2 - t_1)$
$n_9 = 0$
$n_{10} = mt_1$

$$t_1 = t_{G1} = \frac{12n_{10}}{n_1 + 2n_2 + 4n_4 + 6n_6 + 8n_8 + 10n_{10}} \tag{97a}$$

$$t_2 = t_{G2} = \frac{12(n_8 + n_{10})}{n_1 + 2n_2 + 4n_4 + 6n_6 + 8n_8 + 10n_{10}} \tag{97b}$$

$$t_3 = t_{G3} = \frac{12(n_6 + n_8 + n_{10})}{n_1 + 2n_2 + 4n_4 + 6n_6 + 8n_8 + 10n_{10}} \tag{97c}$$

$$t_4 = t_{G4} = \frac{12(n_4 + n_6 + n_8 + n_{10})}{n_1 + 2n_2 + 4n_4 + 6n_6 + 8n_8 + 10n_{10}} \tag{97d}$$

$$t_5 = t_W = \frac{2(n_2 + 2n_4 + 3n_6 + 4n_8 + 5n_{10})}{n_1 + 2n_2 + 4n_4 + 6n_6 + 8n_8 + 10n_{10}} \tag{97e}$$

8 Incorrect ordering of time points

Occasionally, `AmplificationTimeR` will produce timing estimates in an incorrect order (e.g. $t_1 > t_2$). We suggest that, in some cases, this may indicate scenarios in which gains and WGD events have been followed by a loss. Considering the timing equations for a copy number state of $3+2$ in a whole genome duplicated sample without considering losses, this copy number state can only arise through a WGD event followed by a gain (Equation 28, Supplementary Figure ST2 A). In the event of a whole genome duplication followed by a gain, the total number of mutations of multiplicity 2 (n_2) would be made up of the mutations occurring before WGD on the minor allele and after WGD on the major allele, whereas multiplicity 3 mutations (n_3) would exclusively be attributed to mutations occurring on the major allele before WGD. Given the assumption that mutation rate remains constant, and both the major and the minor allele are equally likely to be mutated, it is impossible for n_3 $>n_2$ to occur.

Considering Equation 28 again, a scenario in which $t_1 > t_2$ can occur where the observed number of mutations at multiplicity 3 (n_3) is larger than the observed number of mutations at multiplicity 2 (n_2; Supplementary Figure ST2 A). In this case, $n_3 > n_2$ implies that the interval $[0, t_1]$ would have to be longer than the interval $[0, t_2]$, which is impossible. Thus, incorrectly ordered points may reflect a different order of events that includes loss of a chromosome.

A plausible alternative scenario would be a gain of the major chromosome followed by WGD creating a copy number state of $4+2$, followed by a loss of one of the major alleles, resulting in a final copy number of $3+2$ (Supplementary Figure ST2 B). Provided that the time interval from $0-t_1$ is at least twice the time between t_1 and t_2 (i.e. t_2-t_1), one would expect to observe more mutations of multiplicity 3 than multiplicity 2, while preserving $t_1 < t_2$. Unfortunately, the loss itself cannot be timed.

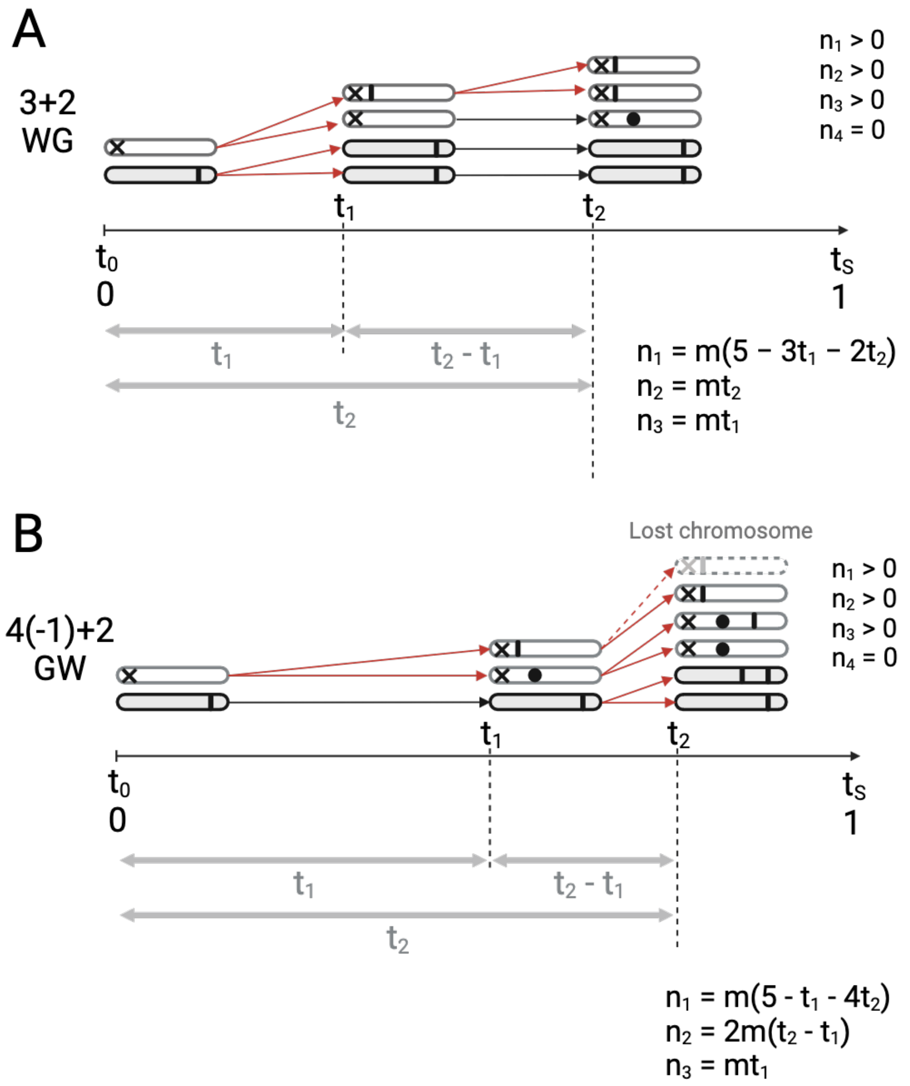

Figure ST2: A schematic representation of events resulting in a final copy number state of $3+2$ in a whole genome duplicated sample. **A.** A model in which only gains are considered, and a state of $3+2$ is achieved through a WGD followed by a gain. **B.** A model in which a chromosomal gain is followed by a WGD event, which is followed by the loss of one chromosome. n_x represents the number of mutations with multiplicity x, where x is 1, 2, 3, or 4. Red arrows indicate gain events or WGD. W=WGD, G=Gain. Image created with BioRender.com
